# Mid-infrared cross-comb spectroscopy

**DOI:** 10.1038/s41467-023-36811-7

**Published:** 2023-02-24

**Authors:** Mingchen Liu, Robert M. Gray, Luis Costa, Charles R. Markus, Arkadev Roy, Alireza Marandi

**Affiliations:** 1 Department of Electrical Engineering, California Institute of Technology, Pasadena, CA 91125 USA; 2 Division of Chemistry and Chemical Engineering, California Institute of Technology, Pasadena, CA 91125 USA

**Keywords:** Infrared spectroscopy, Mid-infrared photonics, Frequency combs

## Abstract

Dual-comb spectroscopy has been proven beneficial in molecular characterization but remains challenging in the mid-infrared region due to difficulties in sources and efficient photodetection. Here we introduce cross-comb spectroscopy, in which a mid-infrared comb is upconverted via sum-frequency generation with a near-infrared comb of a shifted repetition rate and then interfered with a spectral extension of the near-infrared comb. We measure CO_2_ absorption around 4.25 µm with a 1-µm photodetector, exhibiting a 233-cm^−1^ instantaneous bandwidth, 28000 comb lines, a single-shot signal-to-noise ratio of 167 and a figure of merit of 2.4 × 10^6^ Hz^1/2^. We show that cross-comb spectroscopy can have superior signal-to-noise ratio, sensitivity, dynamic range, and detection efficiency compared to other dual-comb-based methods and mitigate the limits of the excitation background and detector saturation. This approach offers an adaptable and powerful spectroscopic method outside the well-developed near-IR region and opens new avenues to high-performance frequency-comb-based sensing with wavelength flexibility.

## Abstract (web summary)

The authors introduce and demonstrate cross-comb spectroscopy in the mid-infrared as a variant of dual-comb spectroscopy. It provides enhanced performance and allows mid-infrared spectral information to be obtained by near-infrared detection.

## Introduction

Dual-comb spectroscopy (DCS), based on two mutually locked frequency comb (FC) sources in the same wavelength range, has become a compelling alternative to traditional Fourier-transform infrared spectroscopy (FTIR) with advantages in resolution, precision, sensitivity, speed, and bandwidth^1–3^. Over the past decade, significant efforts have focused on its extension to the mid-infrared (MIR) spectral region (3–25 µm)^4–9^, where strong molecular signatures are located, making it promising for numerous applications in physical, chemical, biological, and medical sciences or technologies. However, generating two mutually locked broadband frequency comb sources in the MIR has posed a significant challenge. In addition, photodetectors in the MIR usually suffer from lower sensitivity, higher noise, and slower response times, and generally require cooling, compared to their well-developed near-infrared (NIR) counterparts. Moreover, photodetection above 13 µm^10^ remains a significant challenge.

On the other hand, the signal-to-noise ratio (SNR), sensitivity (detection limit) and dynamic range (DR) of DCS have been limited by noise from strong excitation background^11,12^, especially for the detection of trace molecules. To detect a weaker absorption, one can apply a higher excitation power, but the stronger background signal from it will in turn decrease the dynamic range, which is an undesirable trade-off in DCS. Additionally, the sensitivity can still be limited by detector saturation. Therefore, although significant progress has been made toward broadband and high-power (>100 mW) MIR frequency combs^13–19^, the MIR DCS does not yet take full advantage of such sources since typical MIR detectors saturate at ~1 mW. Recently, some works have been demonstrated to alleviate the background issue by linear interferometry^20,21^.

To overcome those obstacles, one effective path is to upconvert the MIR FC to the NIR region using short pulses and capture the wealth of molecular information available in the MIR with NIR photodetectors. Electro-optic sampling (EOS) is one recent successful example of this approach^11,22^, in which ultrashort NIR pulses are used to directly detect the electric field of MIR pulses in the time domain. However, this method necessitates very short NIR pulses with durations shorter than the optical cycle of the carrier frequency of the MIR pulses^23,24^, whose generation and dispersion control require substantial efforts. Besides, the detection is based on field-dependent polarization rotation of the NIR sampling pulses, which adds additional components to the measurement setup. Moreover, to get higher SNR and sensitivity in EOS, some additional efforts may be required to independently tune the power and spectrum of different spectral parts of the ultrashort NIR pulses^25,26^.

In addition to EOS, one can also upconvert the MIR frequency comb using a high-power NIR continuous-wave (C.W.) laser and perform standard DCS in the NIR region^27^. Nonetheless, this method has not yet been demonstrated to exhibit a favorable signal-to-noise ratio (SNR) and bandwidth compared to direct MIR DCS, mainly owing to its low upconversion efficiency because of the low peak power of C.W. lasers. More essentially, this method is still constrained by the above-mentioned limitations of the general DCS as there is no temporal gating^22^.

In this work, we introduce cross-comb spectroscopy (CCS), which can be considered a general form of frequency-converted DCS. Specifically, we show that short-pulse CCS can fundamentally have better SNR, sensitivity, DR, and detection efficiency compared to DCS or C.W. upconversion DCS, and does not require ultrashort NIR pulses and ellipsometry like EOS. We experimentally demonstrate a MIR measurement of atmospheric CO_2_ around 4 µm with a NIR 1-µm detector, which exhibits a high temporal SNR and a high figure of merit (FOM)^12^. Moreover, in CCS, detector saturation can be circumvented since the detection process has been divided into two parts, upconversion (by a NIR local FC) and interference (with a NIR readout FC), which can be tuned independently. In addition, upon a proper setting of the power, the detection can fully utilize the DR of the detector, which does not have to be sacrificed for higher sensitivity. Provided suitable comb sources and strong upconversion capabilities are available, this method can be extended to any wavelength range and promises a superior performance that can break the limits of conventional methods, especially for longer wavelengths.

## Results

### Architecture of the CCS system

**Fig. 1:**
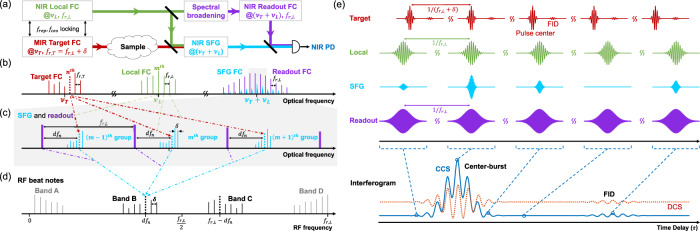
Cross-comb spectroscopy. **a** Schematic of the setup. *ν*_*L*_ and *ν*_*T*_, center optical frequencies of the NIR local FC and MIR target FCs. *f*_*ceo*_ and *f*_*rep*_, carrier–envelope offset frequency and repetition rate of a FC. *f*_*r,L*_ and *f*_*r,T*_, repetition rate of the local FC and the target FC. *δ*, difference between *f*_*r,L*_ and *f*_*r,T*_. PD, photodetector. **b**–**d** Principle of the tooth mapping in frequency domain. An example target tooth, together with its corresponding SFG teeth and RF teeth, is denoted by a dashed line to demonstrate the one-to-one mapping. n (m): index of the example tooth of the target FC (local FC). **b** Optical spectra. **c** Zoomed-in view of the grey-shadowed area in **(b)**. *df*_*n*_ denotes the distance of the SFG teeth (dashed lines) generated by the *n*_*th*_ target tooth from their respective closest readout tooth. **d** Heterodyne beat notes in the RF domain, obtained by square-law detection of the interference between the SFG FC and readout FC with a single NIR detector. Band B (C) is the result of the beating between SFG teeth with their nearest (second nearest) readout tooth, while band A (D) is the result of the beating between two SFG teeth from the same SFG group (two adjacent SFG groups). The arrows denote the optical tooth pairs in **(c)** that contribute to the dashed RF tooth. **e** CCS in time domain. In addition to the typical CCS interferogram (solid blue curve), a typical DCS interferogram (dashed red curve) is plotted for comparison. Note that this illustration describes the case where the target, local and readout FCs are all short pulses, which is not necessary for general CCS. While the DCS interferogram baseline is delay-independent since the envelopes of two pulses are delay-independent, the envelope of the SFG signal in CCS is delay-dependent, which gives a delay-dependent baseline and makes the interferogram “vertically asymmetric”. This time-domain delay-dependent baseline in CCS interferogram corresponds to band A and D in frequency domain, which can be canceled out via balanced detection. More details can be found in Supplementary Sections 1.2, 2, 3 and 5. FID, free induction decay.

The general architecture of CCS for upconversion is illustrated in Fig. 1a. The spectral information contained in the MIR (target) FC is upconverted to the NIR region via sum-frequency-generation (SFG) with a NIR (local) FC of a slightly shifted repetition rate (shifted by *δ*). The SFG output is then interfered with the spectral extension of the local FC (readout FC) to transfer the MIR information into the radio frequency (RF) domain. Like DCS, it is possible to realize a one-to-one mapping between the MIR FC teeth and the RF comb teeth, which are easily accessed with a single NIR detector and RF measurement. To obtain a tooth-resolved absorption spectrum of the target FC, the minimum required aggregate bandwidth of the local and readout FC is equal to the bandwidth of the target FC, which eliminates the need found in EOS for super-short NIR pulse generation and measurement of polarization rotation.

### One-to-one comb tooth mapping

Fig. 1 illustrates the operation principle of CCS using sum-frequency sampling. In Fig. 1b, each pair of comb teeth from the local FC (green) and target FC (red) generates an SFG tooth at a unique frequency, the set of which is referred to as the SFG FC (blue). The teeth of the SFG FC cluster into different frequency groups that are evenly spaced by the repetition rate of the local FC (*f*_*rL*_)^28^ and follow specific patterns (see Fig. 1c and Supplementary Section 2.2). Within a frequency group, the SFG teeth are separated by δ, and each tooth represents a unique tooth of the target comb. Across all SFG groups, teeth generated by the same tooth of the target comb are all at the same relative frequency position in each group. These characteristics enable one-to-one mapping from the MIR domain to the RF domain. To realize this, a readout FC (purple), which is a spectral extension of the local FC, is employed to beat with the SFG FC on a NIR photodetector. For SFG teeth that are generated by the same target tooth, they share the same unique distance to their respective closest readout tooth and are therefore mapped to the same RF tooth, which creates a unique mapping of the target tooth from the mid-IR domain to the RF domain. To explain those patterns more clearly, let us use an arbitrarily chosen target tooth as an example, labeled as the n^th^ target tooth and denoted by a dashed line in Fig. 1b. The process of its mapping from the mid-IR domain to the RF domain, including SFG and heterodyne beating, is shown by arrows in Fig. 1b-d. Like all other target teeth, it mixes with different local teeth and generates multiple SFG teeth that are distributed across SFG groups and separated by *f*_*rL*_. They share the same unique distance to their closest readout tooth, as denoted by *df*_*n*_ in Fig. 1c. Thus, the dashed tooth in RF band B is the unique mapping of the *n*_*th*_ target tooth (Fig. 1d), as is its mirror image in RF band C.

The resultant RF FC contains four distinguishable bands (Fig. 1d)^23^. While band A and D correspond to the envelope of the SFG pulses (intensity cross-correlation between the target FC and local FC) and thus lack useful spectral information, the above shows that band B (or its mirror image band C) contains a one-to-one mapping from the target FC (multiplied by the local and readout FCs) to the RF FC. To interrogate the spectral response of the sample in the target FC path, one can compare the measured RF band B (or C) with the corresponding sample-free result. Balanced detection can eliminate band A and D since they are common-mode signals, which would double the bandwidth for band B and C. Fig. 1e presents the same principle of CCS in the time domain. The target pulses are sampled by the local pulses, generating SFG pulses, which then interfere with the readout pulses, leading to the RF interferogram. More details can be found in Supplementary Sections 2 and 3.

### Detection efficiency and bandwidth

**Fig. 2:**
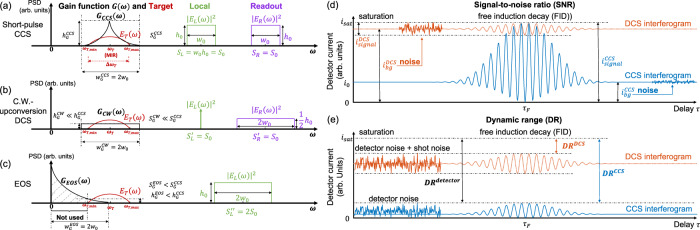
Comparison of detection efficiency, bandwidth, SNR and DR between short-pulse CCS and other dual-comb-based techniques. **a–c** Power gain function *G*(*ω*) for quantification of detection efficiency and bandwidth for three upconversion methods: short-pulse CCS **(a)**, C.W. upconversion DCS **(b)**, and EOS **(c)**. The spectral amplitude of the target (*E*_*T*_ (*ω*)) and spectral intensities of the local (${\left|{E}_{L}\left(\omega \right)\right|}^{2}$) and readout (${\left|{E}_{R}\left(\omega \right)\right|}^{2}$) FCs are denoted by curves in red, green, and purple, respectively. The instrument response function *H*(*ω*) is the convolution of ${E}_{L}^{*}\left(-\omega \right)$ and *E*_*R*_ (*ω*), and its spectral intensity ($G\left(\omega \right)={\left|H\left(\omega \right)\right|}^{2}$) is denoted by the black curve for each method. **a** Short-pulse CCS. *h*_0_, *w*_0_ and *S*_*L*(*R*)_ denote the height (PSD), width (bandwidth), and area (total average power) of the local (readout) spectrum, respectively. Δ*ω*_*T*_, *ω*_*T,min*_, *ω*_*T*_, *ω*_*T,max*_ denote the bandwidth and minimum, center, and maximum optical frequency of the target spectrum to be detected, respectively. *w*_*G*_, *h*_*G*_ and *S*_*G*_ denote the width, maxima, and total area of (under) *G*(*ω*), respectively. **b** C.W. upconversion DCS. **c** EOS. The grey-dashed area of *G*_*EOS*_ (*ω*) denotes the part not effectively used in detection since it does not overlap with the target spectrum. **d**–**e** Comparison between DCS (red curves) and CCS (blue curves) interferograms at FID. **d** SNR comparison. Here we assume enough optical power for both techniques to saturate the detector for highest SNR. In DCS, the weak FID must be accompanied by the strong background from the excitation pulse center, which only contributes to noise here. Contrarily, CCS is free from such a background and can get an interference pattern of higher visibility with smaller noise. *i*_*signal*_, the range of the beating signal. *i*_*bg*_, the background current; *i*_*sat*_, the saturation level of the detector. **e** DR comparison. Here we assume enough optical power for both techniques to detect an identical level of weak absorption. In DCS, a large part of the detector DR is occupied by the background. The higher the sensitivity (lower detection limit) reached, the larger the background, and the smaller the remaining DR. However, in principle, CCS does not have such problem and can make use of a larger part of the detector DR.

Fig. 2a–c compares CCS with the other two upconversion methods in terms of detection efficiency and bandwidth. To quantify their performance, we define a power gain function *G*(*ω*)=|*H*(*ω*)|^2^, where $H(\omega )={E}_{L}^{\ast }(-\omega )\otimes {E}_{R}(\omega )$ is the instrument response function, in which *E*_*L*_ (*ω*) and *E*_*R*_ (*ω*) denote spectral envelopes (Fourier transforms of a single pulse) of the local FC and readout FC, respectively. The width *w*_*G*_, maximum value *h*_*G*_ and total area *S*_*G*_ of *G*(*ω*) can quantify the bandwidth, highest gain, and total gain of the detection, respectively. For short-pulse CCS (panel (a)), we set the spectra of the local FC and readout FC to be both rectangular function with the same height, width, and area. Their corresponding *G*_*CCS*_ (*ω*) (black curve) has a width equal to the sum of their bandwidths, which is required to be larger that of the target spectrum *E*_*T*_ (*ω*) (red curve), i.e., Δ*ω*_*T*_, to detect the whole target band. As for the C.W. upconversion DCS (panel (b)), if we keep the average power of the local FC identical to that of the short-pulse CCS, the local spectrum can be approximated by a Dirac-delta function as it has a finite area, a near-zero bandwidth and a near-infinite spectral density. For a fair comparison, we keep the total average power and the total optical bandwidth of the local FC and readout FC the same as for CCS. To this end, the bandwidth of the readout FC is doubled, and its spectral intensity is halved. As shown in Fig. 2b, although *G*_CW_ (*ω*) has the same bandwidth as G_CCS_ (ω), its ${h}_{G}^{{CW}}$ and ${S}_{G}^{{CW}}$ are both much smaller than those of CCS by a factor proportional to the peak power ratio between a short pulse and C.W. laser of the same average power. This detection gain advantage of CCS is rooted in the much higher peak power that (femto-second) short pulses can offer for the nonlinear wavelength-conversion process, compared to C.W. lasers.

EOS (Fig. 2c) is slightly different as it uses only one “local FC” to play the role of both the local FC and readout FC of the prior methods (see also Fig. 4 and Supplementary Section 3). Again, to keep the total bandwidth and total power the same as those of short-pulse CCS for a fair comparison, we double the bandwidth of the *E*_*L*_ (*ω*) here and keep its height unchanged. Note that the *H*(*ω*) in this case is the autoconvolution of the *E*_*L*_ (*ω*). As shown in Fig. 2c, although the nominal bandwidth of *G*_*EOS*_ (*ω*) is the same as the others, most of it is not effectively utilized (grey-dashed shadow) as it does not overlap with the target spectrum (*E*_*T*_ (*ω*)). To cover the whole *E*_*T*_ (*ω*), the local bandwidth is required to be larger than *ω*_*T,max*_ instead of just Δ*ω*_*T*_ (as is the case for the other two methods), which causes experimental challenges and is an ineffective use of bandwidth. For example, to detect a 30-THz MIR band from 60 THz to 90 THz (5–3.33 µm), EOS would require very short local pulses with a bandwidth of at least 90 THz, i.e., 155–245 THz (1.22–1.93 µm) if it is centered around 200 THz (1.5 µm). Meanwhile, CCS only needs a total bandwidth of 30 THz. Moreover, since only the part of *G*_*EOS*_ (*ω*) that overlaps with *E*_*T*_ (ω) contributes to the detection, its effective *h*_*G*_ and *S*_*G*_ are much smaller than their nominal values and depend on the values of *ω*_*T,min*_ and *ω*_*T,max*_. Using the parameters from the previous example, *h*_*G*_ and *S*_*G*_ of EOS are calculated to be 4/9 and 4/27 those of CCS, respectively, even though EOS can be more experimentally demanding. Additional details regarding this section can be found in Supplementary Sections 3 and 4.1.

### Wavelength conversion and temporal gating

The optical nonlinearity can provide CCS with advantages over DCS in two ways. The first is wavelength conversion. Generally, photodetection in the NIR has a better performance, a lower cost, and does not require cooling. Also, typical commercial MIR detectors have a cut-off wavelength around 13 µm and are limited for the detection of longer wavelengths. Converting MIR information to the NIR can get around these limitations.

Second, and more importantly, strong nonlinearity from short pulses gives rise to a temporal gating effect^11^ that can endow CCS with better SNR, sensitivity, and dynamic range compared to DCS. To demonstrate such advantages, we compare CCS and DCS directly at the free induction decay (FID) part of their interferograms. The FID is the sample’s reradiation which contains molecular signatures^29–31^, and isolating it from the center-burst has been demonstrated to provide the absorption spectrum with a better detection performance^11^. Therefore, the SNR and dynamic range of the two techniques at the FID can be a good indicator of their sensing capability, the comparisons of which are illustrated in Fig. 2d, e, based on our simplified theoretical model (Supplementary Sections 4.2). Note that, in this paper, “FID” refers to either the tail in the electric field of the target pulse (relative to the “pulse center”, see “Target” in Fig. 1e) or its corresponding part of the interferogram (relative to the “center-burst”, see “Interferogram” in Fig. 1e). In DCS (red dotted curve in Fig. 2d), the weak FID beating must be on top of a strong background (the “direct-current” baseline), most of which comes from the power of the strong center of the excitation pulse which only contributes to the noise (shot noise and relative intensity noise) here but not the signal (interference). This kind of baseline is referred to as background signal (power), and this kind of noise is referred as “background noise” in the following text. On the contrary, the FID beating in CCS is free from extra background (blue curve in Fig. 2d), because the FID part of the target pulse is temporally isolated from the strong pulse center by the short local pulses (see Fig. 1e). In other words, it is possible to get an “ideal” interference pattern with full interferometric visibility (SNR) at the FID in CCS, while that of the DCS is greatly limited by the background noise from the excitation pulse center; the lower the absorption to detect, the higher the excitation power (background) that must be used, and the larger this difference can be. Note that saturation at the center-burst part of the interferogram is ignored for both cases.

The SNR comparison naturally leads to a comparison of sensitivity, i.e., the minimum detectable absorption. In DCS, higher optical power gives higher SNR and sensitivity, which are ultimately limited by the detection saturation or relative intensity noise (RIN) of the sources^12^. However, as CCS detects the FID in a “background-free” manner, its fundamental limitation is the nonlinear upconversion capability. That is, the upconverted weak FID signal just must be stronger than the detector noise (NEP) to be detectable, instead of the shot noise or RIN from the strong background as in DCS. Therefore, CCS is not fundamentally limited by the detector saturation or extra noise from the strong excitation background, which sets a hard boundary for DCS.

Fig. 2e illustrates the dynamic range comparison. In CCS, as mentioned before, the detector noise limits the weak side of the absorption signal, and the FID detection can utilize the full DR of the detector. Meanwhile, the background in DCS occupies a large part of the detector DR, which inevitably decreases the room for the FID. In fact, a weaker absorption would require a higher excitation (background) to provide sufficient SNR for detection, which occupies more of the detector DR, further decreasing the DR of the FID (absorption). However, this “sensitivity-DR tradeoff” does not exist in CCS as the excitation background is excluded in the FID detection. In principle, CCS can take advantage of the full DR of the detector. It should be noted that, although here we assume an FID signal that is clearly separated from the pulse center for clarity of our theoretical model, our arguments hold for general cases and do not rely on special temporal features of the FID that could be unique to some molecules. See Supplementary Section 5 for a more detailed discussion.

Among the three upconversion methods, C.W. upconversion DCS can only have the advantage of wavelength conversion, since the C.W. laser cannot provide any temporal gating and it is still basically a DCS detection. As for EOS, one may think it can have all discussed advantages since it uses even shorter pulses for upconversion than CCS. However, this is not fully true since its “local pulse and readout pulse” come from the same pulse; therefore, some additional efforts may be required to independently tune their power and spectra^25,26^ so that, for a very weak FID, the readout power will not saturate the detector before enough local power is applied for upconversion. By contrast, in CCS, as the local pulses and readout pulses are inherently separated, their powers can be independently tuned directly according to the absorption to be detected, and hence the detector saturation can be easily avoided. In short, the flexibility of short-pulse CCS can utilize the full advantages offered by the optical nonlinearity. A detailed discussion for this section can be found in Supplementary Sections 4.2 (theoretical model) and 5 (simulation).

### Setup

To experimentally demonstrate CCS in the MIR, we conduct a measurement of atmospheric carbon dioxide (CO_2_) around 4.25 µm (2349 cm^−1^, antisymmetric stretching mode ν_3_). The target FC consists of 50-fs pulses centered at 4.2 µm with 500 mW of average power provided by two-stage cascaded efficient half-harmonic optical parametric oscillators (OPOs), which are intrinsically phase locked to the pump frequency comb (a mode-locked Yb-fiber laser) at 1 µm^13^. The local FC is a NIR FC centered at 1560 nm (a mode-locked Er-fiber laser) with a 100-nm (400-cm^−1^) FWHM bandwidth, 100-fs pulse width and a 200-mW average power (Menlo Systems FC1500-250-WG). The *f*_*rep*_ of the target FC and local FC are both around 250-MHz and are locked to an RF rubidium (Rb) clock with a shift of 1 kHz. The *f*_*ceo*_ of the Yb-fiber laser and the Er-fiber laser are both locked via standard f-to-2f techniques. The readout FC is a band-pass-filtered part of a supercontinuum generated by the local FC, which is centered around 1145 nm with a 6-nm (45-cm^−1^) FWHM bandwidth and a 2-µW average power. CCS is achieved through SFG of the target FC and the local FC in a 1-mm-long periodically poled lithium niobate (PPLN) crystal followed by its interference with the readout FC, which is measured by a 100-MHz InGaAs balanced detector (Thorlabs PDB415C). The PPLN crystal (Covesion MOPO1-0.5-1) has a 29.52-µm poling period that can provide a ~200-cm^−1^ (~350-nm) quasi-phase-matching 3-dB bandwidth for the SFG. Supplementary Section 1.1 presents the detailed setup and optical spectra of those FCs.

### Experimental results

**Fig. 3:**
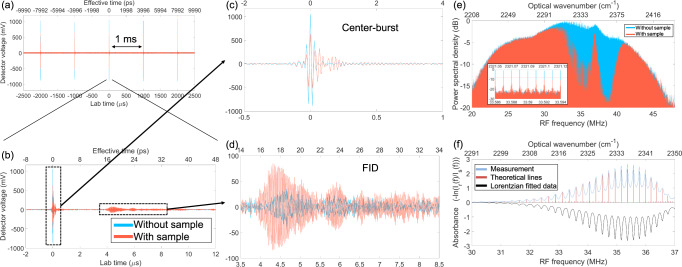
Experimental results of CCS of CO_2_. **a** Five consecutive interferograms with a 1-ms temporal spacing, corresponding to δ = 1 *kHz*. The “without sample” result (blue) is measured when the optical path is purged with nitrogen (N_2_), and the “with sample” measurement (red) is taken when the path is not purged and atmospheric CO_2_ is present. All measurements are carried out at room temperature and atmospheric pressure without extra control. **b** The central 14 µs of one example interferogram. Blowups depicting additional details of the center-burst and FID are shown in panel (**c**) and (**d**), respectively. The lower temporal axes denote the lab time while the upper ones denote the effective time^51^, which are related by the equation ${t}_{{Lab}}/{t}_{{Effective}}={f}_{{rL}}/\delta$. **e** Spectra of band B of the RF FC, obtained by Fourier transforms of 498 consecutive unapodized interferograms, for measurements both with and without CO_2_, are shown in red (*I*_*s*_ (*f*)) and blue (*I*_*r*_ (*f*)), respectively. The inset is a zoomed-in view to show resolved comb lines, which are separated by *δ* = 1000 H*z* in the RF domain corresponding to *f*_*r,L*_ = 250,250,820 Hz in the optical domain. **f** Measured molecular absorbance spectrum (light blue curve), *A*(*f*), defined by $A\left(f\right)=-{{{{{\rm{ln}}}}}}[{I}_{r}(f)/{I}_{s}\left(f\right)]$. The result is obtained from 498 interferograms (for both “with” and “without sample”) each apodized with a 100-µs window. The black curve (inverted) denotes the theoretical model, which is derived by fitting the absorption lines from the HITRAN database (red lines) with a Lorentzian lineshape to the experimental result. The upper axes in both **(e)** and **(f)** denote the optical frequency in wavenumber.

Fig. 3a presents five consecutive interferograms with a period of 1 ms, out of which the central 14 µs of one interferogram is depicted in Fig. 3b. The prominent effects due to CO_2_ can be observed in both the center-burst (Fig. 3c) and the tail (Fig. 3d), which is the result of the coherent addition of molecular FID^29,32^. Note that, thanks to the temporal gating, the background power at the tail (FID) is much weaker than that at the center-burst. This background is not visible in the measurement shown in Fig. 3 as it is concealed by the balanced detector, but it is prominent if the detector is not well balanced (Supplementary Fig. 3). Based on the measurement, we estimate a single-shot time-domain SNR of 167 $\left(\frac{\pm \,1000{mV}}{\pm 6{mV}}\right)$ in a 28-MHz electrical bandwidth, which is more than four times that of a recent EOS work^22^. On the other hand, we estimate an upconversion (SFG) efficiency of at least 2% *mm*^−1^, which is more than two orders of magnitude higher than that of a recent C.W. upconversion DCS work (Supplementary Section 1.3).

Fig. 3e represents the results in the frequency domain, obtained by the Fourier transform of 498 consecutive interferograms (498 ms) without apodization for both the “without sample” and “with sample” cases, where ~2.78 × 10^4^ comb teeth are present in a 245-cm^−1^ band. The average SNR of the without-sample spectrum is 28.9, which gives a sum of spectral SNR of 8.03 × 10^5^ (the sum of the SNR of all comb teeth). Note that we are only able to acquire 0.5-s data with *δ* = 1 kHz, which means the signal spectrum only uses 28 MHz of the whole 125-MHz Nyquist band (half *f*_*rL*_), limited by the memory depth of our data acquisition equipment. If we use a factor of 125/28 larger *δ* and acquire for 1 s, we can get ~ 9 times as many interferograms, which will scale up the sum of spectral SNR by a factor of 3. This leads to an estimated figure of merit of 2.4 × 10^6^ Hz^1/2^ for this MIR spectrometer (Supplementary Section 1.3), which is one of the highest among recently reported MIR DCS or EOS works^4,5,7,22,27^. Note that our SNR can still be further improved since the time-domain signal only reaches about half of the detector saturation (± 1 V out of ± 1.8 V), which can be increased through a higher power from any of the target, local or readout FC.

Shown in Fig. 3f (light blue curve), the molecular absorbance spectrum is obtained by comparing with-sample and without-sample measurements (here a 100-µs apodization window is applied to the interferograms before Fourier transform). Only the *P* branch (rotational structure below the band origin) of the measured spectrum of CO_2_ is shown here (see Supplementary Section 1.4). The theoretical absorbance spectrum (black curve, inverted about the x-axis for clarity) is calculated using spectral lines (red lines) from the HITRAN database^33^ fitted with a Lorentzian line shape of 0.8-cm^−1^ FWHM linewidth.

Note that these results are obtained by locking the *f*_*rep*_ of the target comb and local comb only individually to a RF standard, which gives fixed *f*_*rep*_ values but an uncontrolled broad relative linewidth between two combs^34^. By some post-processing (without external optical referencing), we can correct the without-sample measurements but are unable to fully correct the with-sample measurements (Supplementary Section 1.4). We believe this is the main reason why the fitted absorption linewidth (0.8 cm^−1^) is larger than the theoretical pressure broadening (~0.2 cm^−1^) at room temperature and atmospheric pressure. Similar to other DCS techniques, this problem can be solved by utilizing an intermediate C.W. reference to provide fast phase noise information for either tight-locking using fast actuators^4–7,29,35^ or error correction by post-processing^5,34,36–38^.

## Discussion

**Fig. 4:**
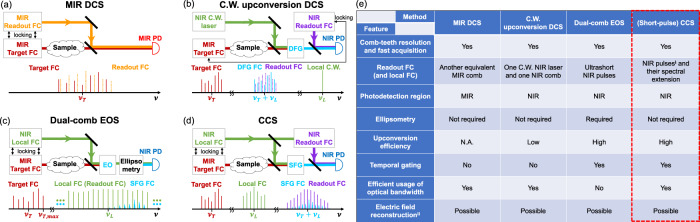
Comparison of principles and features of different dual-comb-based techniques for MIR spectroscopy. **a**–**d** Simplified schematics of different techniques. **a** General dual-comb spectroscopy with an asymmetric (dispersive) configuration^1^. The second MIR FC, which does not pass through the sample, is often referred to as the “local FC” or “slave FC” in other works. However, in the context of this work, it is named the “MIR readout FC” since it samples the MIR target FC linearly, by which a linear cross-correlation signal is generated to give the spectral information of the target FC. **b** C.W. upconversion DCS. The MIR target FC is generated by the DFG between the NIR C.W. laser and the “master NIR comb”^27^, which is not shown in this simplified schematic. This method can be considered as a special case of CCS, in which the “local FC” contains only one “comb tooth”. Note that using an SFG or DFG process for the nonlinear upconversion of the MIR target FC does not make a fundamental difference. **c** Dual-comb EOS. It can also be considered as a special case of CCS, in which the local FC is so broadband that it also serves as the readout FC. The lower-frequency part of the local FC can be regarded as an effective “local FC”, while the higher-frequency part can be regarded as an effective “readout FC”, in the context of CCS. **d** General cross-comb spectroscopy. **e** Table comparing features of different techniques. (*i*) In principle, CCS does not require short pulses as the NIR local FC. However, the (short) local pulses enhance the upconversion efficiency and enable temporal gating. (*ii*) If the electric field of the readout FC (and local FC if applicable) is (are) known, all four techniques can fully reconstruct the electric field of the target pulse. However, this extra information is not necessary for the purpose of general absorption spectroscopy.

CCS has many advantages compared to other dual-comb-based techniques despite their similarities. Fig. 4a–d illustrates dual-comb-based spectroscopic techniques in the MIR, including DCS, C.W. upconversion DCS, dual-comb EOS, and CCS. In all four techniques, two combs of slightly detuned repetition rates are employed to replace the mechanical scanning stage used in traditional FTIR techniques. In fact, CCS can be considered the general form of frequency-converted DCS. C.W. upconversion DCS and dual-comb EOS are essentially two special cases of the CCS; the former uses a very narrow-band local “FC” with only one “comb tooth”, and the latter uses a very broadband local FC (very short local pulses) which also functions as the readout FC (Supplementary Section 3). The features of these four techniques are summarized in Fig. 4e. Compared to MIR DCS, CCS is free from requiring a second MIR FC as well as the poor performance of MIR detectors, and it can have enhanced performance from temporal gating. Compared to C.W. upconversion DCS, CCS features much higher upconversion efficiency and temporal gating, thanks to the short local pulses. Compared to EOS, CCS does not require ultrashort sampling pulses and ellipsometry to detect polarization rotation, which can be experimentally challenging. CCS can also mitigate the limitation from detection saturation in a more direct way, so it can be more resource-efficient and flexible. Moreover, using an independent readout FC can potentially bring more phase information and favorable signal scaling for strong attenuation^39^.

The SNR and sensitivity of CCS are limited by the upconversion efficiency, instead of the detector and background noise (shot noise or RIN at FID from the power of strong excitation pulse center). There are three factors in the upconversion process: the target pulse (generally MIR), the local pulse (generally NIR), and the nonlinear platform (generally a bulk crystal). The improvement of any of these elements can be exploited to better the performance and capability of CCS. First, in the past decade, there has been impressive progress in the generation of high-power (>100 mW) MIR FCs^13–19^. However, MIR DCS has not taken full advantage of this progress yet, as MIR detectors typically saturate at ~ 1 mW. Admittedly, one can always apply additional detectors and bandpass filters to do parallel and sequential detection^12^ to alleviate this problem, but they add to the system complexity and cost, and still do not fundamentally address the limitation from the strong background noise at the FID due to the nature of linear interference. Conversely, CCS can reap the full benefits of more powerful target pulses as saturation is not a main problem. Although the target pulse used in our experiment is already among the FCs with the highest power in its wavelength region, it can still be improved^13^ and enhance the CCS performance.

Second, for the local FC, we use 1.56-µm local pulses with only a 200-mW average power and 100-fs pulse width, while near-IR combs with orders of magnitude better metrics are available, which can directly benefit the demonstrated CCS. Third, a 1-mm commercial bulk PPLN crystal with a poling period of 29.52 µm is used in our experiment as the nonlinear medium, which provides an upconverted MIR bandwidth of 4080 nm – 4530 nm and limits the upconversion efficiency and bandwidth. The total 9 different poling periods of the crystal are expected to provide a combined SFG bandwidth of 2400 nm to 5200 nm, which can cover the whole bandwidth of our MIR target FC^13^ (3500 nm to 5200 nm, see Supplementary Fig. 2). Recently, developments in lithium niobate nanophotonics have enabled dispersion-engineered waveguides with unprecedented phase-matching bandwidths and nonlinear efficiencies orders of magnitude higher than bulk PPLN crystals^40,41^, which can improve CCS performance. One can also envision including several poling periods or a chirped grating in such waveguides to provide a broad SFG bandwidth without sacrificing the efficiency.

Although the power of the local FC and readout FC used in our experiment are relatively low, the NIR detector is already half saturated at the center-burst. The detector can be fully saturated by simply doubling the power of local FC or readout FC. By combining some or all those above-mentioned state-of-the-art techniques, we expect the FID signal from trace molecules can get close to the saturation limit of the NIR detector so that CCS can provide record-high SNR mode-resolved spectroscopy for record-low concentration samples. This is expected to break the limits of current dual-comb-based techniques and is highly desirable for applications where broadband trace molecular detection plays a significant role, such as breath analysis.

**Fig. 5:**
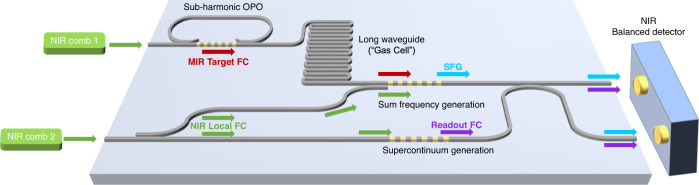
Envisaged on-chip implementation of CCS. Two NIR comb sources of different repetition rates, which are also possible to integrate, are used to pump a single nanophotonic chip. One of the NIR combs is used to pump an on-chip sub-harmonic OPO for the MIR target FC generation, which may then interact with the sample in a long waveguide. The other NIR comb is split into two parts, which are used to function as the local FC via SFG and generate the readout FC via supercontinuum generation in separate poled regions. The outputs are interfered on chip and measured with a NIR balanced detector, which may also be brought on chip.

Currently, our setup relies on free-space and fiber-based components. However, rapid progress in lithium niobate nanophotonics^42,43^ has made it feasible to monolithically integrate most of the components of the entire CCS system into a single photonic chip. In Fig. 5, we show an envisaged on-chip implementation, in which two NIR combs are coupled to the chip, and one of them is used to pump a sub-harmonic OPO to generate the MIR target FC^44,45^. The other NIR comb plays the role of the local FC in sum-frequency generation^44,46,47^, while part of it is used to generate the readout FC via supercontinuum generation^47–50^. A long waveguide is used to increase the interaction area between the target FC and the surrounding environment. Moreover, recent progress in on-chip NIR combs and detectors based on thin-film lithium niobate^42,43^ suggests the potential to also integrate both NIR sources and detectors, which can lead to a fully integrated CCS system.

In summary, we introduce the new concept of cross-comb spectroscopy, which can not only convert spectral information to a more easily accessible wavelength region but also alleviate the limits of the general dual-comb spectroscopy. In a nutshell, CCS can combine and improve upon many of the merits of other demonstrated techniques while circumventing some of their practical challenges. We experimentally demonstrate a CCS measurement around 4 µm with a broad bandwidth, high SNR, and large figure of merit, which are among the best reported for measurements around this wavelength range. This work opens a simple, flexible, and efficient avenue to high-precision, high-sensitivity, high-SNR, high-speed, and broadband spectroscopy in spectral regions with less developed sources and detectors.

## Supplementary information

Supplementary Information

## Data Availability

The data that support the plots within these paper and other findings of this study are available from the corresponding author upon request.

## Code availability

The code that supports cross-comb signal processing and other findings of this study are available from the corresponding author upon request.
