# Supplementary material for: Mid-infrared cross-comb spectroscopy
Source: Nat Commun. 2023 Feb 24;14:1044. doi: 10.1038/s41467-023-36811-7 (PMC9957991; doi:10.1038/s41467-023-36811-7)
Supplement: Supplementary file 1 — Supplementary Information [file 41467_2023_36811_MOESM1_ESM.pdf]

Supplementary Materials for

Mid-Infrared Cross-Comb Spectroscopy

Mingchen Liu[1], Robert M. Gray[1], Luis Costa[1], Charles R. Markus[2], Arkadev Roy[1], Alireza Marandi[1,*]
[1]*Department of Electrical Engineering, California Institute of Technology, Pasadena, California, 91125, USA*
[2]*Division of Chemistry and Chemical Engineering, California Institute of Technology, Pasadena, California, 91125, USA*
[*]*marandi@caltech.edu*

Table of contents

1. Setup and supplementary experimental results 3
1.1 Setup and optical spectra 3
1.2 Temporal gating 4
1.3 Estimation of experimental signal-to-noise ratio (SNR) and figure of merit (FOM) 4
1.4 Phase correction and broadened absorption linewidth 6
2. One-to-one tooth mapping from target FC to RF FC 8
2.1 Target FC and Local FC 8
2.2 SFG FC 10
2.3 Readout FC 10
2.4 RF FC, one-to-one mapping, and absorption spectrum 10
2.5 Universality 11
2.6 Bandwidth requirements for Local FC and Readout FC 12
2.7 Bandwidth requirements for repetition rates and carrier–envelope offset frequency (CEO) frequencies 13
3. Comparison of principle between different techniques 16
3.1 DCS 17
3.2 CCS 18
3.3 C.W. upconversion and EOS 19
3.4 C.W. upconversion and EOS described by comb-teeth mapping 20
4. Comparison of performance between different techniques, by theoretical model 22
4.1 Detection bandwidth and efficiency of upconversion methods 22
4.1.1 General CCS, symmetric CCS, and C.W. upconversion CCS 22
4.1.2 Symmetric CCS and EOS CCS 24

4.1.3 Comparison of General CCS, symmetric CCS, and C.W. upconversion CCS under a different assumption 26
4.1.4 Summary 27
4.2 Comparison between DCS and CCS: temporal gating, sensitivity, SNR, and dynamic range 28
4.2.1 Overview 28
4.2.2 Assumptions of the model 29
4.2.3 SNR at FID, without temporal filtering 31
4.2.4 SNR at FID, with temporal filtering 32
4.2.5 Sensitivity and dynamic range, with temporal filtering 33
4.2.6 SNR scaling with power of different combs 34
4.2.7 Summary 35
5. Comparison of performance between different techniques, by simulation 36
5.1 Simulation assumptions 36
5.1.1 Frequency combs 36
5.1.2 Sample 36
5.1.3 Detectors 36
5.1.4 Noise 37
5.1.5 Nonlinearity 37
5.2 DCS 37
5.3 CCS (without temporal filtering) 39
5.4 CCS (with temporal filtering) 41
5.5 Comparison 43
References 46

1. **Setup and supplementary experimental results**

1.1 Setup and optical spectra

The setup diagram is depicted in Supplementary Fig.1, and the optical spectra are depicted in Supplementary Fig. 2.

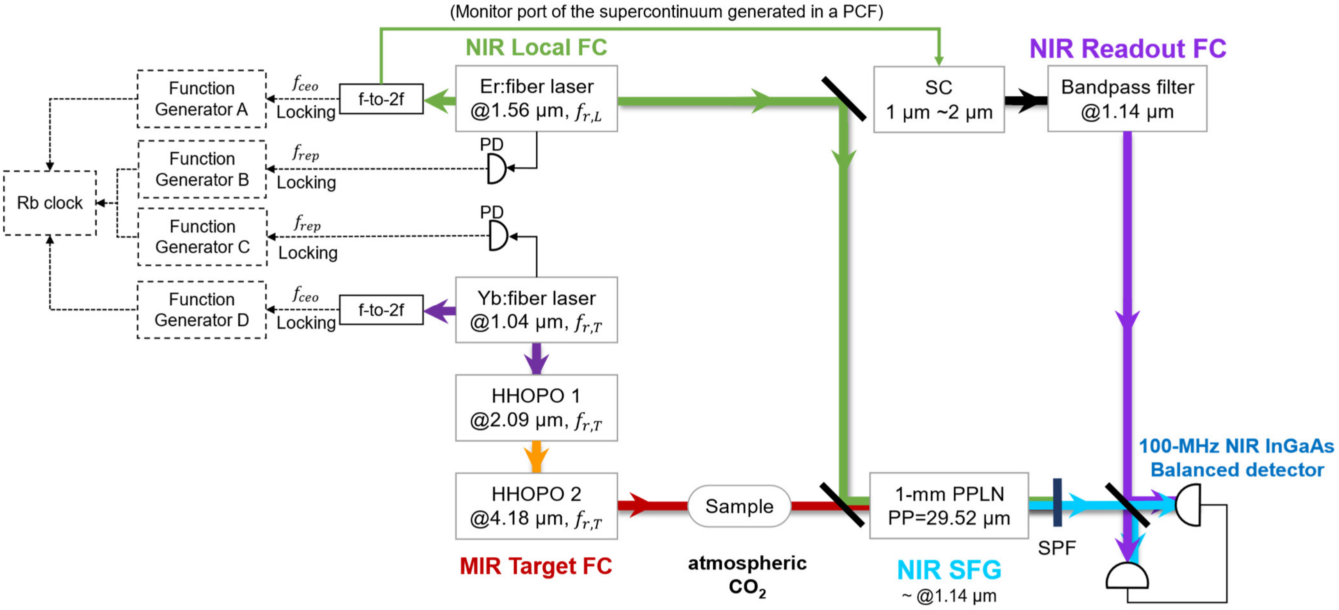

Supplementary Fig. 1| Experimental setup. PCF: photonic crystal fiber. SC: supercontinuum. PD: photodetector. PP: poling period. SPF: shortwave pass filter. The supercontinuum, used as the readout FC after a bandpass filter, is from a monitor port (tap output) of the PCF pumped by the local FC in its f-to-2f module. The SFG signal and readout FC are mixed in a commercial 2x2 50:50 wideband fiber optics coupler (Thorlabs TW1064R5A2A), before which the two beams are coupled from free space into fiber by commercial fiber collimators. The configuration of the fiber coupler is further illustrated in Supplementary Fig. 3a. For the SFG signal, two commercial freespace shortwave pass filters (Spetrogon SP-1300) are used to block residual NIR local power and MIR target power. For the readout FC, the bandpass filter (Delta Optical Thin Film A/S LF104008) for the supercontinuum is centered at 1140nm with a full-width at half-maximum (FWHM) bandwidth of 15 nm. The NIR detector is a commercial InGaAs fiber-coupled balanced detector (Thorlabs PDB415C).

The target FC is provided by a chain of two cascaded half-harmonic OPOs[1]. Pumped by a commercial mode-locked Yb: fiber laser centered at 1.045 μm, the first half-harmonic OPO generates 2.09-μm pulses, which are then used to pump the second half-harmonic OPO at 4.18 μm. Half-harmonic OPOs feature intrinsic phase and frequency locking of their output to the pump[2]; thus the phase and frequency of the 4.18-μm OPO are intrinsically locked to that of the 1.045 μm pump. Hence, by locking the f_{rep} (f_{ceo}) of the 1.045-μm laser to that of the 1.55-μm Er: fiber laser (local FC), the target FC (4.18-μm OPO) is locked to the local FC. In this experiment, the f_{rep} and f_{ceo} of the local FC and 1.045-μm Yb fiber laser (target FC) and all measurement apparatus are locked to a 10-MHz RF rubidium (Rb) clock, ensuring a common frequency standard.

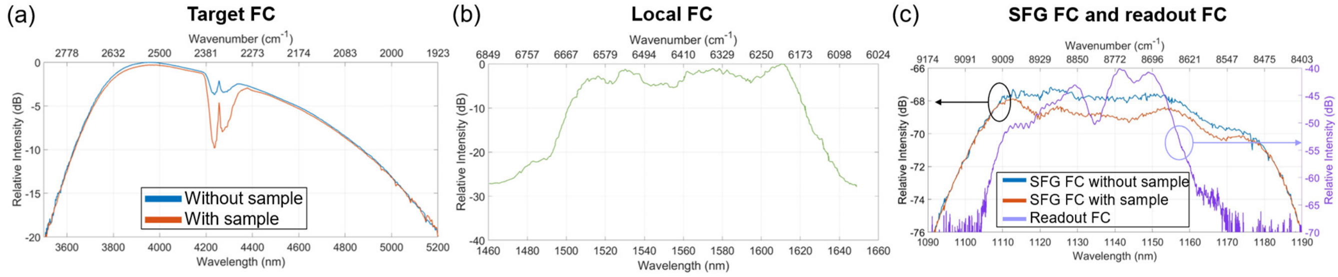

Supplementary Fig. 2| Optical spectra of frequency combs used in the experiment. a. Target FC for both "without sample" (purged) and "with sample" (unpurged) cases, measured by a commercial Fourier-transform infrared spectrometer (FTIR) with a resolution of 4 cm^{-1}. The residual CO_2 which cannot be fully cleared by purging is the reason why the absorption dip can still be observed in the "without sample" curve. **b.** Local FC spectrum provided by the manufacturer (Menlo Systems). **c.** Spectra of SFG FCs (with and without sample) and readout FC measured by a grating-based OSA with a resolution of 0.5 nm.

1.2 Temporal gating

For the same reason as in EOS[3], our cross-comb method can also benefit from the temporal gating (also referred to as "nonlinear gating"), although our local pulse is not as short as that of EOS. However, this effect cannot be seen in the measurement shown in Fig. 3 of the main paper because the balanced detection conceals the strong background. Corresponding to band A and band D of the RF FC (see Section 2.4), the background is a common-mode signal only from the port of the SFG FC and thus is cancelled by the balanced detection. Note that the background at the center-burst is basically an intensity cross-correlation of the target pulses and local pulses, so it is delay (τ, lab-time)-dependent unlike DCS (See Fig. 1 of the main paper). As shown in Supplementary Fig. 3, if we tweak the coupling of the splitter to the balanced detector (panel (a)) such that it is not well-balanced, the strong background will show up prominently at the center-burst (panel (b)). However, because of the temporal gating, the beating at the tail, which contains useful information, is free from any undesirable background power from the strong pulse center. The complete description and importance of temporal gating is more complicated and can be found in Section 3 and 4.2.

Note that the balanced detection can only "conceal" the background in its balanced RF output, but it cannot solve the problem caused by the strong background. Although a well-balanced detector can cancel the common-mode signal and noise in its balanced RF output by comparing the outputs of two photodiodes, there may still be strong optical power incident on each photodiode which is not visible in the balanced output. The strong incident optical power can bring in noise which is not common-mode (e.g., shot noise) and thus cannot be cancelled (indeed they add up), and it will ultimately saturate the photodiodes. This problem exists in detection of weak FID signal for DCS. More illustrations can be found in our simulation (Section 5).

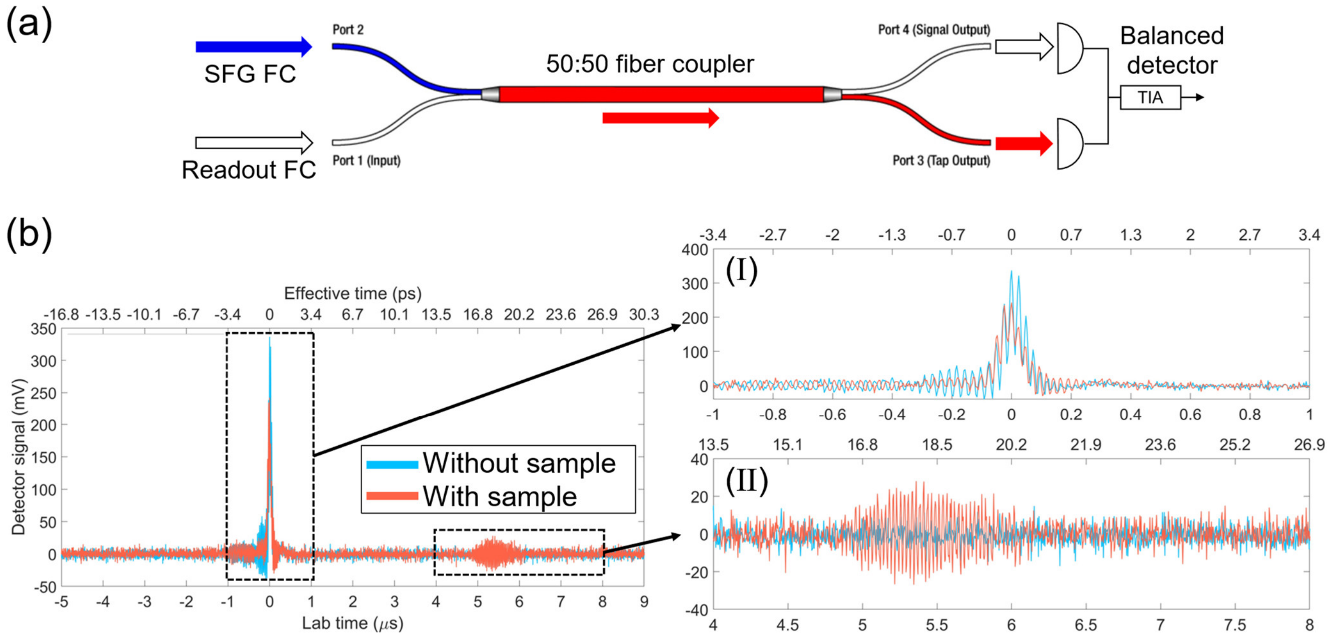

Supplementary Fig. 3| Interferograms of CCS of CO_2, measured by an unbalanced detector. a. Configuration of the fiber coupler and balanced detection. TIA: transimpedance amplifier. **b.** Interferograms measured when the detector is not well balanced. The main figure presents the central 14-μs part of one example interferogram in order to highlight the details of the center-burst (inset I) and the tail (inset II). Note that the measurement is done when the detector is tuned to be just slightly unbalanced. The background at the center-burst is actually very strong and can heavily saturate the TIA if the detector is further unbalanced. Note that the result shown here is from an older measurement where local pulses with lower power are used; thus, its FID signal is lower than that of the Fig. 3 of the main paper.

1.3 Estimation of experimental signal-to-noise ratio (SNR) and figure of merit (FOM)

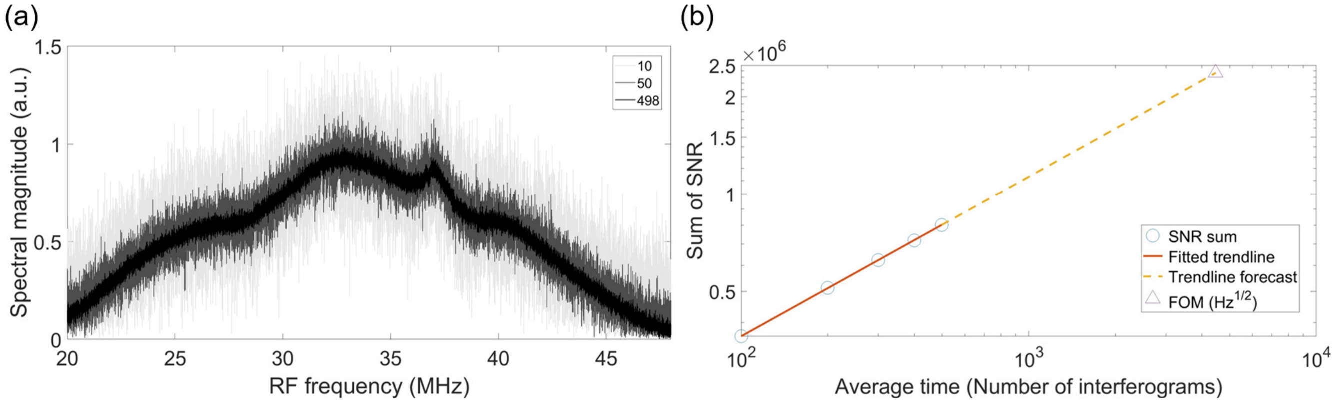

Supplementary Fig. 4| estimation of SNR and FOM. a. averaged without-sample spectra by different numbers of interferograms (10,50,498, denoted by curves with different grayscales). **b.** Sum of SNR as a function of average time (N), where N denotes the number of averaged interferograms. The red line is a linear fitting of the data points (blue circle), and the yellow dashed curve denotes the forecast the trendline, which scales the experimental sum of SNR to an estimation of the FOM (purple triangle). Note that the coordinate is in log-log scale.

Supplementary Fig. 4 presents the estimation of SNR and FOM of our spectrometer. Panel (a) shows averaged without-sample signal spectra by different numbers of interferograms. Note that a small part of the FID signal in the interferograms is discarded before the Fourier transform is applied to exclude the influence of residual sample absorption. It is readily seen that the SNR of the signal spectrum increases with the averaging time. Panel (b) depicts the spectral SNR sum (the sum of the SNRs of all spectral components) as a function of N (number of averaged interferograms) on a log-log scale, with experimental data denoted by the blue circles. A linear fitting (red line) is conducted for those experimental points, whose slope is 0.4936, indicating the SNR increases as a function of $\sqrt{N}$ as expected.

Because of the reasons mentioned in the main paper, currently we are only able to acquire ~0.5-s data with a δ of 1 kHz, which gives a SNR sum of 8.03×10^5 (the highest blue point). To estimate a figure of merit (FOM), we need to scale the number in two ways. Firstly, we assume data acquisition over a full second, which can give twice as many interferograms. Secondly, the signal currently only takes up 28 MHz out of the whole available 125-MHz spectrum (half repetition rate of the local/target FC). If we could set the δ to be ~4.5 kHz (we did not do that in the experiment due to some limitations of our detector and locking electronics), we would be able to get ~4.5 times more interferograms. In total, we can realistically obtain 9 times as many interferograms in our measurement, which leads to an estimation of FOM of 2.4×10^6 (yellow dashed line and purple triangle).

In addition, here we explain how we estimate the upconversion efficiency of our experiment and how it compares to that of prior work using C.W. upconversion[4]. In that work, a 700-μW MIR FC and a 2.4-W C.W. laser are used to get a 1.3-μW upconverted NIR FC with a 20-mm PPLN crystal. Note that all the power in this subsection refers to average power. Therefore, their upconversion efficiency can be calculated as:

$$\frac{P_{upconverted}}{P_{MIR}\times L_{crystal}}=\frac{1.3\ \mu W}{700\ \mu W\times 20\ mm}=9.3*10^{-3}\ \%\ (mm^{-1}) \quad (1)$$

In our experiments, we use 500-mW target pulses and 200-mW local pules to generate a SFG signal of ~100 nW with a 1-mm PPLN crystal. However, in our case, the 100-fs local pulse scans through the 50-fs target pulse due to their different repetition periods (see Fig. 1e of the main paper), so the real time interval in which the two pulses overlap (when SFG power is generated) only accounts a very small portion of the full period. Specifically, when we set the $\delta = 1\ kHz$ for the $f_{rep,L} \cong f_{rep,T} = 250\ MHz$, the local pulse will scan through the target pulse in a total of 2.5×10^5 steps with a step size of approximately 16 fs (meaning the relative position of local and target changes by about 16 fs for each local pulse which samples the target) over the course of an interferogram. Since the target pulse is only around 50 fs, there are only 3~4 steps in which the local pulse overlaps with the target pulse well out of those 2.5×10^5 steps, and moreover, there is only at most one step that

the two pulses overlap perfectly (the maxima of the interferogram). Therefore, we can estimate an effective overlap coefficient (the "duty cycle" of SFG generation) to be about 10^{-5}, which must be factored into the calculation of the SFG efficiency for a fair comparison. Therefore, the efficiency is:

$$\frac{P_{upconverted}}{P_{MIR} \times L_{crystal}} \times \frac{1}{duty\ cycle} = \frac{100\ nW}{500\ mW * 1mm} \times \frac{1}{10^{-5}} = \frac{1}{5 * 10^{6}} \times \frac{1}{10^{-5}} mm^{-1} = 2\%\ (mm^{-1}) \quad (2)$$

Our upconversion efficiency is more than two orders of magnitude higher than the C.W. upconversion work, although the average power of our local FC is just one tenth of theirs. Note that we couple the generated SFG from free space to a single-mode fiber and then measure its power with a fiber-coupled OSA. However, there could be a large loss in the free space-fiber coupling which results in an underestimation of the measured SFG power. Therefore, this efficiency could be correspondingly underestimated.

1.4 Phase correction and broadened absorption linewidth

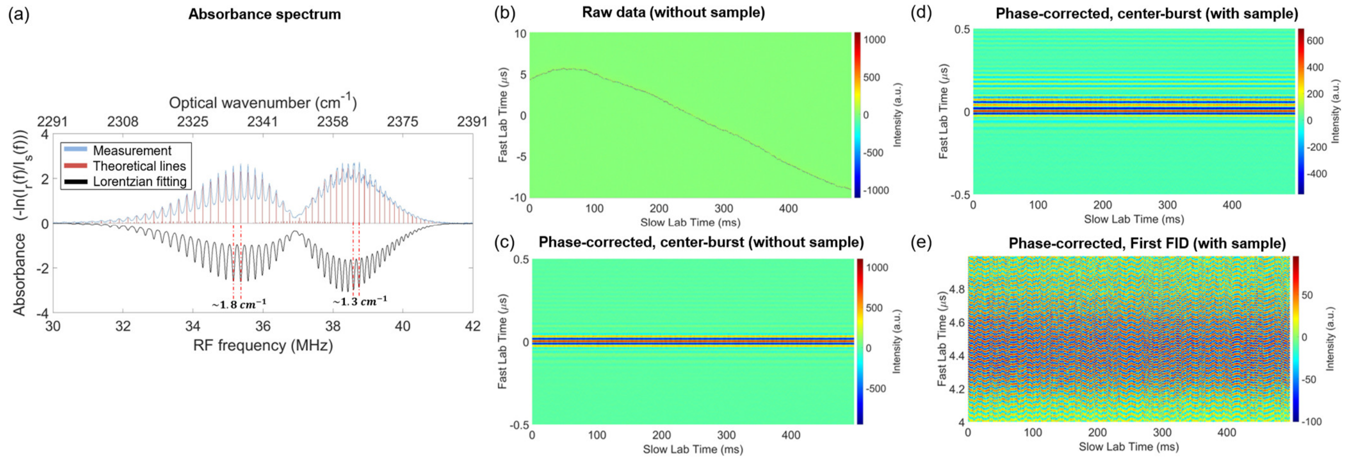

Supplementary Fig. 5|. Full measured absorbance spectrum of atmospheric CO_2 and phase correction process. a. Full measured absorbance spectrum of atmospheric CO_2 in our preliminary cross-comb measurement, including both P and R branches. The SNR of the R branch is lower than that of the P branch. Also, the absorption lines of the R branch are broader than that of the P branch. The spacing between absorption lines in R branch is smaller than that of the P branch. **b.** 3D interferogram of the raw data of the without-sample measurement. **c,** 3D interferogram of the corrected data of the without-sample measurement, around center-burst. **d.** 3D interferogram of the corrected data of the with-sample measurement, around center-burst. **e.** 3D interferogram of the corrected data of the with-sample measurement, around first peak in FID at a fast lab time interval of [4,5] μs.

Supplementary Fig. 5a shows the full measured absorption spectrum, whose right side (higher optical frequency, R branch) has a worse SNR and more broadened absorption linewidth compared to its left-side counterpart (lower optical frequency, P branch). This is because the phase noise (uncontrolled broad relative linewidth) between the target FC and local (readout) FC has a larger effect on the R branch, as explained below.

Panels (b)-(d) show the phase correction for the interferograms, where "3D interferograms" are presented. In those 3D interferograms, each column is a single interferogram (detector voltage is denoted by the colormap), and consecutive single interferograms (columns) are plotted from left to right. Therefore, the vertical axis denotes the "fast time" within each single interferogram, and the horizontal axis denotes "slow time" that shows the time spacing between each interferogram.

Panel (b) shows the without-sample 3D interferogram with the fast time zoomed in to [-10,10] μs to show the center-burst. Ideally, the center-bursts of each single interferogram should perfectly align at $t_{fast} = 0$, but they shift due to the phase noise and timing jitter between the two fiber lasers since no tight locking is applied to them[5]. Two steps are taken to correct these shifts. Firstly, the maxima of the envelopes of each interferogram are shifted and aligned to correct the timing jitter (the envelope of the interferograms is obtained by Hilbert transform). Secondly, a zero-order phase term is applied to each interferogram to make its phase at the maxima of the envelope zero to correct the zero-order phase shift between interferograms.

The obtained results are shown in panel (c)-(e). At the center-burst of both without-sample and with-sample case (panel (c) and (d)), the corrected interferograms overlap very well and thus can be coherently averaged. This is largely because our correction uses the information from the sharp peak structure at the center-burst of the interferograms and thus provides reasonably good correction over the whole center-burst, since it is generated by the interference of two short, femtosecond pulses. This is sufficient for the without-sample measurement since its information only exists around the center-burst. However, for the with-sample case, although its center-bursts are aligned well and can be averaged, prominent phase error still exists at larger fast times, for example, at the first strong FID peak (panel (e)). This is because the coherence time between the two combs is smaller than 4 μs, so the zero-order phase correction at the center-burst is not able to fully correct the error at the more distant FID. The larger the fast time (delay τ), the larger the phase error between different interferograms, and the less they can be averaged. In other words, the relative comb linewidth between our target and local (readout) combs remains broad because their repetition rates are independently locked only to a RF standard.[5]

This explains why our measured absorption linewidth is larger than the theoretical value; although the center-burst of the with-sample measurement can average well, the FID signal at large delay τ cannot. This is like a window function is applied to the time-domain of the averaged with-sample interferogram, which is equivalent to a sinc function convolved to its spectrum which broadens all its spectral features. The R branch of the absorption spectrum suffers more from this undesirable effect because its corresponding time-domain information exists at a larger time delay (fast time) due to a smaller spacing of its absorption lines (see Supplementary Fig.5 (a)), which means a larger phase error and less averaging. More detailed analysis about temporal and spectral features of the CO_2 absorption can be found in ref [6]. As mentioned in the main text, this issue can be solved by setting one intermediate C.W. reference to provide information for either tight-locking by fast actuators, or error correction by data processing, as has been well demonstrated in dual-comb spectroscopy.

However, this effect does not influence the without-sample measurements since their information only exists close around the center-burst in the time-domain where phase error and timing jitter can be corrected based on the information from the sharp peak structure of the center-burst itself. Therefore, in terms of SNR, the obtained without-sample measurements after our phase correction are comparable to the results that can be obtained if the relative comb linewidth (phase noise and timing jitter) between target and local combs are ideally controlled. Hence, our estimate of the FOM of our spectrometer using the SNR of the without-sample measurements is fair.

2. **One-to-one tooth mapping from target FC to RF FC**

2.1 Target FC and Local FC

The electric field of the local FC can be described by

$$e_L(t) = \sum_m A_m^L \exp(i\phi_m^L) \exp(-i2\pi\nu_m t) = \sum_m L_m \exp(-i2\pi\nu_m t) \tag{3}$$

where L_m denotes the complex amplitude that encodes both the intensity and phase of the mth local comb tooth with optical frequency ν_m, and the spatial dependence is omitted here. The superscript "L" of A_m^L and ϕ_m^L denotes local FC, and the subscript "m" corresponds to the mth comb tooth. In addition, for the optical frequency ν_m, we have

$$\nu_m = mf_{r,L} + f_{ceo,L} \tag{4}$$

where $f_{r,L}$ and $f_{ceo,L}$ are the repetition rate and carrier-envelope offset (CEO) frequency of the local FC, respectively.

Sometimes it is not convenient to directly use "m" to index comb teeth, since the first tooth usually occurs at very large m. To be specific, for the first tooth of a practical frequency comb, $m_{first} \sim 10^6$. For convenience, here we define the effective tooth index, m', which starts at 1. If we use m_{first} to denote the tooth index of the first local tooth, we have:

$$\nu_{m'=1} = \nu_{m_{first}} = (m_{first} - 1)f_{r,L} + 1 \times f_{r,L} + f_{ceo,L} = 1 \times f_{r,L} + f_{ceo,L} + \nu_{start,L} \tag{5}$$

Where $\nu_{startL} = (m_{first} - 1)f_{rL}$.

Then, for any $m'^{\,th}$ teeth (m' starts from 1),

$$\nu_{m'} = m'f_{r,L} + f_{ceo,L} + \nu_{start,L} \tag{6}$$

Like the local FC, we use X_n to denote the complex amplitude of the nth target comb tooth with the optical frequency ν_n. We have

$$e_T(t) = \sum_n X_n \exp(-i2\pi\nu_n t), \nu_n = n(f_{r,L} + \delta) + f_{ceo,T} \tag{7}$$

Where δ denotes the repetition rate detuning between the local and target FC, i.e., $f_{r,T} = f_{r,L} + \delta$.

Similarly, we can also define the effective tooth index for target comb teeth,

$$\nu_{n'} = n'f_{r,T} + f_{ceo,T} + \nu_{start,T}, \quad \nu_{start,T} = (n_{first} - 1)f_{r,T} \tag{8}$$

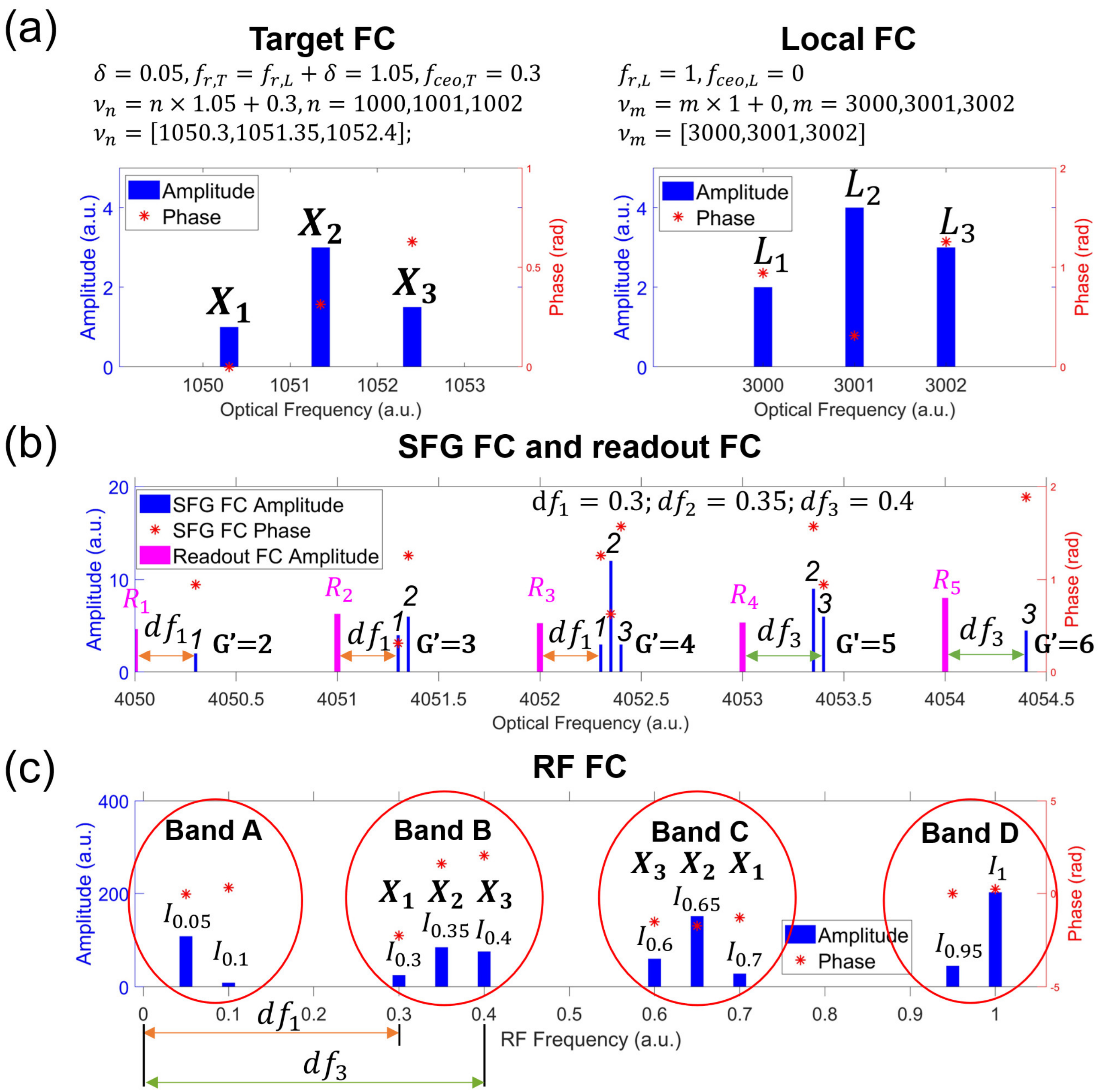

Supplementary Fig. 6| Quantitative illustration of one-to-one tooth mapping of cross-comb spectroscopy. a. Target FC and local FC. Effective tooth indices are used to label each comb tooth in the plot. **b.** SFG FC and readout FC. Each SFG tooth is labeled by the effective tooth index ("1", "2", or "3") of the corresponding target tooth. The phase for the readout FC is assumed to be constant for each tooth and is thus not shown in the plot. $df_{n'}$ denotes the primary readout frequency for the n'^{th} (effective tooth index) target tooth. Each SFG group is labeled by its effective group index G'. **c.** RF FC. Every RF comb tooth in band B and band C is labeled with its corresponding target tooth.

Using the notation introduced above, the frequency-domain picture of cross-comb spectroscopy is depicted in Supplementary Fig. 6. To make a concise and clear illustration, only three teeth are included for both FC, and simple random numbers are assigned to their optical frequencies, which are of arbitrary unit (Supplementary Fig. 6a). Note that generality is not lost by assigning $f_{ceo,L} = 0$, since in practice it is just the relative f_{ceo} between the two FCs that matters. Although only a small number of comb teeth and simple random numbers are used for the following illustrations and equations, the conclusions still hold when scaled to practical numbers.

2.2 SFG FC

Because of the slightly detuned repetition rates between the local and target FCs, each pair of teeth from them will generate an SFG tooth at a unique frequency, the set of which are referred to as the SFG FC. The electrical field of a certain SFG tooth can be described by (phase-matching effect is not included here)

$$E_{n,m}^{sfg} = L_m X_n \exp\left(-i2\pi\nu_{n,m}^{sfg} t\right), \nu_{n,m}^{sfg} = \nu_n + \nu_m \tag{9}$$

As shown in Supplementary Fig. 6b, the resultant SFG comb teeth cluster into different frequency groups[7], which can be indexed by the group index $G = n + m$ (or effective group index $G' = n' + m'$), and the groups are evenly spaced by $f_{r,L} = 1$. The group G' is generated by the SFG between the $\ldots(n'-1)^{th}, n'^{th}, (n'+1)^{th} \ldots$ target teeth and the $\ldots(m'+1)^{th}, m'^{th}, (m'-1)^{th} \ldots$ local teeth. Note that the center group, with $G' = 4$, contains information about all the target teeth, in spite of the fact that different target teeth are modulated by different local teeth (see also Fig. 1 of the main paper). Such a group that contains the information for all target teeth is called a "complete (SFG) group" in the following context. It is readily seen that the number of complete groups formed is determined by the number of local teeth relative to target teeth.

More patterns can be observed within SFG groups. Firstly, SFG teeth in a single group are separated by δ. Secondly, mixing with different local teeth, a given target tooth will generate multiple SFG teeth, which are all at the same relative frequency position in their respective SFG groups. To illustrate the second pattern, each SFG tooth in Supplementary Fig. 6b is labeled by its corresponding target tooth ("1", "2", or "3"). The pattern is made clearer still if readout teeth are introduced as frequency references (see next subsection). These patterns make it possible to do one-to-one mapping between the MIR and RF domains.

2.3 Readout FC

To read out the spectral information of the target FC contained in the SFG FC, another comb, referred to as the readout FC, is employed to beat with the SFG FC on a square-law photodetector. The readout FC is effectively a spectral extension of the local FC and therefore inherits its f_{rep} and f_{ceo}. As shown in the Supplementary Fig. 6b, readout comb teeth can be regarded as "boundary markers" for SFG groups, since they share the same constant distance $f_{r,L}$ between each unit. For a certain SFG group, we name its closest (second closest) readout tooth as its "primary (secondary) readout tooth". For a certain SFG tooth within a SFG group, we name the frequency difference between the tooth and its primary (secondary) readout tooth as its "primary (secondary) readout frequency", and the sum of its primary and secondary readout frequencies is $f_{r,L}$. As shown in the illustration, the SFG teeth generated by the same target tooth always have the same primary readout frequency, even though they are distributed in different SFG groups and correspond to different primary readout teeth. Also, SFG teeth generated by different target teeth have different primary readout frequencies, denoted by $df_{n'}$ in the illustration. These two patterns are very important and provide the foundations for the one-to-one mapping.

As with the local and target FCs, we use "R_q" to denote the complex amplitude of the qth comb tooth of the readout FC.

$$e_R(t) = \sum_q R_q \exp\left(-i2\pi\nu_q t\right), \nu_q = q f_{r,R} + f_{ceo,R} \tag{10}$$

Also, we can define the effective tooth index for readout comb teeth:

$$\nu_{q'} = q' f_{r,R} + f_{ceo,R} + \nu_{start,R}, \ \ \nu_{start,R} = \left(R_{first} - 1\right) f_{r,R} \tag{11}$$

Note that $f_{ceo,R} = f_{ceo,L}$ and $f_{r,R} = f_{r,L}$.

2.4 RF FC, one-to-one mapping, and absorption spectrum

Based on the SFG and readout comb teeth in the optical domain, one can calculate the resultant RF spectrum detected by a single square-law detector. The bandwidth of the detector is assumed to be "1" ($f_{r,L}$), which means that the highest RF frequency the detector can detect is the repetition rate of the local FC, $f_{r,L}$. This is a common condition for many works in dual-comb spectroscopy. To calculate the RF signal (photocurrent) at a given RF frequency, one must sum the contributions from all the comb tooth pairs that can generate heterodyne beating at this frequency.

$$I_{f_0} = \sum_{f^{rf}=f_0} A_1 A_2^* , f^{rf} = \nu_1 - \nu_2 = f_0 \tag{12}$$

A_1 and A_2 denote the complex amplitude of the two involved comb teeth, which can be from the SFG or readout FC. The RF frequency of the beating signal, f_{rf}, is equal to the difference between the optical frequencies of the two involved comb teeth. Following these equations, for the case of this illustration, the RF signal at different frequencies can be calculated

$$Band\ A \begin{cases} I_{0.05} = (L_1 L_2^* + L_2 L_3^*)(X_1^* X_2 + X_2^* X_3) \\ I_{0.1} = (L_1 L_3^*) X_1^* X_3 \end{cases} \tag{13}$$

$$Band\ B \begin{cases} I_{0.3} = (L_1 R_1^* + L_2 R_2^* + L_3 R_3^*) X_1 \\ I_{0.35} = (L_1 R_2^* + L_2 R_3^* + L_3 R_4^*) X_2 \\ I_{0.4} = (L_1 R_3^* + L_2 R_4^* + L_3 R_5^*) X_3 \end{cases} \tag{14}$$

$$Band\ C \begin{cases} I_{0.6} = (R_4 S_1^* + R_5 S_2^* + R_6 S_3^*) X_3^* \\ I_{0.65} = (R_3 S_1^* + R_4 S_2^* + R_5 S_3^*) X_2^* \\ I_{0.7} = (R_2 S_1^* + R_3 S_2^* + R_4 S_3^*) X_1^* \end{cases} \tag{15}$$

$$Band\ D \begin{cases} I_{0.95} = L_3 L_1^* (X_1 X_2^* + X_2 X_3^*) \\ I_1 = (L_2 L_1^* + L_3 L_2^*)(X_1^* X_1 + X_2^* X_2 + X_3^* X_3) + (\sum_{q=1}^{4} R_q^* R_{q+1}) \end{cases} \tag{16}$$

Note that, for simplicity, here we use subscript effective tooth indices "1,2,3", "1,2,3" and "1,2,3,4,5,6" (m', n', q') to index different comb teeth of the target FC, local FC and readout FC, respectively. The resulting teeth are also illustrated in Supplementary Fig. 6c and are referred to as the "RF FC".

As shown in the illustration, RF FC comb teeth can be classified into four bands[8]. Band A consists of the intra-group beat notes, which are generated by two SFG teeth from the same SFG group. Band D is also composed of beat notes generated by two SFG teeth, but the two teeth are from two different adjacent SFG groups. Note that the frequency component with $f^{rf} = f_{rL} = 1$ is a special component in band D which also includes the contribution from beatings between two readout teeth. Band A and band D result from only the SFG FC (excluding $f_{rf} = 1$) and correspond to the envelope of the SFG pulses (cross-correlation signal between target and local FC) in the time domain, which doesn't contain much useful information for our purpose. In contrast, band B, consisting of beat notes between SFG teeth and their primary readout teeth, is a one-to-one mapping of the original target FC. As demonstrated in the equations, the complex amplitude of a certain band B RF tooth is related to and directly proportional to that of only one target tooth, although it is generally modulated by more than one local tooth and readout tooth. Like band B, band C is also a one-to-one mapping of the original target FC, resulting from beating between SFG teeth and their secondary readout teeth. Band B and C contain the exact same information regarding the target FC, which are mirror images of each other, reflected about $f_{r,L}/2$ in the RF domain.

Based on the one-to-one mapping, the absorption spectrum in the MIR region interrogated by the target FC, including both amplitude and phase, can be obtained by comparing the RF band B (C) measured with the sample in the path and the corresponding result measured without the sample in the path (reference).

2.5 Universality

In our experiment, we use a MIR synchronously pumped degenerate OPO (centered at 4.18 μm) and Er-doped fiber laser (centered at 1.55 μm) as the target FC and local FC, respectively. The readout FC is a band-pass filtered portion of a supercontinuum pumped by the local FC, which is generated in a photonic crystal fiber (PCF). It should be noted that the scheme of cross-comb spectroscopy (CCS) doesn't have any limitation on the laser techniques used for the frequency comb generation. However, the current implementation benefits from the intrinsic phase locking of the mid-IR comb to the Yb: fiber

laser pump. Also, as a special case of CCS, the local FC or readout FC can be replaced by a "frequency comb" with only one tooth, i.e., a C.W. laser. This is explained in depth in the following section.

Moreover, in this derivation, we demonstrate the frequency-up-conversion one-to-one comb tooth mapping by SFG. In fact, it is also possible to realize one-to-one mapping by difference frequency generation (DFG), the derivation of which is very similar. This may be useful in the application of frequency-comb-based spectroscopy in the ultraviolet spectral range or for even shorter wavelengths.

2.6 Bandwidth requirements for Local FC and Readout FC

To realize one-to-one mapping for all teeth of the target FC, local FC and readout FC, one must satisfy some requirements which will be discussed in detail in this subsection. To provide a concise discussion, we continue to use the simple illustration above, keeping the number of target teeth to be three but varying the number of local teeth to be 2, 3, or 4. The results are shown in the Supplementary Fig. 7. N, M, and Q denotes the number of teeth of the target, local and readout FCs, respectively.

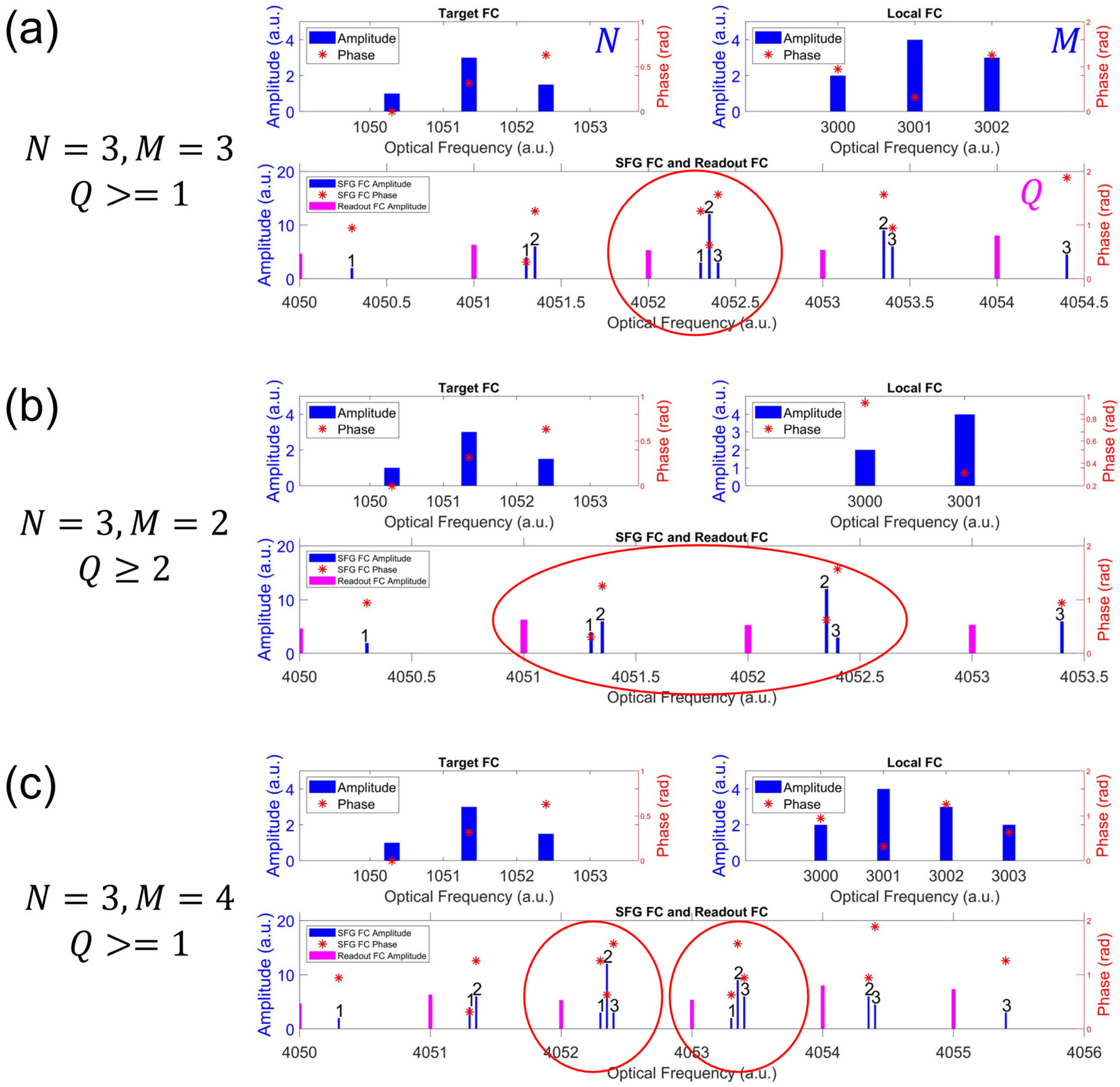

Supplementary Fig. 7| Bandwidth requirements for local FC and readout FC. M, N and Q denote the number of comb teeth for the target, local, and readout FCs, respectively. **a.** M=N=3, Q must be >= 1. The only complete SFG group, together with its primary readout tooth, is circled in red. **b.** N=2, M=3, Q must be >=2. Two incomplete SFG groups circled in red need to be read out by two readout teeth to map all three target teeth. **c.** N=3, M=4, Q must be >=1. Two complete SFG groups, together with their primary readout teeth, are circled in red.

As shown in the panel (a), when $M = N$, there is only one complete group (circled in red) formed in the SFG FC, which alone contains the information from all target teeth. Thus, to read all target information out, one readout tooth is required at minimum (R>=1), where the equality holds if and only if the readout tooth is the primary (or secondary) readout tooth of that complete group.

If we have one less local tooth (M=2, panel (b)), there is no complete group formed in the SFG FC, and at least two readout teeth are needed to read all three target teeth out (Q>=2). Similarly, to make the equality hold, the readout teeth need to be the primary (or secondary) readout teeth for those two center SFG groups, which are circled in red.

When there is one more local tooth relative to the number of target teeth (M=4, panel (c)), there will be two complete groups (circled in red) formed in the SFG FC. As in the case of L=3, one readout tooth is enough to read out all the target information (Q>=1). However, because of the availability of more complete groups, the requirement of the location of the single readout tooth to make the equality hold is more relaxed compared to the case of M=3. Here, it can be the primary (or secondary) readout tooth of either complete group.

This discussion can be generalized to any large number of teeth, although the various cases are demonstrated only in small numbers here for simplicity. In short, to realize the one-to-one mapping of all target teeth, the minimum required aggregate bandwidth of the local and readout FCs needs to be equal to or greater than that of the target FC, i.e., $M + Q \geq (N + 1)$. Note that there are two trade-offs behind this equation:

a. The trade-off between the local tooth number and readout tooth location. If there are more local teeth, the location (frequency) of the readout teeth can be more flexible since there are more complete groups formed. Conversely, the requirement of the readout tooth location will be stricter if there are fewer local teeth. In practice, it is generally much more difficult to accurately control the frequency of the readout teeth with the precision of the repetition rate than to obtain more local/readout teeth. Therefore, the general practical solution could be to make the aggregate bandwidth of local and readout FC moderately larger than that of the target FC and to roughly control the frequency of the readout comb (e.g., with the precision of 0.1 nm). This is what we do in the experiment.
b. The trade-off between the number of teeth of the local FC and readout FC. As the equation suggests, fewer readout teeth are needed if there are more local teeth, and vice versa. It should be noted that, although in theory only the sum of the bandwidth of local FC and readout FC is regulated to realize the one-to-one mapping of the target teeth, a relatively broad local FC (short local pulse) will be more beneficial in practice, as it can provides a better time gating (Section 1.2 and 4.2) and a higher upconversion efficiency (Section 4.1).

2.7 Bandwidth requirements for repetition rates and carrier–envelope offset frequency (CEO) frequencies

In the last subsection, we discuss the bandwidth requirements on optical side. In this subsection, we discuss instead the requirements on RF side, specifically, $f_{r,L}$, $f_{r,T}$, δ, $f_{ceo,L}$ and $f_{ceo,T}$. Without loss of generality, we continue the assumption that $f_{ceo,L} = 0$; thus, $f_{ceo,T}$ is effectively the relative f_{ceo} between the target FC and local FC.

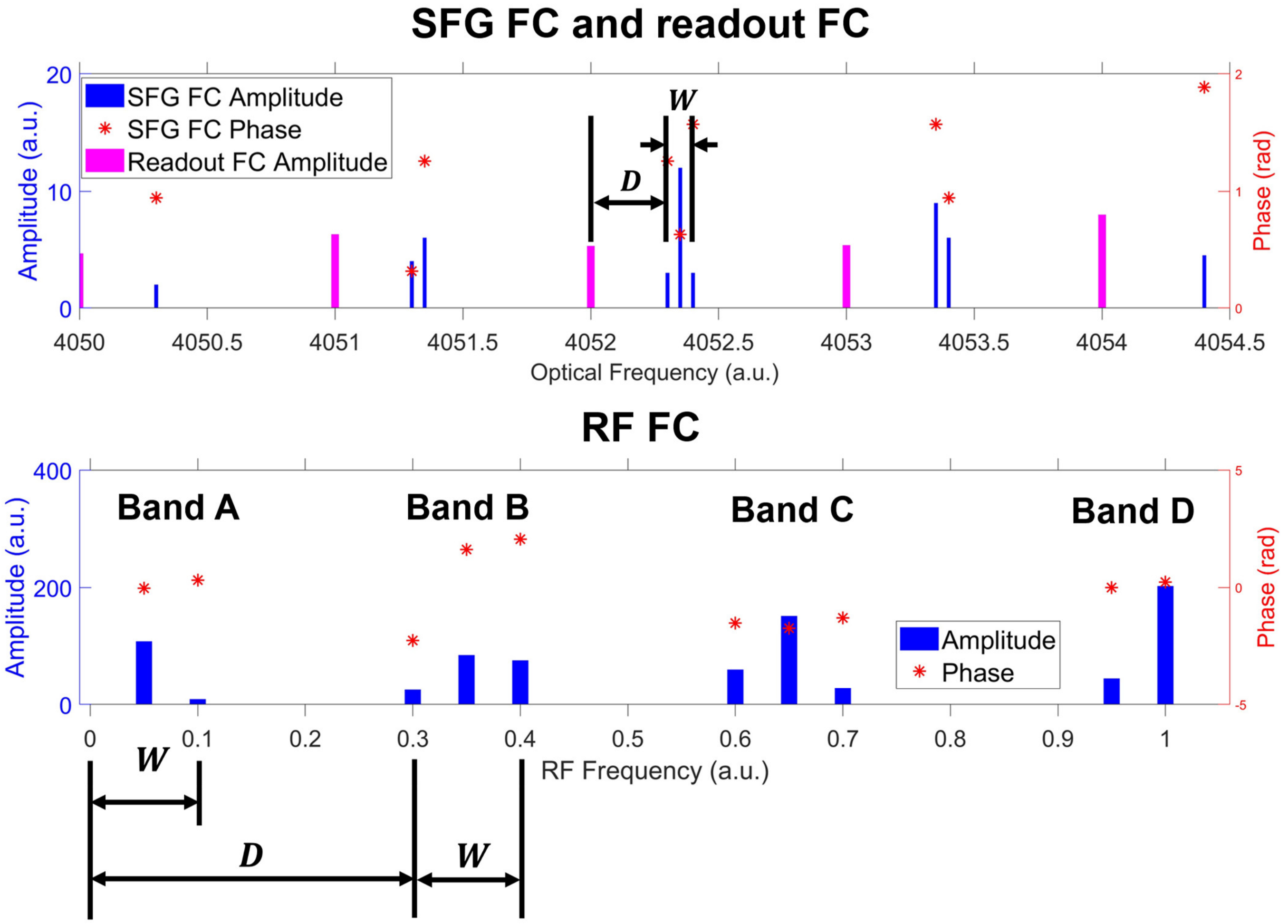

Supplementary Fig. 8| Bandwidth requirements for RF frequencies. The SFG FC and readout FC are from the Supplementary Fig. 6. D: the spectral distance (RF frequency) from the first tooth of an SFG group to its primary readout tooth. W: the spectral width of one complete group.

To quantify the requirements, here we define two important parameters (see the illustration in Supplementary Fig. 8):

a. The spectral (frequency) distance from the first tooth of an SFG group to its primary readout tooth, denoted by D. Note that the "first tooth of an SFG group" refers to the SFG tooth that corresponds to the first target tooth (the tooth with minimum frequency in the target FC).

$$D = mod\left(\left(n_{first} f_{r,T} + f_{ceo,T}\right), f_{r,L}\right) \quad (17)$$

$mod(A, B)$ denotes the remainder after division of dividend A by divisor B, and $\left(n_{first} f_{r,T} + f_{ceo,T}\right)$ is the optical frequency of the first tooth of the target FC.

b. The spectral width of one complete group, denoted by W.

$$W = \left(n_{last} - n_{first}\right)\delta = BW_T \times \frac{\delta}{f_{r,T}} \quad (18)$$

BW_T denotes the optical bandwidth of target FC.

Additionally, to realize a one-to-one mapping, two kinds of spectral overlap need to be avoided:

a. Avoiding overlap between band A(D) and band B(C), which requires:

$$D > W \quad (19)$$

b. Avoiding overlap between band B and band C, which requires:

$$D + W < \frac{f_{r,L}}{2} \quad (20)$$

Similar to dual-comb spectroscopy (DCS), $\frac{\delta}{f_{r,T}}$ needs to be small enough to provide enough bandwidth in the RF domain, i.e., to satisfy the requirement b. In addition, $f_{ceo,T}$ also need to be determined carefully to satisfy requirement a, which is different with DCS.

Note that the above bandwidth requirements are effective when a single detector is used for heterodyne photodetection. For the case that an ideal balanced detector is used, the requirements are simplified to only one equation:

$$W < \frac{f_{r,L}}{2} \tag{21}$$

This is because the band A and band D are eliminated by the balanced detector since they are common-mode signal from the SFG FC. In another word, the balanced detector can double the bandwidth for RF band B (C) assuming unchanged $\frac{\delta}{f_{r,T}}$, which makes the RF bandwidth requirement effectively same as the general dual-comb.

3. **Comparison of principle between different techniques**

In this section, we will compare DCS, C.W. upconversion spectroscopy, electric-optic sampling (EOS), and cross-comb spectroscopy (CCS) (Supplementary Fig. 9) using simple mathematical descriptions. Then, we will demonstrate that C.W. upconversion and EOS are essentially two special cases of the cross-comb; the former uses a very narrow-band local "FC" with only one "comb tooth", and the latter uses a very broadband local FC (very short local pulse) which also functions as the readout FC. We will describe them in both the time domain and the frequency domain. Especially, we show that the CCS in a general configuration can utilize the optical bandwidth in a more efficient way, compared to EOS. In all these techniques, if the full electric field profile of the readout FC (local FC) is available, generally acquired by field-resolved measurements (e.g., FROG), the electric field of the target FC can also be reconstructed based on measured correlation signal. This extra information could be helpful in some ways; however, it is not necessary for the goal of general absorption spectroscopy.

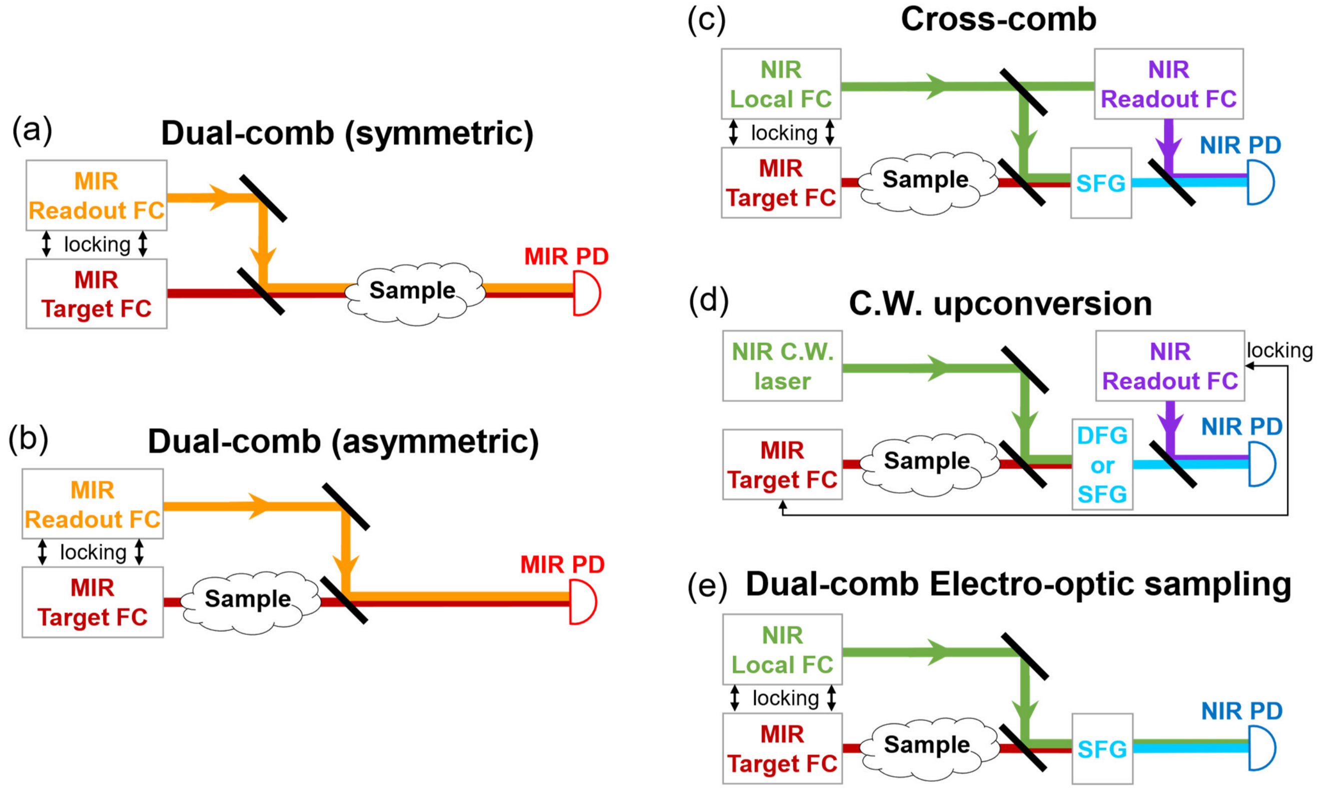

Supplementary Fig. 9| Simplified schematics of different techniques. Note that generally balanced detectors are used, which are simplified to be single detectors in the schematics. Also, there may be additional equipment before the detector, which is also omitted here; for example, an ellipsometry setup for electro-optic sampling (e).

To begin with, let us review the cross-correlation theorem:

$$C(\tau) = f(t) \otimes h(t) = \int_{-\infty}^{+\infty} f^*(t) h(t+\tau) dt \quad \Rightarrow \quad \mathcal{F}\{C(\tau)\} = F^*(\omega) H(\omega) \tag{22}$$

Or equally:

$$C(\tau) = f(t) \otimes h(t) = \int_{-\infty}^{+\infty} f(t) h^*(t-\tau) dt \quad \Rightarrow \quad \mathcal{F}\{C(\tau)\} = F(\omega) H^*(\omega) \tag{23}$$

Where $F(\omega)$ and $H(\omega)$ denote the Fourier transform of $f(t)$ and $h(t)$, respectively.

3.1 DCS

Firstly, for DCS with a symmetric (collinear) configuration (Supplementary Fig. 9(a))[9],

$$c(\tau) = \int_{-\infty}^{+\infty} e_T(t) e_R^*(t-\tau) dt \tag{24}$$

$$C(\omega) = \mathcal{F}\{c(\tau)\} = E_T(\omega) E_R^*(\omega) \tag{25}$$

where $e_T(t)$ and $e_R(t)$ denote the electric field of the target FC (pulse) and readout FC without passing the sample (passing the reference cell), while $E_T(\omega)$ and $E_L(\omega)$ denote their Fourier transform, respectively. $c(\tau)$ denotes the cross-correlation signal measured by the detector in the time domain, and $C(\omega)$ is its Fourier transform in the frequency domain.

Let assume the sample's spectral response is $S(\omega)$, including both spectral amplitude $|S(\omega)|$ and spectral phase $Ang\ (S(\omega))$. If we use $e(t)$ and $e'(t)$ to denote the electric field of a pulse before and after passing the sample, we have:

$$\mathcal{F}\{e'(t)\} = S(\omega)\mathcal{F}\{e(t)\} = S(\omega)E(\omega) \tag{26}$$

Therefore, for the cross-correlation signal $c'(\tau)$, measured when the target pulse and readout pulse pass the sample:

$$c'^{(\tau)} = \int_{-\infty}^{+\infty} e'_T(t) e'^*_R(t-\tau) dt \tag{27}$$

$$C'(\omega) = \mathcal{F}\{c'(\tau)\} = E_T(\omega)S(\omega)E_R^*(\omega)S'(\omega) = E_T(\omega)E_R^*(\omega)|S(\omega)|^2 \tag{28}$$

By comparing those two measurements (with and without sample), we have:

$$D(\omega) = \frac{C'(\omega)}{C(\omega)} = |S(\omega)|^2 \tag{29}$$

$D(\omega)$ denotes the comparison between those two measurements. It shows that this measurement can only provide spectral intensity of the sample's response, which lacks the phase information.

In fact, a symmetric DCS measurement is essentially a traditional FTIR (Michelson interferometer), which gives information only about spectral intensity but not spectral phase. Therefore, one cannot get any temporal information on the target pulses which are disturbed by the sample. In other words, the correlation signal $c(\tau)$ is independent of the spectral phase of $e'_T(t)$, which is cancelled as the readout pulse also passes the sample.

Secondly, for DCS with an asymmetric (dispersive) configuration (Supplementary Fig. 9(b)),

$$c(\tau) = \int_{-\infty}^{+\infty} e_T(t) e_R^*(t-\tau) dt \tag{30}$$

$$C(\omega) = \mathcal{F}\{c(\tau)\} = E_T(\omega) E_R^*(\omega) \tag{31}$$

$$c'^{(\tau)} = \int_{-\infty}^{+\infty} e'_T(t) e_R^*(t-\tau) dt \tag{32}$$

$$C'^{(\omega)} = \mathcal{F}\{c'^{(\tau)}\} = E_T(\omega)S(\omega)E_R^*(\omega) \tag{33}$$

$$D(\omega) = \frac{C'(\omega)}{C(\omega)} = S(\omega) \tag{34}$$

Note that in this configuration, the readout pulse does not pass the sample before being combined with the target pulse. In this case, the measured $D(\omega)$ is dependent on the phase of $S(\omega)$; thus, one can get phase information of the sample response.

However, one still cannot recover the full electric field of the target pulse, $e_T(t)$ (or $e'_T(t)$) only by measurement of $C(\omega)$ (or $C'(\omega)$), in which $E_T(\omega)$ (or $E'_T(\omega)$) is modulated by $E_R^*(\omega)$. This is because $E_R^*(\omega)$ is generally unknown unless some other field-resolved measurements (e.g., FROG) are applied to measure it. Nonetheless, general absorption spectroscopy does not

require the full knowledge of $e_T(t)$, since what we need to measure is $S(\omega)$ rather than $E_T(\omega)$, assuming $e_R(t)$ does not change for measurements with and without the sample.

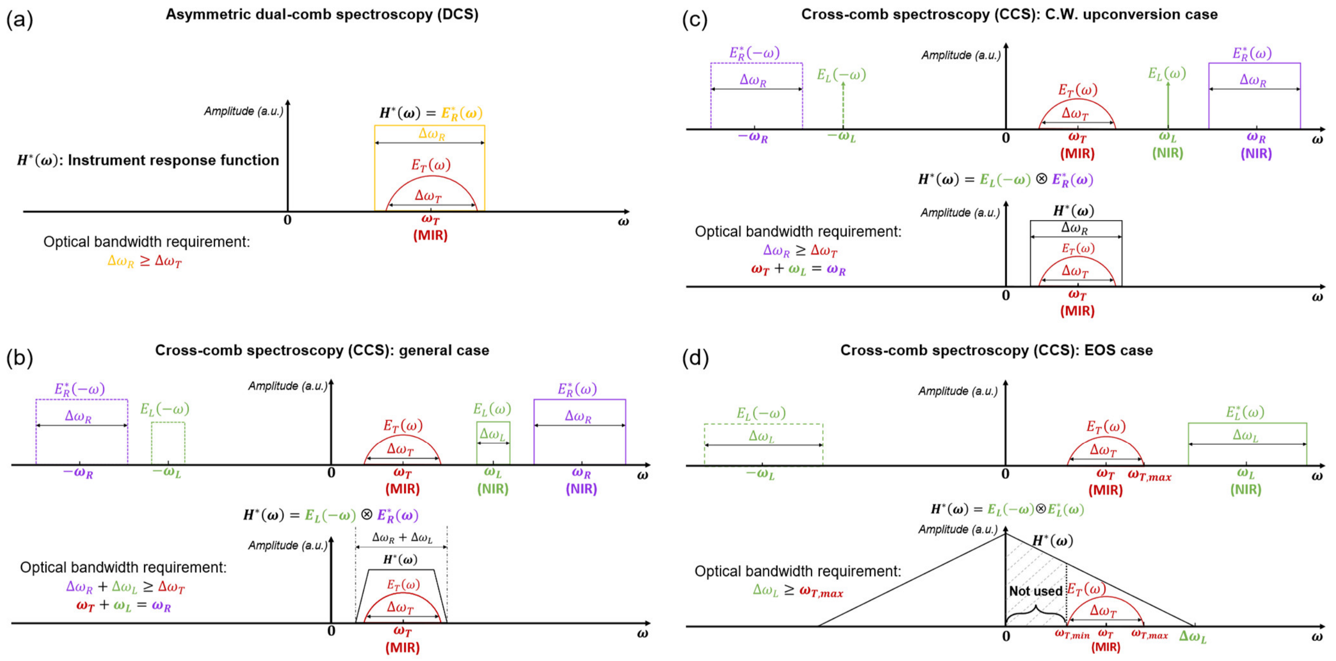

Supplementary Fig. 10| Instrument response function of different techniques. $\omega_T, \omega_L, and\ \omega_R$: spectral center of the target FC, local FC, and readout FC. $\Delta\omega_T, \Delta\omega_L, and\ \ \Delta\omega_R$: optical bandwidth of target FC, local FC, and readout FC. Note that we only plot the spectral amplitude (e.g., $|E_L(\omega)|$) for each function and assume zero spectral phase for all of them. Also, the spectral profiles of the local and readout FC ($E_L(\omega), E_R(\omega)$) are simplified to rectangular functions for clarity. **a.** asymmetric DCS. **b.** general CCS. The bandwidth requirement agrees with the result of our derivation in Section 2 in which comb teeth are included. **c.** CCS (C.W. upconversion case). **d.** CCS (EOS case). $\omega_{T,max}$ ($\omega_{T,min}$), the maximum (minimum) frequency of the target spectrum.

Supplementary Figure 10(a) illustrates the spectral amplitudes of $E_T(\omega)$ and $E_R^*(\omega)$ as well as the optical bandwidth requirement for the readout FC. $H^*(\omega)$ denotes the response function of the instrument, which is simply equal to $E_R^*(\omega)$ in this case. Note that this illustration, as well as the following illustrations for other techniques, depicts only the spectral envelopes and thus does not account for individual comb lines. This description simplifies the math without losing any generality since the comb lines can be understood effectively as sampling the envelopes in frequency domain (or equivalently, as a periodic extension in the time domain). The full description of CCS which factors in comb lines is presented in Section 2.

3.2 CCS

Thirdly, let us discuss CCS, which has the additional step of frequency conversion (Supplementary Fig. 9(c)).

Step 1: nonlinear upconversion

$$e_{SFG}(t,\tau) = e_T(t)e_L(t-\tau) \tag{35}$$

where $e_L(t)$ denotes the electric field of the local FC (pulse). Note that this equation is approximated that needs to be based on proper assumptions and approximations, the main of which include slowly varying envelop approximation (SVEA), weak nonlinearity, medium without loss and dispersion, unaffected input beams, and ideal phase-matching. In addition, for clarity, we omit all the proportionality constants since we are mainly interested in the shape of the pulses/spectra. Those conventions are same as what are generally used in the community of ultrafast pulse measurement[10], where more details can be found.

Step 2: linear readout (same as asymmetric DCS)

$$c(\tau) = \int_{-\infty}^{+\infty} e_{SFG}(t,\tau)e_R^*(t-\tau)dt = \int_{-\infty}^{+\infty} e_T(t)e_L(t-\tau)e_R^*(t-\tau)dt \tag{36}$$

Let $h(t) = e_L^*(t)\, e_R(t)$, we can rewrite the above equation as:

$$c(\tau) = \int_{-\infty}^{+\infty} e_T(t) h^*(t-\tau)\, dt \tag{37}$$

$$C(\omega) = \mathcal{F}\{c(\tau)\} = E_T(\omega) H^*(\omega) \tag{38}$$

Above is the result for the measurement without sample, and for the measurement with sample we have:

$$c'^{(\tau)} = \int_{-\infty}^{+\infty} e'_T(t) h^*(t-\tau)\, dt \tag{39}$$

$$C'^{(\omega)} = \mathcal{F}\{c'^{(\tau)}\} = E_T(\omega) S(\omega) H^*(\omega) \tag{40}$$

$$D(\omega) = \frac{C'(\omega)}{C(\omega)} = S(\omega) \tag{41}$$

Like asymmetric DCS, one can get phase information of the sample response, but $e_T(t)$ cannot be fully recovered since $E_T(\omega)$ is modulated by $H^*(\omega)$ in $C(\omega)$. However, this does not impede the measurement of the absorption spectrum $S(\omega)$.

In this case, the response function of the instrument is $H^*(\omega)$, based on that $h(t) = e_L^*(t)\, e_R(t)$, we have:

$$H^*(\omega) = E_L(-\omega) \otimes E_R^*(\omega) \tag{42}$$

which is illustrated in Supplementary Fig. 10(b).

3.3 C.W. upconversion and EOS

Both C.W. upconversion and EOS can be shown to be special cases of the above CCS description. To describe C.W. upconversion (Supplementary Fig. 9(d)), nothing needs to be modified in the CCS equations, except that $e_L(t-\tau)$ denotes a continuous sinusoidal wave instead of a pulse. Also, it should be noted that, using an SFG or DFG process for nonlinear upconversion does not make a fundamental difference here; the equations are equivalent up to a complex conjugation. The illustration is shown in Supplementary Fig. 10(c).

EOS (Supplementary Fig. 7(e)) requires a more careful discussion. Let us start with equations of CCS.

Step 1: nonlinear upconversion

$$e_{SFG}(t,\tau) = e_T(t) e_L(t-\tau) \tag{43}$$

In the case of ideal EOS, $e_L(t)$ is much shorter than $e_T(t)$. In other words, in the temporal span of $e_L(t)$, $e_T(t)$ varies very little and can be approximated to be constant. Thus, we have:

$$e_{SFG}(t,\tau) = \boldsymbol{e_T(t)} e_L(t-\tau) \cong \boldsymbol{e_T(\tau)} e_L(t-\tau) \tag{44}$$

Another way to interpret this is that $e_L(t)$ is approximated to be a Dirac delta function ($\delta(t-\tau)$) that samples $e_T(t)$ in the time domain.

With this approximation, we can continue to derive the next readout step. Note that in EOS the role of readout pulse is played by the local pulse itself.

Step 2: linear readout

$$c(\tau) = \int_{-\infty}^{+\infty} \boldsymbol{e_T(t)} e_L(t-\tau) e_R^*(t-\tau)\, dt \cong \int_{-\infty}^{+\infty} \boldsymbol{e_T(\tau)} e_L(t-\tau) e_L^*(t-\tau)\, dt = \boldsymbol{e_T(\tau)} \int_{-\infty}^{+\infty} e_L(t-\tau) e_L^*(t-\tau)\, dt = K\boldsymbol{e_T(\tau)} \tag{45}$$

$$C(\omega) = \mathcal{F}\{c(\tau)\} = K E_T(\omega) \tag{46}$$

$$C'^{(\omega)} = \mathcal{F}\{c'(\tau)\} = K E_T(\omega) S(\omega) \tag{47}$$

$$D(\omega) = \frac{C'(\omega)}{C(\omega)} = S(\omega) \tag{48}$$

where K denotes the constant that equals to the integration $\int_{-\infty}^{+\infty} e_L(t-\tau)e_L^*(t-\tau)dt$, the core of which is independent of the parameter delay τ. As shown in the equation, under this approximation, the correlation signal $c(\tau)$ is equal to the electric field of target pulse $e_T(\tau)$ up to a constant. Thus, under the approximation of the ideal local pulse (infinitely short pulse width), one can obtain the full electric field of the target pulse $e_T(t)$ in addition to the absorption spectrum $S(\omega)$.

In practice, the finite pulse duration of the sampling pulse always imposes a frequency-dependent instrument response[11,12], which is illustrated in Supplementary Fig. 10(d). In this case, the instrument response function $H^*(\omega)$ is the "autoconvolution" of the local spectrum.

$$H^*(\omega) = E_L(-\omega) \otimes E_L^*(\omega) \tag{49}$$

In contrast to DCS and CCS, EOS needs the bandwidth of the local FC ($\Delta\omega_L$) to be equal or larger than the maximum frequency of the target FC ($\omega_{T,max}$) to detect the full spectrum of the target FC. This explains why EOS requires a much broader optical bandwidth compared to DCS and CCS. However, the $H^*(\omega)$ band below the minimum frequency of the target FC ($\omega_{T,min}$), is not effectively utilized, resulting from the fact that the same continuous FC is used as both the readout and local FC.

3.4 C.W. upconversion and EOS described by comb-teeth mapping

In the previous subsection, we have described C.W. upconversion spectroscopy and dual-comb EOS using the language of CCS without including comb teeth. In this subsection, we do the same thing factoring in comb teeth, following the derivation in Section 2.5. Note that Fig. 4 of the main paper is a good illustration for this subsection.

Based on what we derived for RF band B in Section 2.5, we can write the general formula for j_{th} target tooth mapped in RF band B:

$$I_j = \left(\sum_{m=1}^{M} L_m R_{m+j}^*\right) X_j \tag{50}$$

where M denotes the total number of local teeth. Note that all the subscripts denote effective tooth index.

For the case of C.W. upconversion, there is only one "local tooth", so the formula is simplified to be

$$I_j = L_1 R_{m+1}^* X_j \tag{51}$$

Everything can be described well by the language of CCS.

For the case of ideal EOS, let us review the approximation that we made in the time domain, which is:

"In the span of $e_L(t)$ or $e_R(t)$ (very short local/readout pulse), $e_T(t)$ (target pulse) varies slowly, and thus can be approximated as constant."

Correspondingly, in the frequency domain, we can have such an equivalent approximation:

"In the span of $E_T(\omega)$ (very narrowband, relatively), $E_L(\omega)$ or $E_R(\omega)$ (very broadband, relatively) varies slowly and can be approximated as constant."

With this approximation, we have:

$$R_m \cong R_{m+1} \cong R_{m+2} \cong R_{m+3} \dots\dots \cong R_{m+N} \tag{52}$$

where N denote the total number of target teeth. Thus, we have:

$$for\ any\ j, \sum_{m=1}^{M} L_m R_{m+j}^* \cong K \tag{53}$$

where K denotes a constant.

$$I_j = \left(\sum_{m=1}^{M} L_m R_{m+j}^*\right) X_j \cong K X_j, for\ any\ j \tag{54}$$

This result is equivalent to the equation $C(\omega) = \mathcal{F}\{c(\tau)\} = KE_T(\omega)$, which we derived in the last subsection in the time domain. Both results show that, in the limit of ideal EOS, the measured correlation signal (RF heterodyne beating) is equal to the electric field of the target pulse up to a constant.

The case of nonideal EOS is well demonstrated in reference [5].

In summary, both C.W. upconversion spectroscopy and EOS fall into the category of CCS, representing two opposite limits on the bandwidth of the local comb.

4. **Comparison of performance between different techniques, by theoretical model**

In the last section, we compared how different techniques work. With the same model and assumptions, in this section, we further compare some of their important metrics, including detection bandwidth, efficiency, SNR and dynamic range. We will first compare detection bandwidth and efficiency of different upconversion methods, pointing out that the short-pulse CCS is overall more efficient. Secondly, we will present a comparison between DCS and short-pulse CCS in terms of SNR and dynamic range, highlighting the effect of temporal gating[3]. Lastly, some insights into the design rules of CCS systems are provided, based on the results of this section.

4.1 Detection bandwidth and efficiency of upconversion methods

4.1.1 General CCS, symmetric CCS, and C.W. upconversion CCS

In last section, we defined a response function $H(\omega)$ to describe different methods. Here, we will continue to use it for a more quantitative comparison. With the same mathematical assumptions as before (Supplementary Fig. 10), the dimensions of $H(\omega)$ of the general CCS case are calculated, based on the given parameters (heights and width) of $E_L(\omega)$ and $E_R(\omega)$ (Supplementary Fig. 11(a)).

In practice, it is more general and convenient to discuss and compare spectral intensity (power) instead of spectral amplitude of the electric field. Thus, the amplitude spectrum is squared to the intensity spectrum, and a power gain function $G(\omega) = |H^*(\omega)|^2$ is defined to describe the detection efficiency of the system (Supplementary Fig. 11(b)).

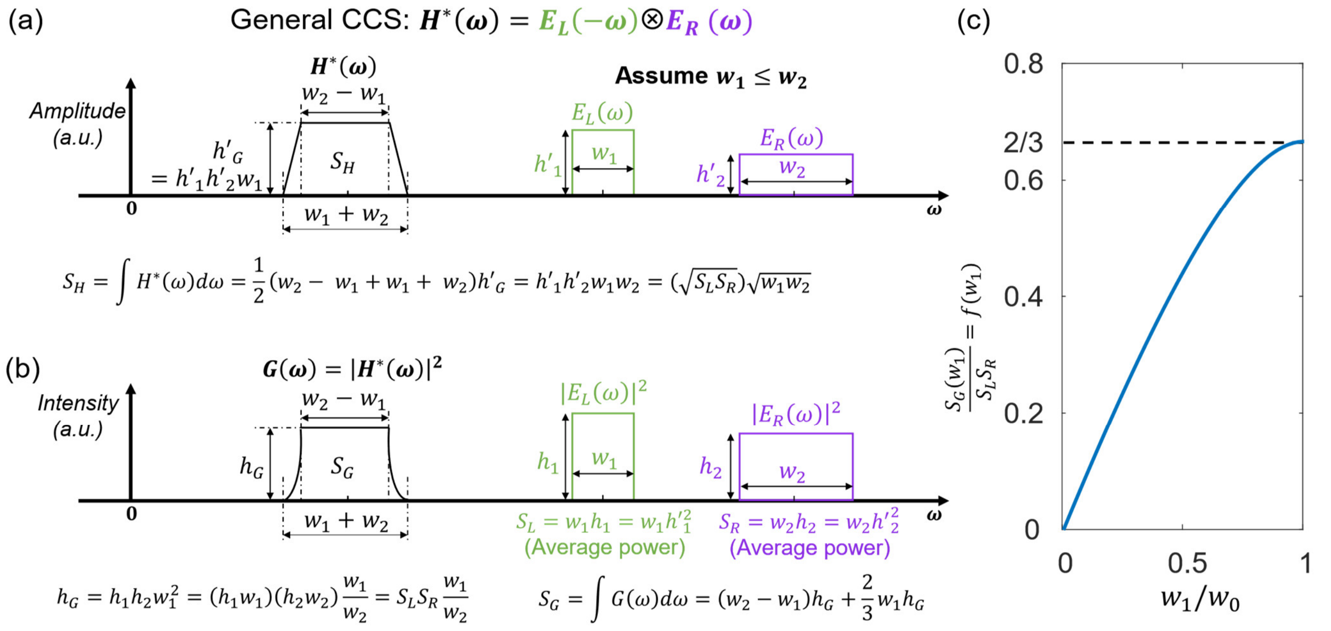

Supplementary Fig. 11| Instrument response function and gain function of general CCS. a. Instrument response function $H(\omega)$ spectral amplitude of $E_L(\omega)$ and $E_R(\omega)$. **b.** Detection gain function $G(\omega)$ and spectral intensity of $E_L(\omega)$ and $E_R(\omega)$. $S_{L(R)} = \int |E_{L(R)}(\omega)|^2 d\omega$. w and h denote the width and height of those spectral profiles, respectively. We assume w_1 (bandwidth of local FC) $\le w_2$ (bandwidth of readout FC) in this derivation, with no lack of generality for conclusions we draw. **c.** $\frac{S_G(w_1)}{S_L S_R} = f(w_1) = \left(2w_0 - \frac{4}{3}w_1\right)\frac{w_1}{2w_0 - w_1}$. This function shows how S_G scales with w_1 under the assumptions of constant combined power and combined bandwidth of local FC and readout FC. It monotonically increases with w_1 on the interval [0, w_0].

Three metrics of $G(\omega)$ are used to quantify it:

h_G: the maximum value ("height") of $G(\omega)$, which describe the highest detection gain at the center part of $G(\omega)$.

w_G: the bandwidth of $G(\omega)$. Here we simply use zero points to define the width.

S_G: the area under $G(\omega)$, i.e., $\int G(\omega)d\omega$. This quantifies the total gain of the system.

Under our assumption of rectangular spectral profiles for $E_L(\omega)$ and $E_R(\omega)$, we get:

$$h_G = h_1 h_2 w_1^2 = (h_1 w_1)(h_2 w_2)\frac{w_1}{w_2} = S_L S_R \frac{w_1}{w_2} \tag{55}$$

$$BG = w_1 + w_2 \tag{56}$$

$$S_G = \int G(\omega)d\omega = (w_2 - w_1)h_G + \frac{2}{3}w_1 h_G = \left(w_2 - \frac{1}{3}w_1\right)h_G = \left(w_2 - \frac{1}{3}w_1\right)\frac{w_1}{w_2}S_L S_R \tag{57}$$

where we assume $w_1 \leq w_2$.

Please refer to Supplementary Fig. 11 and its caption for a detailed definition of variables. Note that $S_{L(R)}$, which denotes the area under $\left|E_{L(R)}(\omega)\right|^2$, is equivalent to the average power of the local (readout) FC.

There are in total four effective free variables: $S_L, S_R, w_1, and\ w_2$. For reasonable comparison later, let us assume a fixed total bandwidth $w = 2w_0 = w_1 + w_2$ and a fixed total power $S = 2S_0 = S_L + S_R$ for the local and readout FC.

Let us first consider the choice of S_L and S_R. Based on the equation of h_G, we have

$$S_L S_R \leq \left(\frac{S_L + S_R}{2}\right)^2 = S_0^2,, where\ "="\ holds\ when\ S_L = S_R = S_0 \tag{58}$$

Thus, we want to make $S_L = S_R = S_0$ to optimize h_G (this also optimizes S_G). In this case, $S_L S_R = S_0^2$, which is a constant.

Secondly, let us consider the choice of w_1 and w_2. To optimize h_G, it is obvious that we want to make $w_1 = w_2 = w_0$, thus we have $h_G = S_L S_R = S_0^2$.

As for S_G, a more careful calculation is needed. Since $S_L S_R$ is already set to a constant, let us consider function $f(w_1, w_2) = \left(w_2 - \frac{1}{3}w_1\right)\frac{w_1}{w_2}$ with the constraint that $w_1 + w_2 = w = 2w_0$, by which $f(w_1, w_2)$ is effectively a function of one variable (w_1 or w_2). Making it a function of only w_1, we plot $f(w_1)$ in Supplementary Fig. 11(c), which demonstrates how S_G scales with w_1 under our assumptions here. $f(w_1)$ monotonically increases with w_1 on the interval [0, w_0], and reaches its maxima of $2/3$ at $w_1 = w_2 = w_0$. Based on these observations, we name the case where $S_L = S_R = S_0$ and $w_1 = w_2 = w_0$ as "symmetric CCS."

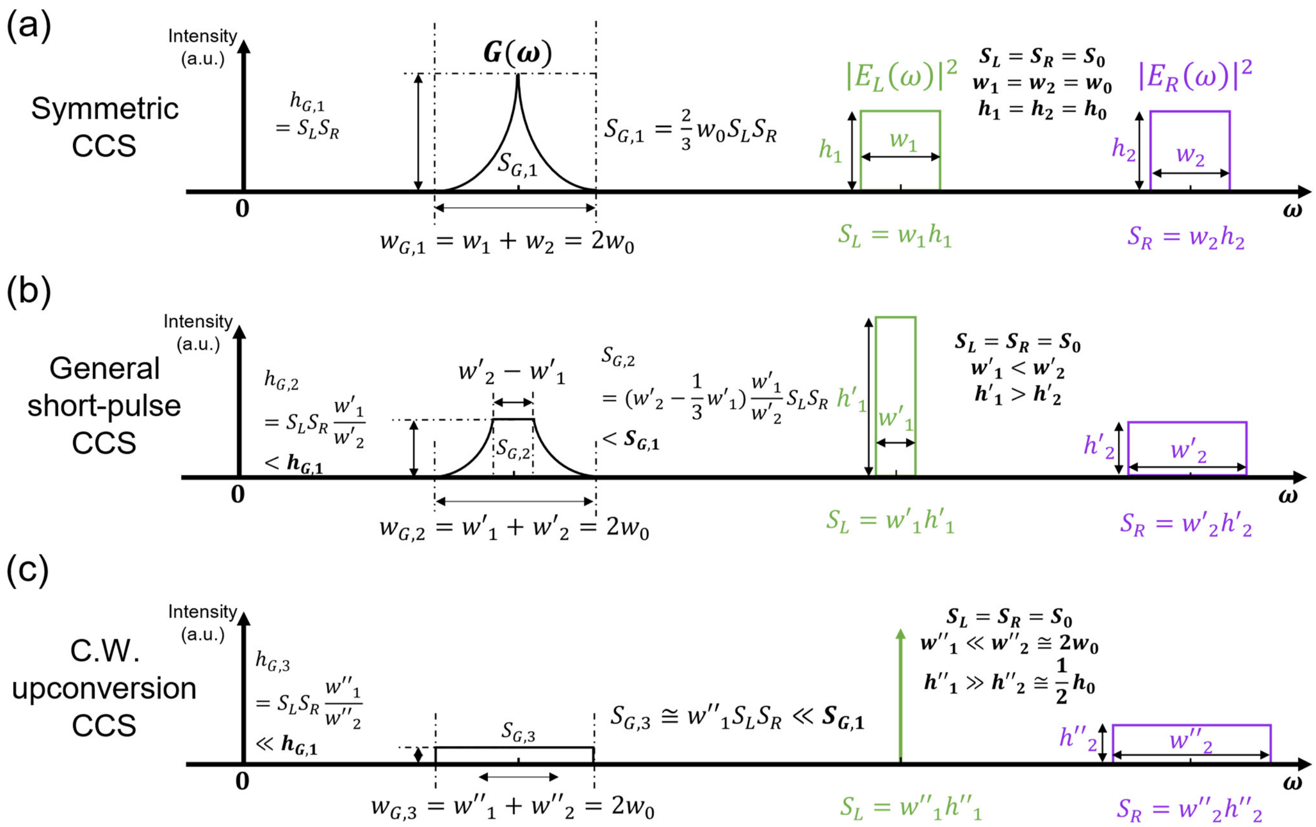

Supplementary Fig. 12| Gain function $G(\omega)$ of symmetric CCS (a), general CCS (b), and C.W. upconversion CCS (c). In (c), the local FC spectral intensity profile is like a Dirac-delta function, which has a very small width w''_1 and a very large height h''_1, while their product S_L is kept same as the other two cases.

With the derivation above, it is easy to compare the symmetric CCS, general short-pulse CCS (with parameters close to symmetric CCS) and C.W. upconversion CCS, shown in Supplementary Figure. 12 (a)-(c), respectively. Note that we keep the total bandwidth and power of local and readout FC the same for all three cases. As the bandwidth of the local FC shrinks, symmetric CCS becomes general short-pulse CCS, which finally becomes C.W. upconversion CCS. During the transition, although the bandwidth w_G stays the same, the maximum gain h_G and the total area S_G monotonically decrease. For the C.W. upconversion case (panel c), the bandwidth of the local FC shrinks to w_1'' that is $\ll w_0$. If we compare h_G and S_G between symmetric case and C.W. upconversion case, we have:

$$\frac{h_{G,sym}}{h_{G,C.W.}} = \frac{h_{G,1}}{h_{G,3}} = \frac{2w_0}{w_1''} \gg 1 \tag{59}$$

$$\frac{S_{G,sym}}{S_{G,C.W.}} = \frac{S_{G,1}}{S_{G,3}} = \frac{2w_0}{3w_1''} \gg 1 \tag{60}$$

In short, general short-pulse CCS (where local and readout FC both have a broad bandwidth) has a much higher detection gain than C.W. upconversion CCS. This should not be a surprising result if we think about the comparison of conversion efficiency of general nonlinear optics processes between C.W. laser and short pulses. The enhancement ratio here is exactly equivalent to the peak power enhancement ratio between coherent short pulses and a C.W. laser with the same average power. The conclusion we arrive at here has its roots in the same reason why people prefer short pulses over C.W. lasers for nonlinear optics: the much higher peak power of short pulses.

4.1.2 Symmetric CCS and EOS CCS

Here we compare short-pulse CCS with EOS CCS, the former of which is represented by the symmetric CCS, and the comparison is illustrated in Supplementary Figure. 13. Still, we keep their total bandwidth and power the same to make a fair comparison.

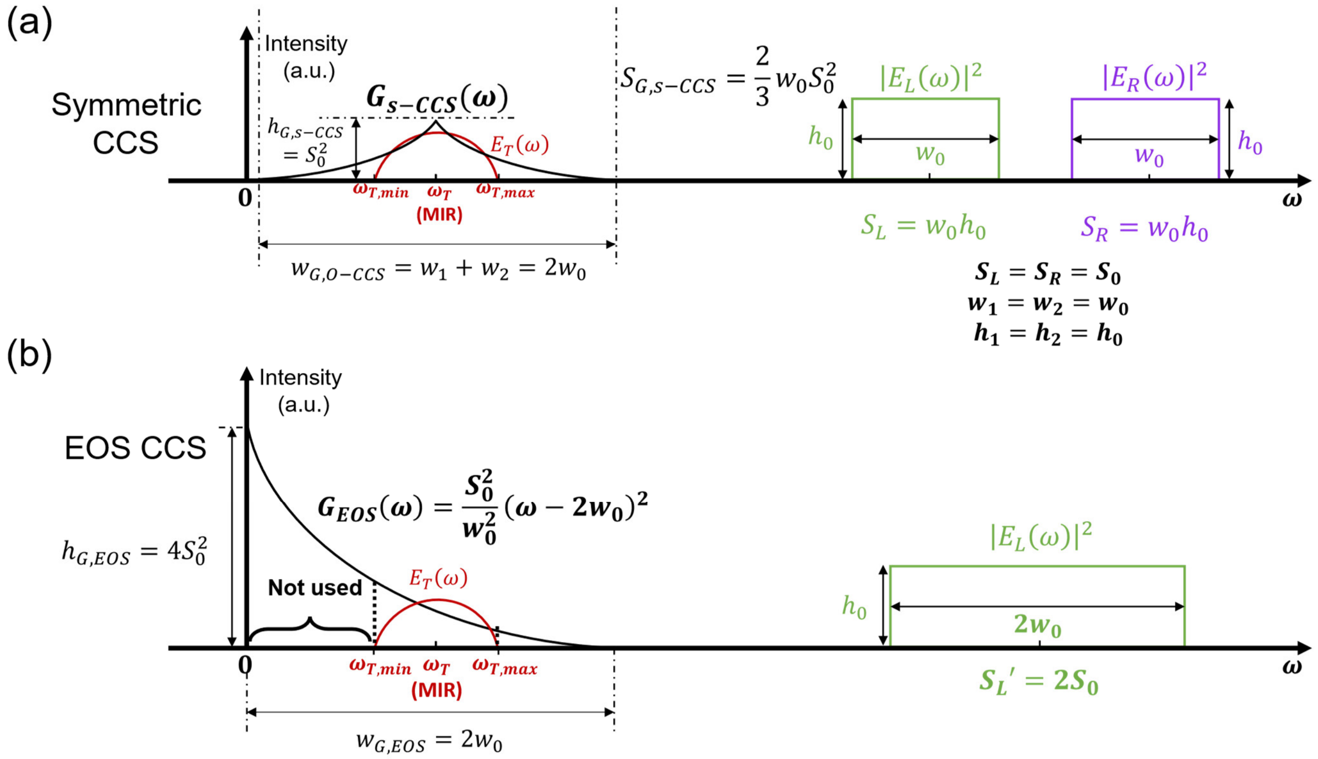

Supplementary Fig. 13| Gain function $G(\omega)$ of symmetric CCS (a) and EOS CCS (b). Note that the two panels are not on the exact same Y scale. As denoted in the figure, the heights of $G(\omega)$ are $h_{G,s-CCS} = S_0^2$ and $h_{G,s-CCS} = 4S_0^2$, respectively (The latter is four times of the former).

Based on the calculation shown in the plot, although the maxima of $G_{EOS}(\omega)$, i.e., $h_{G,EOS}$, is larger than that of the $G_{ccs}(\omega)$ i.e., $h_{G,s-CCS}$, the $h_{G,EOS}$ is at $\omega = 0$, which cannot overlap with target spectrum at all. Actually, $G_{EOS}(\omega)$ monotonically decreases with ω, and the largest part of it in amplitude, i.e., $\omega \in [0, \omega_{T,min}]$, is not effectively used, as shown in the Supplementary Fig. 10. A system response curve with a similar shape and trend has been calculated in ref [11] (see Fig. S1 in its Supplementary Materials). This qualitative behavior already shows that EOS uses resources in a less efficient way compared to general CCS. Nevertheless, we still proceed to give a more quantitative description.

For a fair comparison, we want to calculate $G_{EOS}(\omega_{T,min})$ (the gain at $\omega = \omega_{T,min}$), $G_{EOS}(\omega_T)$ (the gain at the center of the target spectrum, $\omega = \omega_T$), and $S_{G,EOS} = \int_{\omega_{T,min}}^{\omega_{T,max}} G_{EOS}(\omega)d\omega$ (the overall gain that covers target spectrum). Based on the model presented before, we can have an analytical expression for $G_{EOS}(\omega)$:

$$G_{EOS}(\omega) = \frac{S_0^2}{w_0^2}(\omega - 2w_0)^2 \tag{61}$$

Of course, the value of the three metrics depends on the value of $\omega_{T,min}$, ω_T, and $\omega_{T,max}$, and different values can result in different conclusions for the comparison. Here, to give an example, let us adopt some values that are close to our experiments.

Let us set $\omega_T = 75\ THz$ ($4\ \mu m$) and assume we are going to detect a 30-THz-broad target bandwidth, that is, $\omega_{T,min} = 60\ THz$ ($5\ \mu m$) and $\omega_{T,max} = 90\ THz$ ($3.33\ \mu m$). If we set the center of the local FC at $200\ THz$ ($1.5\ \mu m$), the bandwidth of the local FC needs to be at least 90 THz, i.e., $w_0 = 45\ THz$, in order to detect the whole target spectrum. This suggests the local FC must span $155\ THz - 245\ THz$ ($1.22\ \mu m\ to\ 1.93\ \mu m$), and is therefore nontrivial to generate. Meanwhile, symmetric CCS only requires 15-THz local and readout FCs (in total 30 THz), which are much less challenging experimentally.

With the assumed values of $w_0, \omega_{T,min}, \omega_T$, and $\omega_{T,max}$, we have $\omega_{T,min} = \frac{4}{3}w_0, \omega_T = \frac{5}{3}w_0, and\ \omega_{T,max} = 2w_0$. Then the three metrics can be calculated and compared.

$$G_{EOS}(\omega_{T,min}) = \frac{4}{9}S_0^2;\quad G_{EOS}(\omega_T) = \frac{1}{9}S_0^2;\quad G_{EOS}(\omega_{T,max}) = 0; S_{G,EOS} = \int_{\omega_{T,min}}^{\omega_{T,max}} G_{EOS}(\omega)d\omega = \frac{8}{81}w_0 S_0^2 \tag{62}$$

$$\frac{G_{EOS}(\omega_{T,min})}{G_{s-CCS}(\omega_T)} = \frac{4}{9};\quad \frac{G_{EOS}(\omega_T)}{G_{s-CCS}(\omega_T)} = \frac{1}{9};\quad \frac{S_{G,EOS}}{S_{G,s-CCS}} = \frac{4}{27} \cong 0.15 \tag{63}$$

In this case, the maximum value of $G_{EOS}(\omega)$ is just 4/9 of that of the $G_{o-CCS}(\omega)$, and this value is only at the left edge of the target spectrum. If we instead compare the gain at the center of the two functions, the ratio becomes only 1/9. Since they are of different profiles, it is more reasonable to compare their overall gain S_G, and $S_{G,EOS}$ is only ~15% of $S_{G,O-CCS}$. In short, although EOS may require much more experimental effort, its overall detection efficiency can be much lower than symmetric (general short-pulse) EOS.

4.1.3 Comparison of General CCS, symmetric CCS, and C.W. upconversion CCS under a different assumption

In 4.1.1, we compare general CCS, symmetric CCS, and C.W. upconversion CCS under the assumption of constant combined power and bandwidth of local FC and readout FC. While that is a fair comparison in theory, it is not generally realistic since it requires changing the properties of both combs simultaneously, which could be experimentally challenging. Here we provide another way of comparison under a different assumption that we keep the readout FC always the same ($w_R = w_0$ and $h_R = h_0$), and only scale the bandwidth of local FC (w_L) while maintaining its average power a constant ($S_L = S_0$). Like Supplementary Fig. 12, gain functions of different cases are illustrated in Supplementary Fig. 14 (a-d). Compared to 12, here we include one more different general short pulse CCS (14 (a)) in which the local bandwidth ($w_L = w'''_1$) is larger than readout bandwidth (w_0).

As shown clearly, the width of $G(\omega)$, total bandwidth w_G, decreases monotonically with the local bandwidth w_L.

Similarly, the area of $G(\omega)$, total gain S_G, also decreases monotonically with local bandwidth w_L, the trendline of which is shown in 14 (e).

The scaling of the height of $G(\omega)$, highest gain h_G, is slightly different. It gets maximized when $w_L = w_R$ (Symmetric CCS), as we derived before in Section 4.1.1. However, this metric is less important than the other two.

In short, as the local FC gets broader in frequency domain (shorter in time domain), the overall performance of CCS increased. This agrees with our general understanding that shorter pulses lead to larger peak power, which benefits efficiency of nonlinear process.

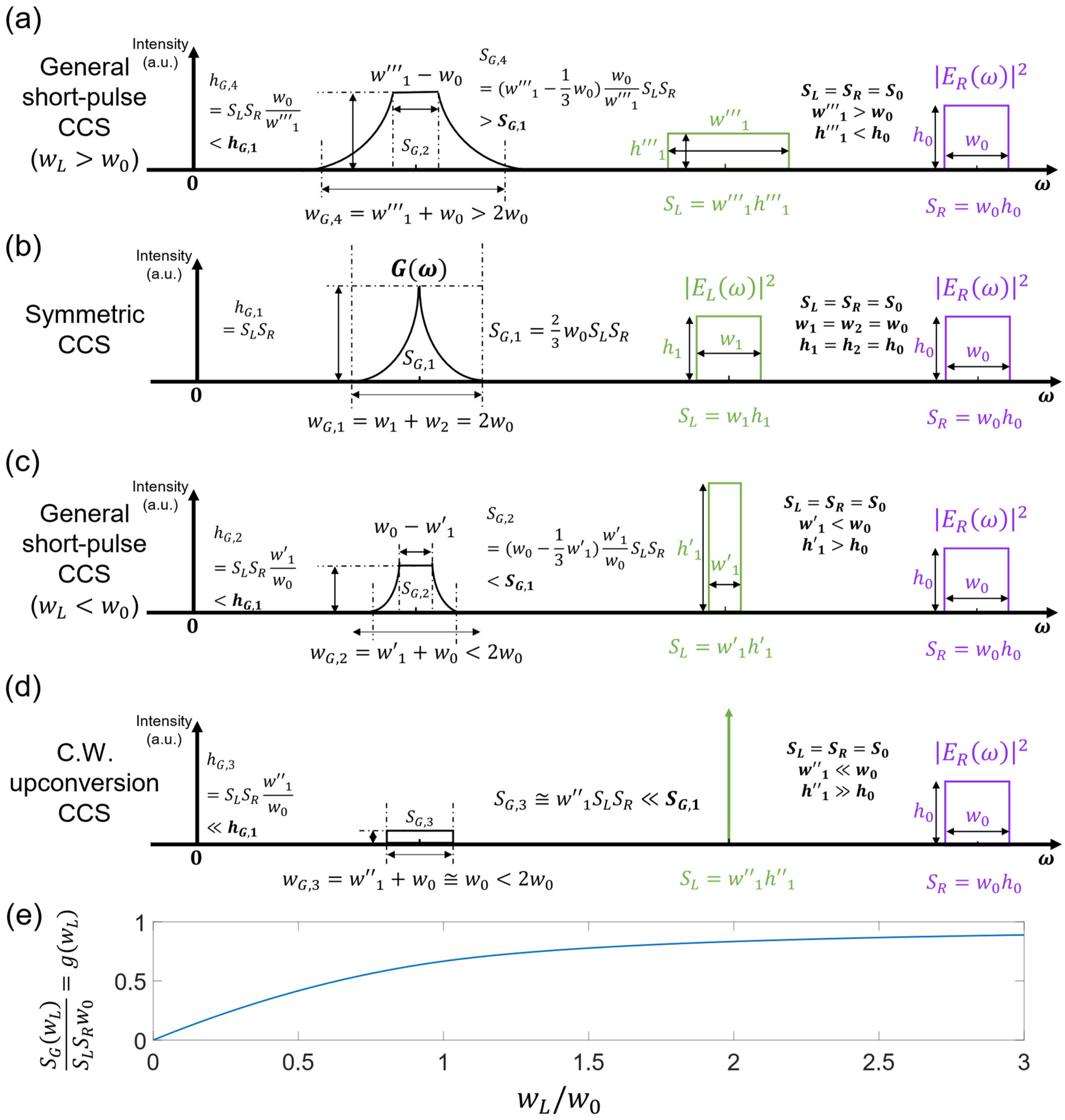

Supplementary Fig. 14| Gain function $G(\omega)$ of symmetric CCS (b), general CCS (a, c), and C.W. upconversion CCS (d), and S_G scaling with w_L (e), under a different assumption. In (d), the local FC spectral intensity profile is like a Dirac-delta function, which has a very small width w''_1 and a very large height h''_1, while their product S_L is kept same as the other three cases. In (e), note that the abscissa, local bandwidth w_L, is normalized by w_0, and the ordinate, total gain S_G, is normalized by $S_L S_R w_0$.

4.1.4 Summary

In this subsection we compare symmetric CCS, general short-pulse CCS, C.W. upconversion CCS, and EOS CCS. Compared to C.W. upconversion CCS, general short-pulse CCS can have a much higher detection (upconversion) efficiency, which comes from the enhancement of peak power of short pulses over C.W. laser. Compared to EOS CCS, general short-pulse CCS

(represented by symmetric CCS) can have a much larger detection bandwidth and detection efficiency, although it is much less experimentally demanding. Overall, among different upconversion configurations, short-pulse CCS has advantages in bandwidth, efficiency, flexibility, and experimental complexity.

4.2 Comparison between DCS and CCS: temporal gating, sensitivity, SNR, and dynamic range

4.2.1 Overview

In this part, we will provide a quick qualitative description. In asymmetric DCS, we have the correlation signal:

$$c(\tau) = \int_{-\infty}^{+\infty} e_T(t)e_R^*(t-\tau)dt \tag{64}$$

Note that only the cross term, i.e., the effective correlation signal, is kept in this equation. The background that is omitted in the equation is:

$$B = \int_{-\infty}^{+\infty} |e_T(t)|^2dt + \int_{-\infty}^{+\infty} |e_R^*(t-\tau)|^2dt = \int_{-\infty}^{+\infty} |e_T(t)|^2dt + \int_{-\infty}^{+\infty} |e_R^*(t)|^2dt \tag{65}$$

This background is equal to the sum of the full power of the target pulse and local pulse, which is independent of the delay, τ. At large delay τ, when the weak tail (optical free induction decay) of the target pulse is being sampled by the local pulse, the effective correlation signal can be much smaller than the constant background. In other words, the extra noise incurred by the background from the strong target pulse can envelop the weak useful signal at the tail. Even in the absence of technical noise, the strong background can saturate the detector, thus fundamentally limiting the dynamic range and SNR of the measurement[3].

In CCS, in which a short local pulse is used (not necessarily as short as in the EOS case), the correlation signal is:

$$c(\tau) = \int_{-\infty}^{+\infty} e_{SFG}(t,\tau)e_R^*(t-\tau)dt = \int_{-\infty}^{+\infty} e_T(t)e_L(t-\tau)e_R^*(t-\tau)dt \tag{66}$$

The omitted background terms are:

$$B(\tau) = \int_{-\infty}^{+\infty} |e_{SFG}(t,\tau)|^2dt + \int_{-\infty}^{+\infty} |e_R^*(t-\tau)|^2dt = \int_{-\infty}^{+\infty} |e_T(t)\boldsymbol{e_L(t-\tau)}|^2dt + \int_{-\infty}^{+\infty} |e_R^*(t)|^2dt \tag{67}$$

In stark contrast to DCS, the background in CCS is dependent on the delay τ, as the target pulse is “temporally gated” by a short local pulse $e_L(t-\tau)$. At the weak tail of the target pulse, where the effective correlation signal is weak, the background is also very weak, as it is free from the strong power of the center (peak) part of the target pulse. This allows a much stronger target pulse to be used, which promises a higher SNR at the weak tail, compared to the linear DCS. This behavior is well shown qualitatively in Fig. 1(e) of the main paper.

It is readily seen that the temporal gating effect is better as the local pulse is shorter. Also, a shorter local pulse benefits the upconversion efficiency. This is one of the reasons why we use a relatively short local pulse (broadband local FC) in our experiment, although only the total bandwidth of the local FC and readout FC is regulated in theory to fully map the target FC.

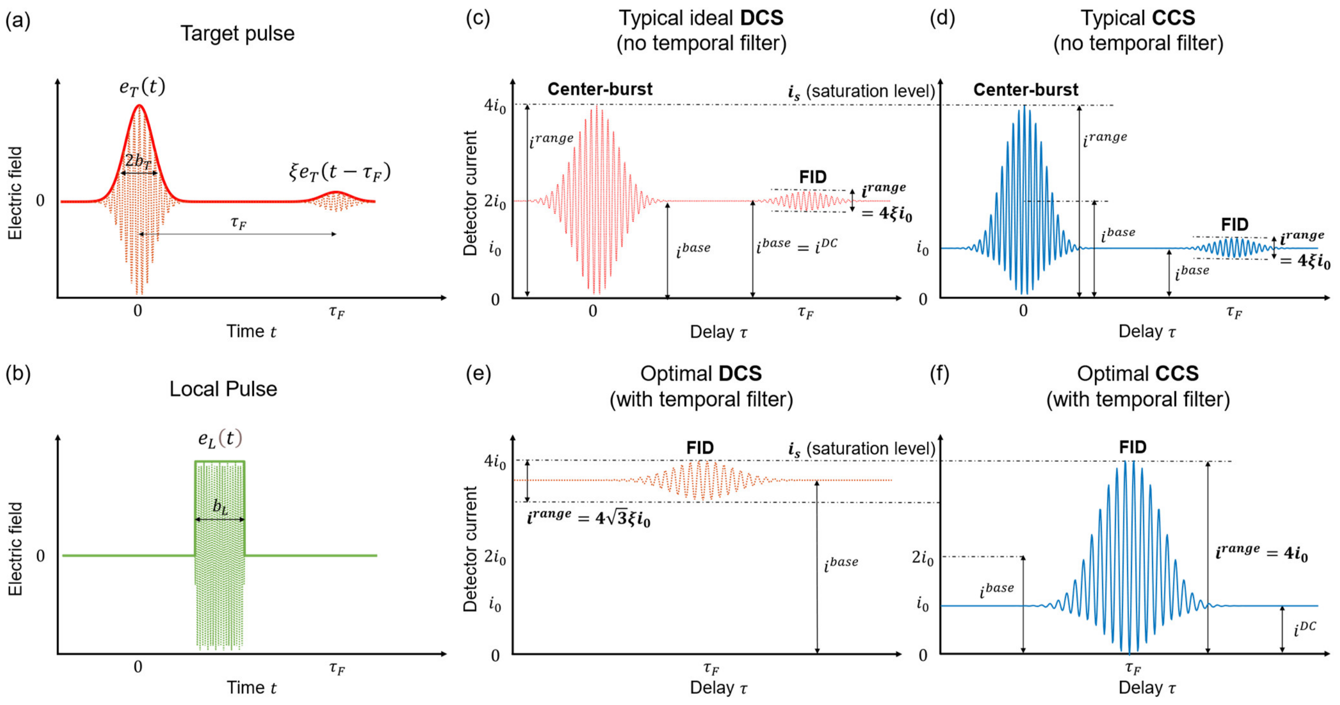

Supplementary Fig. 15| Theoretical model assumptions and comparison between CCS and DCS. a. Electric field of the target pulse $e_T(t)$, followed by its FID part $\xi e_T(t-\tau_F)$. **b.** Electric field of the local pulse $e_L(t)$. **c-d.** Typical interferograms of DCS **(c)** and CCS **(d)**, without temporal filtering. **e-f.** Optimal interferograms of DCS **(e)** and CCS **(f)** at FID, with a temporal filter to remove their center-bursts.

4.2.2 Assumptions of the model

To compare the SNR and sensitivity of DCS and short-pulse CCS quantitatively, firstly, we need to set up our model (Supplementary Fig. 15 (a)-(b)):

(1) When the target pulse does not go through sample, we describe it by: $e_T(t) = ag_b(t)\exp(i\omega_T t)$, where a_0 denotes the amplitude, $g_b(t)$ denotes the pulse envelope function. $g_b(t)$, and other functions $g(t)$ to follow, is set to be a gaussian function with a width of b , i.e., $\exp\left(-\left(\frac{t}{b}\right)^2\right)$. Also, we assume a slowly varying envelope, i.e., $\frac{dg(t)}{dt} \ll \omega$.

(2) After the target pulse goes through the sample, we describe it by $e_T^s(t) = \left[a_T g_{b_T}(t) + \xi a_T g_{b_T}(t-\tau_F)\right]\exp(i\omega_T t) = e_T(t) + \xi e_T(t-\tau_F)$ (see panel (a)). The first term denotes the original probing pulse (center-burst), and the second term denotes the FID from the sample. ξ denotes the amplitude ratio between the center and FID, which is $\ll 1$ if assuming a weak absorption measurement. τ_F denotes the time interval between the FID and the pulse center, and we assume it is $\gg b_T$ (the pulse width of $a_{\tau_T}(t)$), i.e., the FID signal is far enough from the center thus the field amplitude here is not influenced by the term $g_{b_T}(t)$. Indeed, it is not physically sound to assume the FID signal has the same pulse shape as the original excitation pulse. However, what matters for the following calculations is the amplitude ratio ξ between the center-burst and the FID signal, and these assumptions simplify the math without changing the core of the calculation. According to the derivation in part II of the supplementary material of ref. [3], ξ approximately equals the absorption up to a few other factors.

(3) For DCS, we set the readout pulse similar to the target: $e_R(t) = a_R g_{b_R}(t)\exp(i\omega_T t)$ (see $e_T(t)$ in panel (a)). For simplicity we assume $b_R = b_T$.

(4) For CCS, we set the local pulse as a square gating function, i.e., $e_L(t) = rect_{b_L}(t)$. b_L denotes the width of this square function, for which we assume $\tau_F \gg b_L > b_T$ (i.e., a local pulse width larger than target pulse width but much smaller than the interval between the FID and center-burst). Therefore, for the target pulse $e_T^s(t)$, around the center-burst, we

have $e_{SFG}(t,\tau \sim 0) = e_T^s(t)e_L(t-\tau) \cong e_T(t)$; around the FID, we have $e_{SFG}(t,\tau \sim \tau_F) = e_T^s(t)e_L(t-\tau) \cong \xi e_T(t-\tau_F)$. This assumption means the gating function effectively separates different temporal parts of the target pulse, and only the part that overlaps with the gating function can influence the detector value at a specific delay τ. As for the readout pulse, we assume it has the same envelope as the readout of DCS, although with a different optical frequency ω.

(5) For both DCS and CCS, we use a single slow detector that samples at a rate f_s, the same as the repetition rate of the readout FC ($f_s = f_{r,R} = f_{r,L} \cong f_{r,T}, T_s = \frac{1}{f_s} \ggg \tau_F \gg b$). Taking DCS as an example, at a delay τ, the detector current can be represented by: $i(\tau) = C_{ip}\int_{-\frac{T_S}{2}}^{\frac{T_S}{2}} |e_T(t) + e_R(t-\tau)|^2 dt$, where C_{ip} denotes a constant parameter that converts the result of the integration (equivalent to optical power) to photocurrent. Note that C_i includes some physical constants related to the electric field as well as the quantum efficiency and responsivity of the detector, which are not main subjects of this study. The parameter, C_{ip}, and integration limits, $\frac{T_S}{2}$ and $-\frac{T_S}{2}$, will be omitted to simplify the equation later, since the calculation is not generally sensitive to them.

(6) There are three kinds of noise that would be generally included in the SNR discussion: detector noise (NEP), shot noise, and relative intensity noise (RIN)[13]. For clarity, we do not include the RIN in this calculation. Therefore, unless the optical power is very low, shot noise is the main noise source here. Since we are going to apply the idea of "temporal gating", we study the SNR of the measurement in the time domain.

Let us first start with a typical ideal DCS measurement (no FID). We assume $e_T(t) = e_R(t) = ag_b(t)\exp(i\omega_T t)$, and $i_a = \int e_T^2(t)dt$. Please refer to Supplementary Fig. 15 (c) (the center-burst part) for illustration of the following discussion.

When $|\tau| \gg b$, i.e., the two pulses do not overlap, and we have a background signal:

$$i^{DC} = \int e_T^2(t)dt + \int e_R^2(t)dt = 2i_a \tag{68}$$

At $\tau = 0$, when the two pulses constructive interfere, i.e., the maxima of the interferogram, we have:

$$i^+(0) = \int \left(2e_T(t)\right)^2 dt = 4i_a \tag{69}$$

At $\tau \cong 0$, when the two pules destructively interfere, i.e., the minimum of the interferogram, we have:

$$i^-(0) \cong \int [e_T(t) - e_R(t)]^2 dt = 0 \tag{70}$$

Thus, the range of the interference here, denoted by i^{range}, is $4i_a$, which can be understand as the amplitude of the "signal". To evaluate the noise, we define the base current i^{base} around $\tau = 0$ as the average value of $i^+(0)$ and $i^-(0)$, which is:

$$i^{base}(0) = \frac{1}{2}(i^+(0) + i^-(0)) = 2i_a \tag{71}$$

In this case, the base current around the maxima is equal to the background i_{DC}. Since shot noise here increases with i_b, to optimize SNR, one wants to optimize the ratio i^{range}/i^{base}. Actually, this ratio is equivalent to the "interferometric visibility" up to a factor of $1/2$.

The shot noise around $\tau = 0$ can be expressed by:

$$i^{sn} = C_{isn}\sqrt{i^{base}} = C_{isn}\sqrt{2i_a} \tag{72}$$

C_{isn} denote a constant parameter that convert $\sqrt{i^{base}}$ into current noise. Like C_{ip}, C_{isn} includes some physical constants which are not the main subjects of this study.

If the optical power is low, the dominant noise is detector noise, and the SNR of the measurement is:

$$SNR = \frac{i^{range}(0)}{i_{NEP}} = \frac{4i_a}{i_{NEP}} \tag{73}$$

The dominant noise becomes the shot noise when the optical power is higher, and SNR of the measurement is:

$$SNR = \frac{i^{range}(0)}{i^{sn}} = \frac{4i_a}{C_{isn}\sqrt{2i_a}} \tag{74}$$

Apparently, the SNR increases with $\sqrt{i_a}$, and this dependence agrees with general shot-noise-limited measurement since $i_a \propto$ the power of the target (or readout) pulse. Also, the above two equations are equivalent to equation (24) and (25) of ref [13], although different letter conventions are used, and the RIN and dynamic range terms are not included.

The SNR can be improved by increasing i_a (average power of FCs), which stops when $i^+(0) = 4i_a$ reaches i_s, i.e., the saturation level of the detector. Let $i_0 = \frac{1}{4}i_S$, thus the SNR reaches maxima when $i_a = i_0$ ($4i_0 = i_s$):

$$SNR_{DCS,max} = \frac{4i_0}{C_{isn}\sqrt{2i_0}} \tag{75}$$

4.2.3 SNR at FID, without temporal filtering

To compare the sensitivity for weak absorption, we need to calculate SNR at the FID instead of the center-burst. Let us start with DCS following the last subsection. This time we assume $e_T^s(t) = [a_0 g_b(t) + \xi a_0 g_b(t-\tau_F)]\exp(i\omega_T t) = e_T(t) + \xi e_T(t-\tau_F), e_R(t) = e_T(t) = a_0 g_b(t)\exp(i\omega_T t), i_0 = \int e_R^2(t)dt = \int e_T^2(t)dt.$ See Supplementary Fig. 15 (c).

At the center-burst, $i^+(0) = 4i_0 = i_S$, and the SNR here reaches maxima, as explained above.

However, at the FID, everything changes. We have:

$$i^+(\tau_F) = \int \left((1+\xi)e_T(t)\right)^2 dt + \int e_T^2(t)dt = [(1+2\xi+\xi^2)+1]i_0 \tag{76}$$

$$i^-(\tau_F) = \int \left((1-\xi)e_T(t)\right)^2 dt + \int e_T^2(t)dt = [(1-2\xi+\xi^2)+1]i_0 \tag{77}$$

$$i^{range}(\tau_F) = i^+(\tau_F) - i^-(\tau_F) = 4\xi i_0 \tag{78}$$

$$i^{base} = \frac{1}{2}[i^+(\tau_F) + i^-(\tau_F)] = (2+\xi^2)i_0 \cong 2i_0 \tag{79}$$

$$SNR_{DCS,FID} = \frac{4\xi i_0}{C_{isn}\sqrt{2i_0}} = \xi SNR_{DCS,max} \tag{80}$$

In short, although the amplitude of the signal (the interference) is ξ times weaker than that at the center-burst, the noise level is still the same since the detector can see all the background from the center-burst, i.e., the large energy which contributes to only the noise here but not the signal.

Let us then consider CCS. (See Supplementary Fig. 15 (d).) At the center-burst, since we assume $e_{SFG}(t,\tau \sim 0) = e_T'(t)e_L(t-\tau) \cong e_T(t)$, its SNR has the same results as DCS. However, at the FID, $e_{SFG} \cong \xi e_T(t-\tau_F)$, we have:

$$i^+(\tau_F) = \int \left((1+\xi)e_R(t)\right)^2 dt = [(1+2\xi+\xi^2)]i_0;\; i^-(\tau_F) = \int \left((1-\xi)e_R(t)\right)^2 dt = [(1-2\xi+\xi^2)]i_0 \tag{81}$$

$$i^{range}(\tau_F) = 4\xi i_0;\; i^{base} \cong i_0 \tag{82}$$

$$SNR_{CCS,FID} = \frac{4\xi i_0}{C_{isn}\sqrt{i_0}} = \sqrt{2}\, SNR_{DCS,FID} \tag{83}$$

This shows a $\sqrt{2}$ SNR enhancement of CCS over DCS, which is not significant. In fact, here we greatly limit the capability of CCS. On one hand, in this simple model we assume the same detector performance (NEP, responsibility, quantum efficiency, saturation level, and etc.) of the NIR detector of CCS and MIR detector of DCS, the latter of which should be worse the former. On the other hand, we still limit the optical power, especially the local pulse, of the CCS, to avoid detector saturation at the center-burst, which is not necessary for detecting weak absorption.

4.2.4 SNR at FID, with temporal filtering

Now we introduce a temporal filter that throws out the interference signal at the center-burst (before the FID) and only keep the interference around FID for the detection of weak absorption[3]. In this case, we no longer care about the detector saturation at the center-burst; the limits of the SNR at the FID are set by the detector saturation at the FID locally.

For DCS, we can increase both the power of $e_R(t)$ and $e_T(t)$ by 2 times, i.e., $e_R(t) = \sqrt{2}e_T(t), e_T^s(t) = \sqrt{2}e_T(t) + \sqrt{2}\xi e_T(t-\tau_F)$, to maximize the SNR here. See Supplementary Fig. 15 (e). We cannot increase more since $i^+(\tau_F)$ already saturates the detector. We have:

$$i^+(\tau_F) = \int \left(\left(\sqrt{2}+\sqrt{2}\xi\right)e_T(t)\right)^2 dt + \int 2e_T^2(t)dt = [(2+4\xi+2\xi^2)+2]i_0 \cong 4i_0 = i_s \tag{84}$$

$$i^-(\tau_F) = \int \left(\left(\sqrt{2}-\sqrt{2}\xi\right)e_T(t)\right)^2 dt + \int 2e_T^2(t)dt = [(2-4\xi+2\xi^2)+2]i_0 \cong 4i_0 = i_s \tag{85}$$

$$i^{range}(\tau_F) = i^+(\tau_F) - i^-(\tau_F) = 8\xi i_0;\ \ i^{base}(\tau_F) = \frac{1}{2}[i^+(\tau_F) + i^-(\tau_F)] = (4+2\xi^2)i_0 \cong 4i_0 \tag{86}$$

$$SNR_{DCS,FID}^{gated} = \frac{8\xi i_0}{C_{isn}\sqrt{4i_0}} = \sqrt{2}\,SNR_{DCS,FID} = \sqrt{2}\xi SNR_{DCS,max} \tag{87}$$

Compared to not-gated case, the SNR can only be increased by a factor of $\sqrt{2}$.

For CCS, thanks to temporal gating by the local pulse, the detector signal at the FID is free from the power of the center part of the target pulse (the second term of the RHS of the above equation of $i^+(\tau_F)$, i.e., $\int 2e_T^2(t)dt$). Thus, we can increase the power of the local pulse by a factor of $(\frac{1}{\xi})^2$, i.e., $e_L'(t) = \frac{1}{\xi}rect_{b_L}(t)$. Then, at the FID, (See Supplementary Fig. 15 (f))

$$e_{SFG}(t,\tau=\tau_F) = \xi e_T(t-\tau_F)e_L'(t-\tau_F) = \xi e_T(t-\tau_F)\frac{1}{\xi}rect_{b_L}(t-\tau_F) \cong e_T(t-\tau_F) \tag{88}$$

If we keep $e_R(t) = e_T(t)$, we will have an SNR equivalent to that of the center-burst of a typical ideal DCS or CCS measurement, calculated in section 4.2.2:

$$i^+(\tau_F) = \int\left(2e_T(t-\tau_F)\right)^2 dt = 4i_0 = i_s;\ i^-(\tau_F) = 0; i^{range}(\tau_F) = 4i_0;\ i^{base}(\tau_F) = 2i_0 \tag{89}$$

$$SNR_{CCS,FID}^{gated} = \frac{4i_0}{C_{isn}\sqrt{2i_0}} = \frac{\mathbf{1}}{\sqrt{\mathbf{2}\boldsymbol{\xi}}}SNR_{DCS,FID}^{gated} \tag{90}$$

Therefore, a $\frac{1}{\sqrt{2}\xi}$ SNR enhancement is demonstrated for CCS compared to DCS at FID, assuming sufficient upconversion. The weaker the absorption, the stronger the enhancement can be. The reason for this lies in the fact that the DCS signal at the FID always comes with a factor of $1/\xi$ stronger DC background which contributes only to the noise but not the signal, while CCS does not. This comparison is illustrated in Fig. 2d of the main paper.

In practice, one can "infinitely" decreases ξ by decreasing the sample concentration or gas cell length. However, the enhancement ratio $\frac{1}{\sqrt{2}\xi}$ cannot be infinitely increased; the limit is set by two factors, whichever comes first:

1. The SFG efficiency. To fully reach the SNR enhancement, one needs to upconvert the target pulse by a factor $1/\xi$ stronger using local pulses with a higher peak power. However, this can be clamped by the highest available peak power of local pulses or the damage threshold of the SFG crystal.
2. The damage threshold of the NIR detector. Although the detector saturation at the center-burst no longer matters if we discard the signal there, we still do not want the power there to damage the detector. Generally, however, the detector is damaged by the average power rather than the peak power, and the average power on the detector mainly depends on that of the readout pulses instead of the local pulses. In other words, just a very small portion of the local pulse average power contributes to the total average power on the detector, the ratio of which is decided by the "duty cycle" of the SFG process (see Section 1.3). In temporal-filtered CCS, the strategy is to use stronger local pulses while keeping the readout pulses unchanged, which adds only a tiny optical average power on the detector. Hence, it is very unlikely that the limit of this factor comes earlier than the former.

4.2.5 Sensitivity and dynamic range, with temporal filtering

Following the SNR calculation, a comparison of sensitivity (the minimal detectable ξ) becomes straightforward. Let us define ξ_{min}, the minimal detectable ξ that makes SNR=1. For DCS at the FID, we have:

$$SNR_{DCS,FID}^{gated} = \frac{8\xi_{min}^{DCS} i_0}{C_{isn}\sqrt{4i_0}} = 1 \tag{91}$$

$$\xi_{min}^{DCS} = \frac{C_{isn}}{2\sqrt{i_s}} \tag{92}$$

Clearly it is limited by i_s, the detector saturation. ($i_s = 4i_0$)

For CCS, the sensitivity depends entirely on the upconversion capability, as discussed before. Let us assume the upconversion conversion ratio is $1/\xi_0$. Then for an absorption ξ_0, as derived before, we have an SNR $= \frac{4i_0}{C_{isn}\sqrt{2i_0}}$ at the FID if we set $e_R(t) = e_T(t)$. However, this SNR is much larger than 1 and more than enough to detect the absorption signal. Thus, we can further decrease the absorption. Let us assume an extra absorption factor ξ_1 to make the target FID a "small signal". Simultaneously, to maximize SNR, we set $e_R(t) \cong 4\, e_T(t)$. Then, at the FID, we have:

$$i^{range}(\tau_F) = 16\xi_1 i_0;\ i^{base}(\tau_F) = 4i_0;$$

$$SNR_{CCS,FID} = \frac{16\xi_1 i_0}{C_{isn}\sqrt{4i_0}} \tag{93}$$

Assuming this SNR=1, we have:

$$\xi_{1,min} = \frac{C_{isn}}{4\sqrt{i_s}} = \frac{1}{2}\xi_{min}^{DCS} \tag{94}$$

Adding the ratio ξ_0 back, we have:

$$\xi_{min}^{CCS} = \xi_0\xi_{1,min} = \frac{1}{2}\xi_0\xi_{min}^{DCS} \tag{95}$$

Therefore, CCS can detect a $\frac{1}{2}\xi_0$ smaller absorption compared to DCS. The detection is limited by the upconversion capability rather than the detector.

It should be noted that, in order to maximize the sensitivity (minimize the detectable ξ) for both techniques, we set their parameters ($e_R(t)$ and $e_T(t)$) to make i_{base} close to detector saturation. However, in such settings, there will not be dynamic range for the detection: a larger absorption will excess the saturation limit, and lower absorption will make $SNR < 1$ thus not detectable. In short, for a detection where we want to get good dynamic range, we do not want to use the settings for the best sensitivity.

We continue to compare their dynamic ranges. In the interest of fairness, we used different power settings for each method to optimize their respective DR while keeping their sensitivity (minimal detectable absorption, the lower limit of the detectable range) the same. The comparison is illustrated in Fig. 2e.

In CCS, for an arbitrary low absorption ξ_{low}, we keep powers of readout FC ($e_R(t)$) and SFG signal ($e_{SFG}(t,\tau_F)$) the same and to be a quarter of the detector NEP. Note that SFG power can be tuned by either target power ($e_T(t)$) or local power ($e_L(t)$). In such power setting, the interference range (i^{range}) would be equal to the i_{NEP}, making the absorption just detectable. Note that the dominant noise source in this scenario is detector noise (NEP) rather than the shot noise as readout power and SFG power are both tuned very low. Any stronger absorption would make $e_{SFG}(t,\tau_F)$ larger and more detectable until the detector is saturated. In other words, the interference range can vary from i_{NEP} to i_S, which means the DR of the absorption (ξ) can utilize the full DR of the detector $\left(\frac{i_S}{i_{NEP}}\right)$ and we have $DR_{CCS_\xi} = \sqrt{DR_{detector}}$. The square root here is simply because we use ξ, a ratio of optical field, to quantify absorption, but detector current is proportional to optical power (square of the field).

In DCS, if we keep the readout power and target power here as same as those of the CCS case, we will get a same interference range $i^{range} = i_{NEP}$, which makes the same small absorption ξ_{low} detectable. However, unlike CCS, a large extra DC background will always be seen by the detector due to a lack of temporal gating. This power can occupy a large portion of the detector DR and therefore limits the range of the detectable absorption. Since this extra background is a factor of $\left(\frac{1}{\xi_{low}}\right)^2$ larger than the interference background in CCS, the dynamic range of DCS would be about a factor of ξ_{low} smaller than that of CCS, i.e., $DR_{DCS_\xi} = \sqrt{(\xi_{low})^2 DR_{detector}} = \xi_{low}\, DR_{CCS_\xi}$. In fact, this still underestimates the difference between them, since in DCS the dominate noise would become the shot noise at the same ξ_{low} because of the large background. Therefore, even larger target power or readout power has to be used to make the same absorption detectable, i.e., make the interference larger than the total noise, which is now the sum of shot noise and detector noise. This explains why for the same ξ_{low}, in Fig. 2e of the main paper, the interference of the DCS looks larger than that of the CCS; it has to be made larger by a larger target or readout power, to overcome a larger noise. This larger target or readout power will then occupy more $DR_{detector}$. Moreover, the lower the ξ_{low} set to be, the more $DR_{detector}$ has to be occupied as higher target or readout power has to be used in DCS to make the signal detectable, and larger the difference between DCS and CCS will be.

4.2.6 SNR scaling with power of different combs

Here we provide a direct analysis about how temporal SNR of the interferogram scales with powers of different combs, based on the same model and assumptions.

First of all, as the SFG process in our experiment and theoretical model is far from saturation or depletion, the SFG output power is expected to be linear to target power or local power, which is consistent with what we observed in the experiment.

Then the question becomes how SNR scales with the SFG power, which depends on the relative power of the other arm of the interference, readout power, and the current dominant noise source of the detection. This is equivalent to how a typical interference (between two short pulses) SNR scales with the power of one arm. We can use a simple equation to explain this:

$$I = \left(\sqrt{P_{SFG}} + \sqrt{P_r}\right)^2 = P_{SFG} + P_r + 2\sqrt{P_{SFG}P_r} \quad (96)$$

with which we have,

$$signal \propto \sqrt{P_{SFG}P_r} \quad (97)$$

$$Noise = (shot\ noise + NEP) \propto \left(\sqrt{P_{SFG} + P_r} + P_{NEP}\right) \quad (98)$$

Then there are a few different cases:

a. When SFG and readout power are both low and the dominant noise is the detector noise, the SNR scales linearly with the square root of SFG power or target/local power ($S \propto \sqrt{P_{SFG}}, N = P_{NEP}$).

b. When SFG power is high, readout power is low, and the dominant noise is the shot noise from SFG power, the SNR does not change with the SFG power or target/local power ($S \propto \sqrt{P_{SFG}}, N \propto \sqrt{P_{SFG}}$).
c. When SFG power is low, readout power is high, and the dominant noise is the noise from readout power, the SNR scales linearly with the square root of the SFG power or target/local power ($S \propto \sqrt{P_{SFG}}, N \propto \sqrt{P_r}$).
d. When both the SFG power and readout power are high and comparable, and the dominant noise is shot noise from both the SFG power and readout power, the SNR increases with the SFG power or target/local power ($S \propto \sqrt{P_{SFG}}, N \propto (\sqrt{P_{SFG} + P_r})$).

Note that in above analyses, the roles of SFG and readout FC are equivalent, and the roles of target power and local power are also equivalent. Those analyses are basically equivalent to that of typical optical heterodyne detection.

4.2.7 Summary

The discussion above demonstrates the fact that the sensitivity of short-pulse CCS is limited by upconversion capability (SFG efficiency), which is fundamentally different from DCS, where it is limited by the detector saturation. The beauty of the short-pulse-upconversion CCS is that strong local pulses can greatly enhance the peak power of the signal of interest in a localized temporal window, with minimal increase to the background signal and average power on the detector which add to noise and saturation of the detector. This time gating effect endows CCS advantages in SNR, sensitivity, and dynamic range.

Moreover, it should be noted that, among the three different upconversion configurations, only short-pulse-upconversion CCS can fully have these advantages. Firstly, C.W. upconversion CCS is basically DCS, which does not have advantages we discussed here at all. Secondly, for EOS CCS, one may expect it to have the same advantages since it also uses short pulses for upconversion, but this is not true if no more efforts are taken to independently control the power and spectrum of different spectral parts of the ultrashort pulses. Admittedly, the even higher peak power of the local pulse (because of shorter pulse length) used in EOS CCS can provide even higher upconversion efficiency. However, when the average power of the local pulses is increased to detect weaker absorption, that of the readout part of the local spectrum is also increased, which can saturate the detector unexpectedly, if they are not independently controlled. In other words, in short-pulse-upconversion CCS, you can always use higher local power to amplify a weaker FID signal while keeping the readout power unchanged, and you will never saturate the detector. However, in EOS CCS, you cannot do the same since local and readout are from the same pulse (spectrum), and thus their power cannot be tuned independently.

5. **Comparison of performance between different techniques, by simulation**

In previous sections we have compared principles and some performance metrics of different techniques using simplified theoretical models. In this section, we will provide a more quantified comparison by numerical simulation, to further demonstrate the advantages of CCS. We will focus on the SNR and sensitivity of the typical MIR DCS, MIR CCS without temporal filtering, and MIR CCS with temporal filtering, and how they scale with the sample concentration (absorbance).

5.1 Simulation assumptions

Although more quantified and accurate, the assumptions of our simulations are still basically consistent with those of the previous theoretical models. These assumptions will be explained in detail below.

5.1.1 Frequency combs

We use femtosecond pulses for both DCS and CCS. For DCS, transform-limited sech^2 pulses of a FWHM of 250 fs centered at 4270 nm (70 THz) are used as the target FC, and the same pulse profile is used for the readout FC. For CCS, the exact same target FC is used, and pulses with the same shape and width but centered at 1560 nm (192 THz) and 1145 nm (262 THz) are used as the local FC and readout FC, respectively. Their repetition rates are set at 250 MHz, and the difference in repetition rates is set at 1kHz. The detection bandwidth is taken to be 125 MHz, i.e., half of the repetition rate.

For DCS, the comb powers are assumed to be enough to saturate the MIR detector (~1 mW), which is practical as many high-power MIR combs have been demonstrated in the past decade. For CCS, we also assume enough MIR power, local power, and nonlinearity, which is interchangeable with higher SFG power, to have a high enough upconverted SFG power to saturate the NIR detector (also ~1mW). This assumption is also practical considering our experimental results, recent progress in related areas, and state-of-the-art techniques as discussed in the main paper.

5.1.2 Sample

Here we use 1-meter-long CO_2 of ambient level (~400 ppm) as the sample of unit concentration (relative concentration 10^0 in Supplementary Fig. 22), which is used in simulation for different techniques.

We model the CO_2 response using the Lorentz oscillator model[14] in which the refractive index as a function of frequency, $n(\omega)$, is given by the equation

$$n^2(\omega) = 1 + \sum_{ij} \frac{f_{ij} N_j q^2}{2\varepsilon_0 m_e\left(\omega_{ij}^2 - \omega^2 + i\gamma_{ij}\omega\right)} \tag{99}$$

where the indices i, j refer to the upper and lower states of the transitions of interest, N_j is the density of molecules in state j, f_{ij} is the oscillator strength, q is the electron charge, m_e is the electron mass, ε_0 is the vacuum permittivity, ω_{ij} is the angular frequency of the transition's line center, and γ_{ij} is the transition linewidth. Line parameters for the CO_2 transitions are taken from the HITRAN database[15]. The absorption and dispersion can be directly computed from the resulting complex refractive index through the relationship

$$n(\omega) = n'(\omega) - i\kappa(\omega) \tag{100}$$

where the dispersion information is contained in $n'(\omega)$ and the absorption profile is given by $\kappa(\omega)$.

Note that although we use target pulses centered at 4270 nm and CO_2 absorption, this simulation can be adapted to other MIR wavelength easily, which would not fundamentally change the conclusions of this section.

5.1.3 Detectors

Detectors are an important factor in considering the differences between MIR DCS and CCS. We choose an InGaAs detector as NIR detector and a HgCdTe (MCT) detector as MIR detector, each of which is a very typical choice in its wavelength region. While different detectors from different manufacturers can have very different performance metrics, we adapt the specifications of two commercial detectors from Thorlabs, FPD510-FS-NIR (InGaAs) and PDAVJ10 (MCT), for the simulation, which can well represent the general metrics of these two kinds of detectors. Note that we also refer to a review paper[16] for D^* of these detectors.

For the NIR InGaAs detector, we assume:

Spectral responsivity 0.8 A/W; Size $(1\ mm)^2$; D*=$1\times10^{11}\ cm*Hz^{\frac{1}{2}}*W^{-1}$; NEP= $1\ pW/Hz^{\frac{1}{2}}$; $P_{saturation}=1\ mW$

For the MIR MCT detector (at room temperature), we assume

Spectral responsivity $0.01\ A/W$; Size $(1\ mm)^2$; D*=$5\times10^{8}\ cm*Hz^{\frac{1}{2}}*W^{-1}$; NEP= $200\ pW/Hz^{\frac{1}{2}}$; $P_{saturation}=1\ mW$

5.1.4 Noise

As before, we include detector noise and shot noise in our simulation. For the assumed NIR detector, the detector noise will dominate at low input powers, while shot noise will dominate at higher input powers. For the assumed MIR detector, however, even at detector saturation, the shot noise will be about the same order of magnitude as the detector noise, as shown by the simulation below, because of its low responsivity and high NEP. Therefore, the shot noise and detector noise are both important at high input powers for the MIR detector. Note that this detector difference is not included in our theoretical model above, making that model effectively less advantageous to CCS.

5.1.5 Nonlinearity

For simplicity, we assume an ideal nonlinear conversion process where the upconverted field is the product of the target and local field (the SFG part), which is consistent with the theoretical model. The power efficiency is estimated by the standard SFG model with the assumption of quasi-C.W. operation[17,18]. The nonlinear crystal is assumed to be lithium niobate. Although a more accurate model for the nonlinearity could be used to give a more accurate estimation of upconversion efficiency and bandwidth, these estimates will not affect most parts of this simulation, because generally there will be enough SFG power to saturate the detector. The accurate estimation of upconversion efficiency will only be important to estimate the limits of the CCS with temporal filtering, as we will explain later.

5.2 DCS

With all the parameters assumed, the DCS interferograms can be simulated, as presented in Supplementary Fig. 16. The average power of the target FC and local FC are both set to one quarter of the $P_{saturation}$, by which the detector will be just saturated (10 μA) at the center peak of the reference interferogram (see panel a). The reference interferogram (without sample) and absorbed interferogram (with sample) are present in upper panels (a-d) and lower panels (e-f), respectively. As the full interferograms are too long to show, we present the center-burst ((a) and (e)) and one typical part of the FID ((b) and (f)), which can exhibit key signatures of the sample. In (a) and (b) ((e) and (f)), we show only the ideal unbalanced interferogram (signal from one arm of the balanced detector), to better display the relationship between the baseline (D.C.) and the effective interference (A.C.). The corresponding balanced interferograms, as well as noises, are shown in panel (c) and (d) ((g) and (h)). Note that it is because we use a relatively strong absorption here that the effective interference at the FID is fairly observable compared to the background (see (f)) and the noises (see (h)), but the signal could be easily overwhelmed by the noise if a much lower absorption were considered.

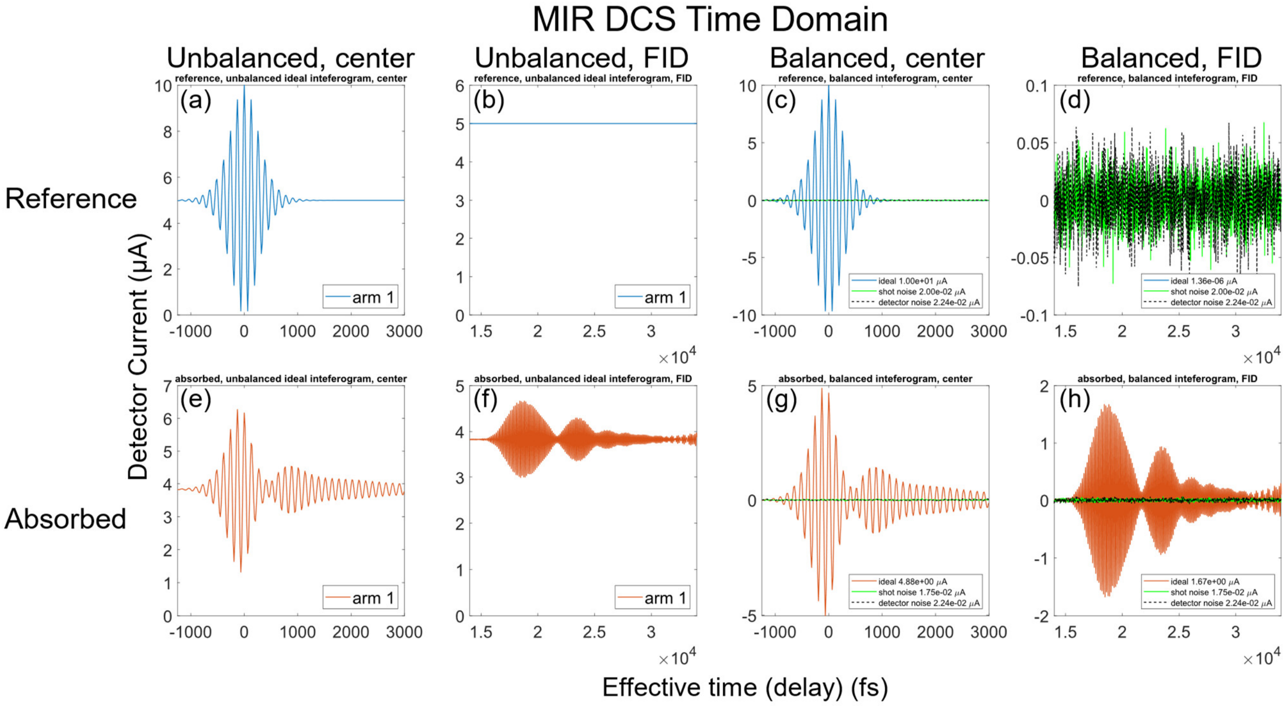

Supplementary Fig. 16| Time domain of MIR DCS. a. Unbalanced ideal interferogram at the center (-1.25 ps – 3 ps) of the reference measurement (without sample). Note that only the signal recorded by one arm of the balanced detector is shown, and signal at the other arm is of π-phase difference, which is also the case for (b), (e), and (f). **b.** Unbalanced ideal interferogram at one part of the FID (-14 ps – 34 ps) of the reference measurement. **c.** Balanced ideal interferogram at the center of the reference measurement, together with noise. The maxima of the ideal interferogram, as well as the average level (standard deviation) of the noise, is denoted in the legend box for the reference; the same is done in (d), (g) and (h). **d.** Balanced ideal interferogram at one part of the FID of the reference measurement, together with noises. **e.** Unbalanced ideal interferogram at the center of the absorbed measurement (with sample). **f.** Unbalanced ideal interferogram at one part of the FID of the absorbed measurement. **g.** Balanced ideal interferogram at the center of the absorbed measurement, together with noises. **h.** Balanced ideal interferogram at one part of the FID of the absorbed measurement, together with noises.

By taking the Fourier transform of those temporal signals and noises, spectra can be obtained, which are shown in Supplementary Fig. 17. In panel (a), the spectral amplitude of the ideal reference measurement, the ideal absorbed measurement, and the total noise (sum of the two kinds of noises) are presented, and their average amplitudes are displayed in the legend box. The spectra are truncated to an interval with endpoints where the reference amplitude equals the noise amplitude. Note that the frequency axes are obtained from the direct Fourier transform, which needs to be linearly mapped to real frequencies. However, it is not done in the figures as it is not necessary for our purpose. The spectra of the two noises are presented individually in panel (b). As mentioned before, for this MIR detector, the detector noise is still close to the shot noise even when the detector is saturated in reference measurement, so both are important.

The real "signal", corresponding to "noise", in the absorption measurement, is neither the reference spectrum nor the absorbed spectrum, but the difference between them. The difference spectrum is depicted in panel (c), together with spectra of the reference and the noise. The SNR of the measurement, defined as a spectral average, is the ratio of the average amplitude of the difference spectrum to that of the noise spectrum. The SNR increases with the absorption (sample concentration) as the difference amplitude increases with the absorption and approaches its upper limit. The upper limit, i.e., the max SNR, is the ratio of the average amplitude of the reference spectrum to that of the noise spectrum, because difference amplitude cannot be larger than the reference amplitude. In other words, the difference spectrum approaches the reference spectrum when the absorption is very large, while the SNR approaches its maximum. On the other side, lower absorption would result in a smaller

difference amplitude and thus lower SNR, which can approach zero. Note that the SNR here is defined differently with that of ref [13], however, the max SNR defined here is fundamentally equivalent to the SNR (figure of merit) there.

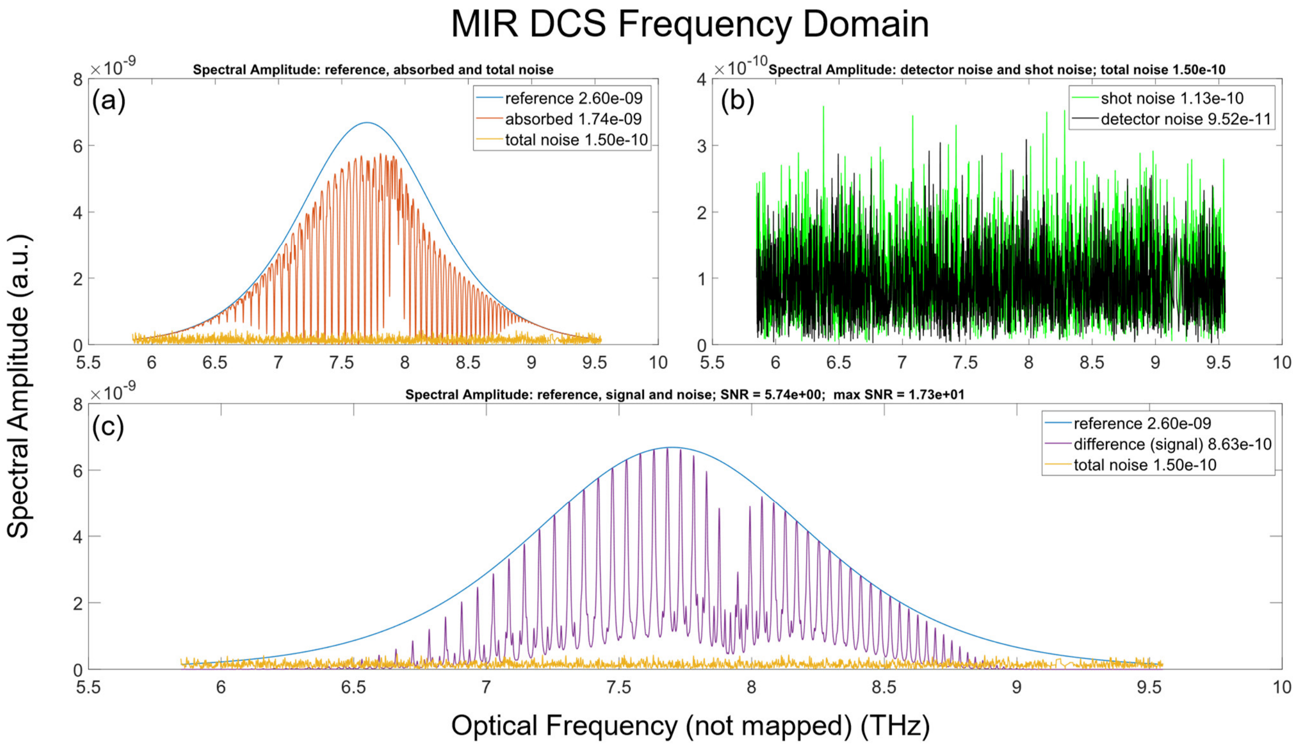

Supplementary Fig. 17| Frequency domain of MIR DCS. a. Spectral amplitudes of the ideal reference measurement, ideal absorbed measurement, and total noise. The spectral amplitudes are actually complex values with phase information, but we show only their magnitude in our figures for clarity. The average amplitudes of each spectrum are displayed in the legend box; the same is done in (b) and (c). **b.** Spectral amplitudes of the shot noise and detector noise. **c.** Spectral amplitudes of the ideal reference measurement, ideal difference spectrum, and total noise. The spectral average SNR at this (unit) sample concentration (5.74) and its upper limit (17.3) are displayed in the plot subtitle.

5.3 CCS (without temporal filtering)

As with DCS described above, the time domain and frequency domain results of CCS are presented in Supplementary Figs. 18 and 19, respectively. The SFG pulse power at zero-time-delay and the readout power are set to be the same, equal to one quarter of the $P_{saturation}$, meaning the detector will be just saturated (800 μA) at the center peak of the reference interferogram (see 18 panel a). For this NIR detector, at saturation, the dominant noise is shot noise because of its high responsivity and low detector noise (19 panel b). Moreover, although we set the same relative powers here as for the previous DCS case (one quarter of detector saturation), the relative D.C. baseline here (one quarter of the saturation level) is lower than that of the previous case (half of the saturation level), which leads to less relative shot noise. This is the result of temporal gating, which agrees with our discussion before in the theoretical model (Supplementary Section 4.2.3). A spectral average SNR of 46.8 at unit concentration and its upper limit of 161 are measured, as shown in 19 panel c. The SNR would scale with sample concentration in the same way as that of the DCS above, due to the nature of the interference and measurement.

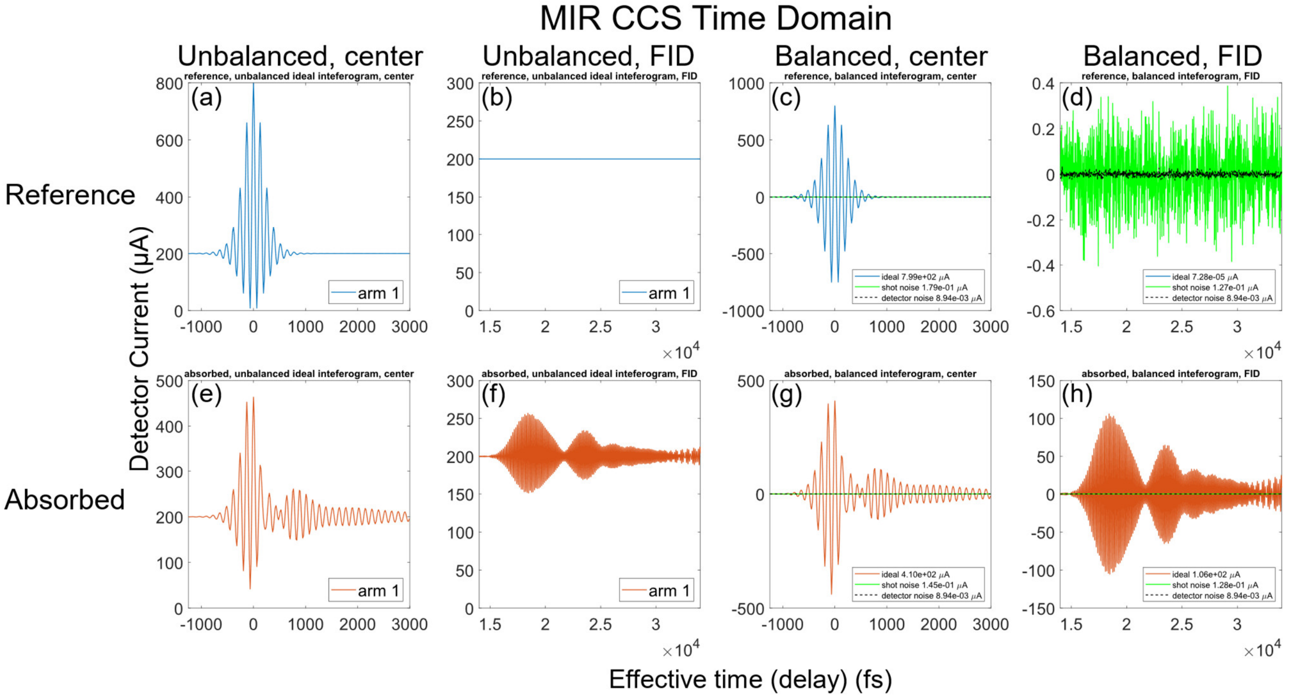

Supplementary Fig. 18| Time domain of MIR CCS. a. Unbalanced ideal interferogram at the center (-1.25 ps – 3 ps) of the reference measurement (without sample). Note that only the signal recorded by one arm of the balanced detector is shown, and signal at the other arm is of π-phase difference; the same is true for (b), (e), and (f). **b.** Unbalanced ideal interferogram at one part of FID (14 ps – 34 ps) of the reference measurement. **c.** Balanced ideal interferogram at the center of the reference measurement, together with noises. The maxima of the ideal interferogram, as well as the average level (standard deviation) of noises, is denoted in the legend box for reference; the same is true in (d), (g) and (h). **d.** Balanced ideal interferogram at one part of FID of the reference measurement, together with noises. **e.** Unbalanced ideal interferogram at the center of the absorbed measurement (with sample). **f.** Unbalanced ideal interferogram at one part of FID of the absorbed measurement. **g.** Balanced ideal interferogram at the center of the absorbed measurement, together with noises. **h.** Balanced ideal interferogram at one part of FID of the absorbed measurement, together with noises.

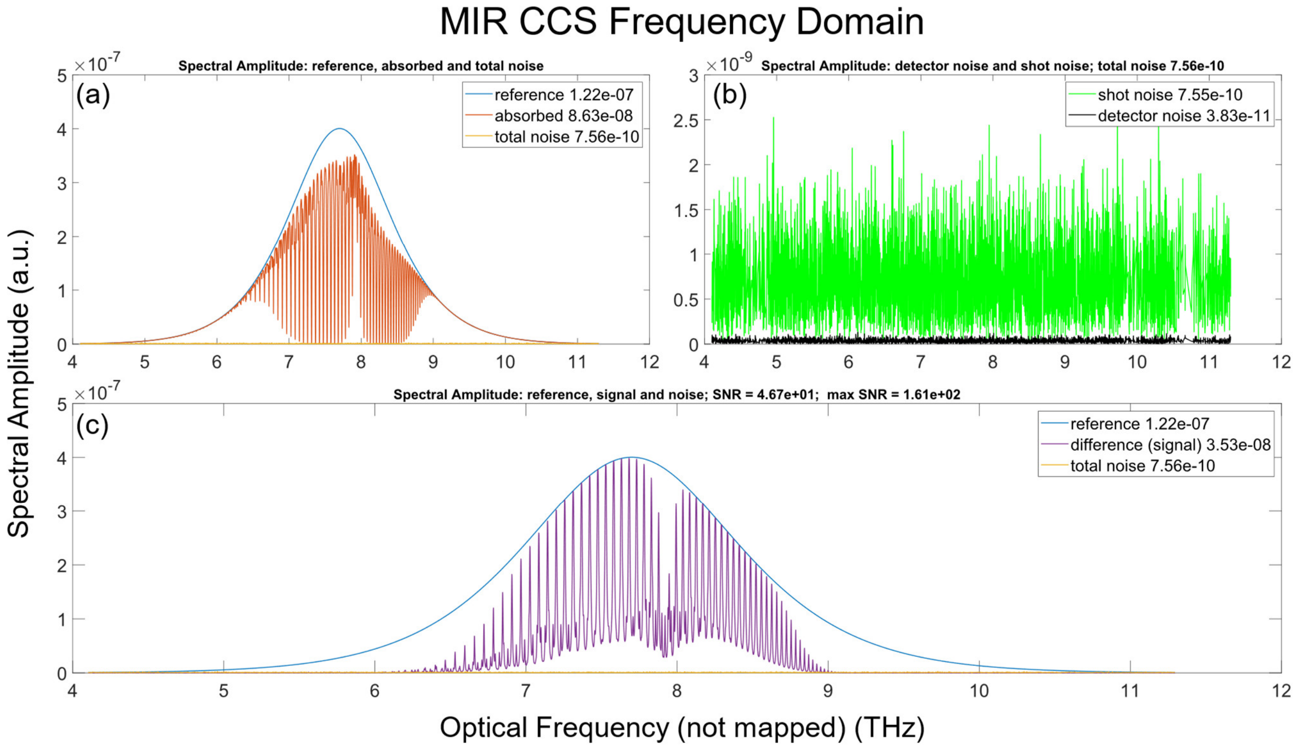

Supplementary Fig. 19| Frequency domain of MIR CCS. a. Spectral amplitudes of the ideal reference measurement, ideal absorbed measurement, and total noise. The average amplitudes of each spectrum are displayed in the legend box; the same is true in (b) and (c). **b.** Spectral amplitudes of shot noise and detector noise. **c.** Spectral amplitudes of the ideal reference measurement, ideal difference spectrum, and total noise. The spectral average SNR at this (unit) sample concentration (46.7) and its upper limit (161) are displayed in the plot subtitle.

5.4 CCS (with temporal filtering)

This detection scheme is different with two cases above, and its basic idea comes from a previous work on EOS[3]. We will cut the interferograms, both reference and absorbed, at a specific delay, τ_c ($\tau_c > 0$), and keep only the part $\tau > \tau_c$ for the detection of the sample. Since the center of the interferogram (around $\tau = 0$) will be cut out, the detection saturation there will be acceptable, and a larger SFG (by tuning target and/or local power) or readout power can be applied. In this example simulation, we keep the readout power the same and increase the target power tenfold and local power fivefold compared to the case (CCS without temporal filtering) in the last section. Next, a $\tau_c = 0.5\ ps$ is applied, the results of which in time domain and frequency domain are presented in Supplementary Figs. 20 and 21, respectively.

In contrast to the two previous cases, here the absorbed amplitude is generally larger than the reference amplitude (21 (a)). Although the estimated SNR can be significantly higher than the without-filter case, the signal obtained here is more useful for detecting the presence of the molecule than recovering its complete fingerprint. In other words, as part of the information is excluded by temporal filtering, the original and complete absorption spectrum of the sample cannot be retrieved, at least directly, like in general DCS and CCS.

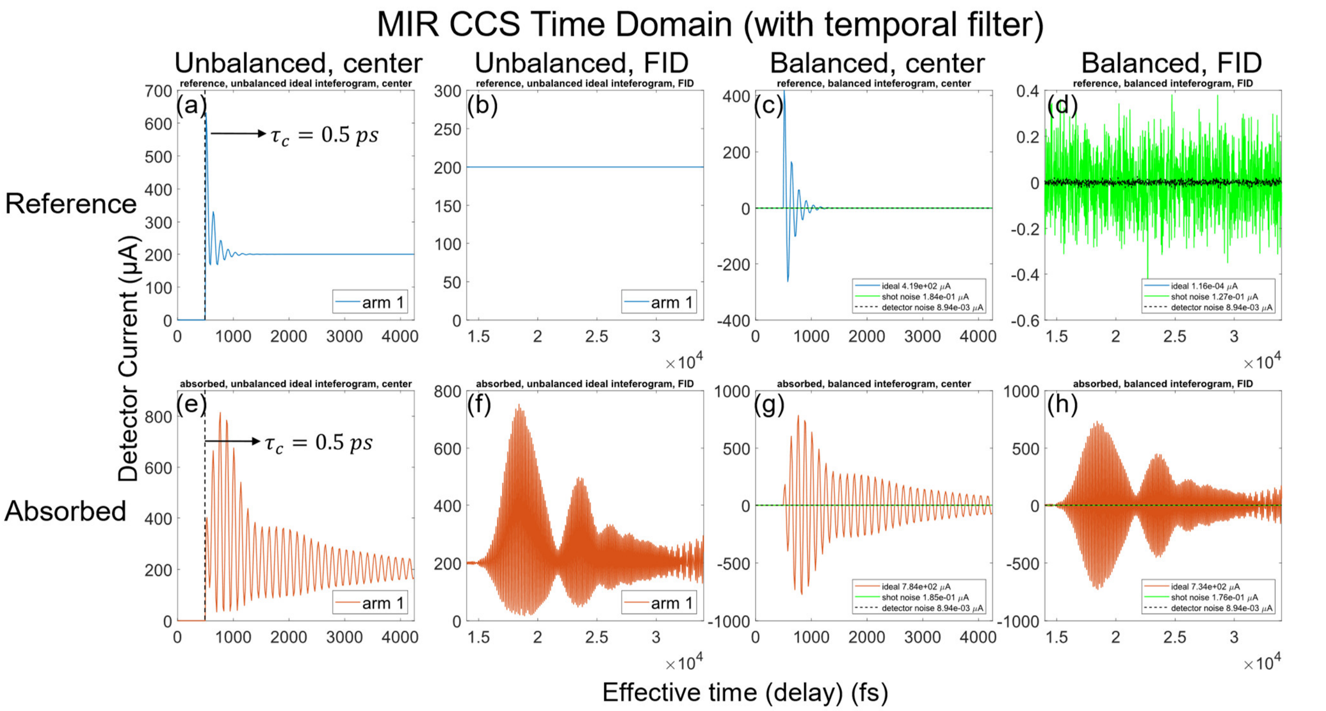

Supplementary Fig. 20| Time domain of MIR CCS (with temporal filtering). a. Unbalanced ideal interferogram at the center (0 ps – 4.25 ps) of the reference measurement (without sample), with a $\tau_c = 0.5\ ps$ (same for (e)). Note that only the signal recorded by one arm of the balanced detector is shown, and the signal at the other arm is of π-phase difference; the same is true for (b), (e), and (f). **b.** Unbalanced ideal interferogram at one part of FID (14 ps – 34 ps) of the reference measurement. **c.** Balanced ideal interferogram at the center of the reference measurement, together with noises. The maxima of the ideal interferogram, as well as the average level (standard deviation) of the noises, is denoted in the legend box for reference; the same is true in (d), (g) and (h). **d.** Balanced ideal interferogram at one part of FID of the reference measurement, together with noise. **e.** Unbalanced ideal interferogram at the center of the absorbed measurement (with sample). **f.** Unbalanced ideal interferogram at one part of FID of the absorbed measurement. **g.** Balanced ideal interferogram at the center of the absorbed measurement, together with noise. **h.** Balanced ideal interferogram at one part of FID of the absorbed measurement, together with noise.

Clearly, different settings of powers and τ_c can lead to different SNR results, and here we only show one possibility. A complete and systematic discussion and optimization of them could be useful but involved, so they are therefore beyond the scope of this section. Nevertheless, the choice of τ_c is still worth additional discussion. While a larger τ_c can further decrease reference amplitude (subtrahend), it also decreases the absorbed signal (minuend) since the FID signal generally decays exponentially in the time domain. In this example, the choice of $\tau_c = 0.5\ ps$ is a balance between the temporal amplitudes of the reference signal and absorbed signal, on one hand. On the other hand, at this given setting of power and concentration, this choice of timing ensures as much information is preserved as possible without saturating the detector. Moreover, though we highlight the FID signal from 14-34 ps throughout the paper, we do not use a τ_c near there in this simulation for two reasons. Firstly, despite the minimal residual reference signal there, the absorbed signal at such a large time delay is also weaker than that closer to $\tau = 0$ (see 21 (e)-(h)). A choice of τ_c that is too large, e.g., 10 ps, will eliminate a significant portion of useful signal, for example, signal from 0.5-4.25 ps as shown in panel (e). Secondly, and more importantly, such a distinct peak at such a large time delay is a unique feature of CO_2 (linear molecule)[6,19], which is special compared to more general molecules. Therefore, by using a τ_c much closer to 0 than that unique FID peak, we demonstrate that this method works, and our related claims hold, for more general cases and do not have to rely on such special features, although our theoretical model (Fig. 1e, Fig. 2d-e, and Supplementary Fig. 14) assumes a picture more like this feature for clarity.

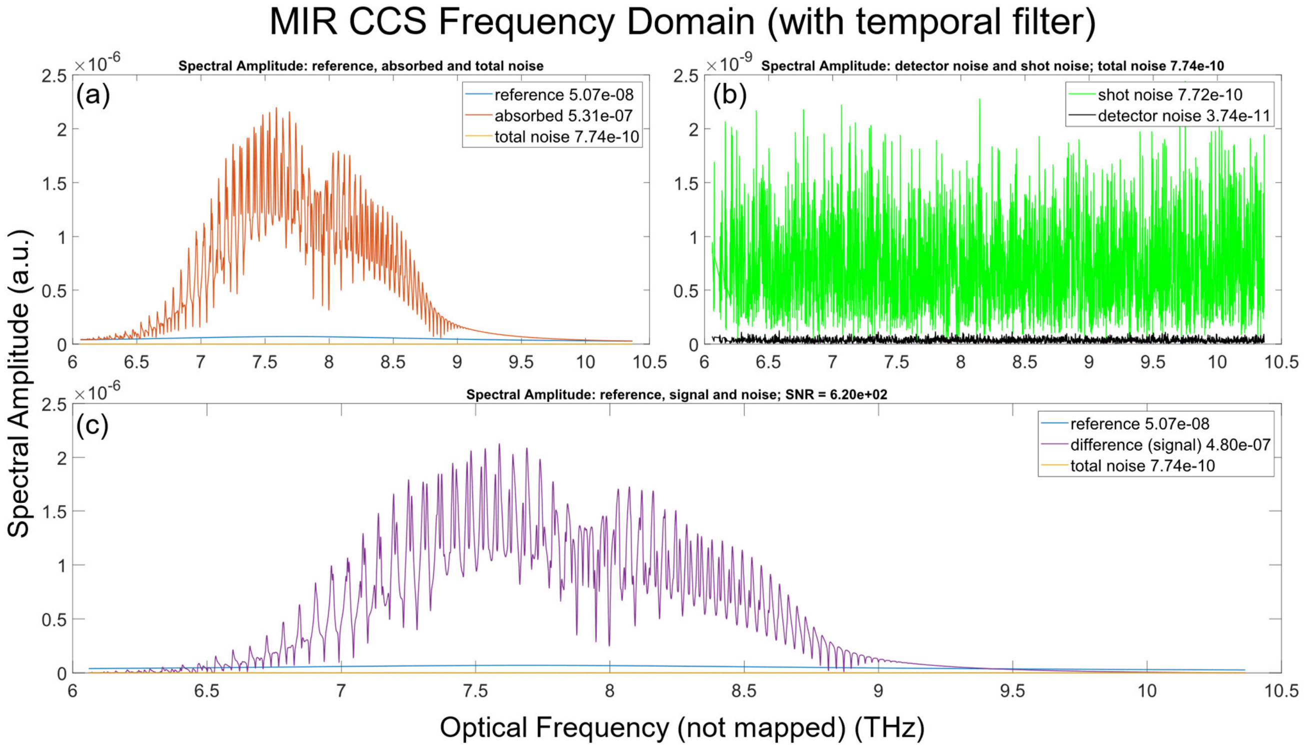

Supplementary Fig. 21| Frequency domain of MIR CCS (with temporal filtering). a. Spectral amplitudes of ideal reference measurement, ideal absorbed measurement, and total noise. The average amplitudes of each spectrum are displayed in the legend box; the same is true in b and c. **b.** Spectral amplitudes of the shot noise and detector noise. **c.** Spectral amplitudes of the ideal reference measurement, ideal difference spectrum, and total noise. The spectral average SNR at this (unit) sample concentration (620) is displayed in the subtitle.

5.5 Comparison

The trendlines between relative sample concentration and SNR for all three schemes are depicted in Supplementary Fig. 22. Each data point denotes the highest possible SNR that can be obtained at that concentration. When the SNR is greater than or equal to 1, we assume the sample (absorption) is detectable. Otherwise, it is assumed undetectable, as it would be hard to distinguish the spectral difference from noise. The abscissa (concentration) of the intersection between the line of SNR=1 and each curve can be understood as its sensitivity (minimum detectable concentration, MDC).

Let us first discuss DCS and CCS without temporal filtering. Although a different sample concentration could lead to a different absorbed measurement, their reference measurements, which already saturate their detectors, do not change. Therefore, at different concentrations, neither increasing nor decreasing the optical power would further optimize the SNR, so that we keep the same power setting for the SNR estimation at different concentrations. In other words, each data point in the curves denotes the highest possible SNR one may possibly get at the given concentration, which is limited by the detector saturation instead of the optical power. Compared to the unit concentration, a higher concentration might lead to a higher SNR because of a larger difference spectrum, until it approaches its upper limit, as explained in Section 5.2. A lower concentration would decrease the SNR all the way to zero, with an SNR<1 being regarded as undetectable. Since the SNR would decrease at a certain fixed rate, the sensitivity (MDC) is determined by the max SNR, which fundamentally depends on the detector.

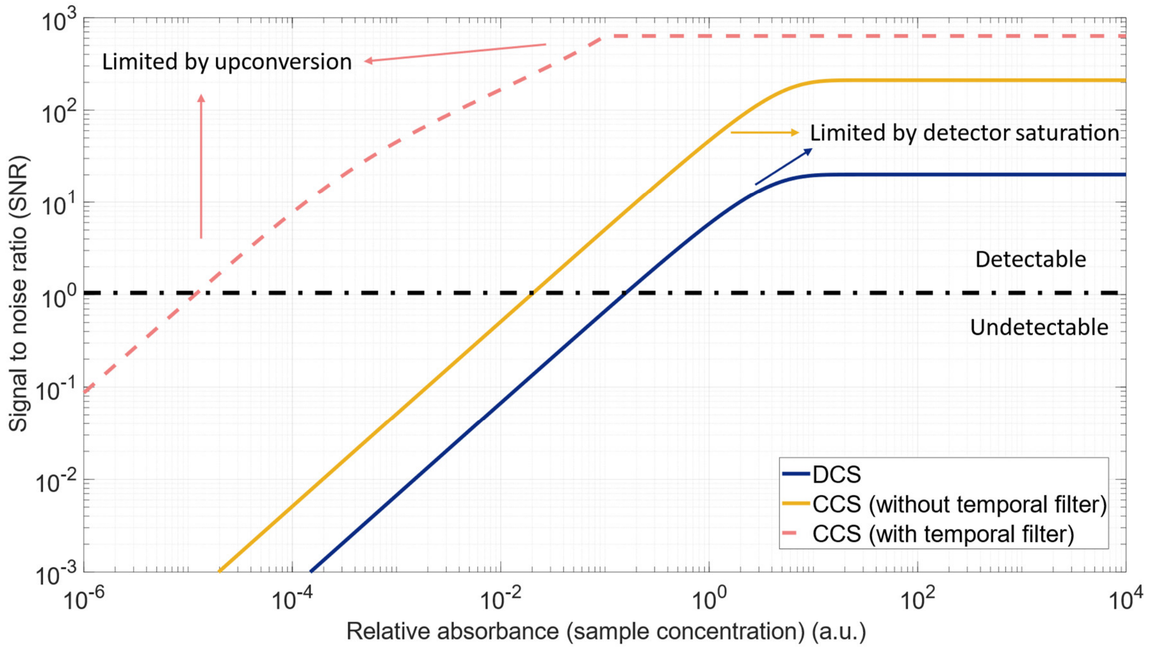

Supplementary Fig. 22| Scaling between relative concentration and SNR for different detection schemes. MIR DCS (blue solid line), MIR CCS without temporal filtering (yellow solid line), and MIR CCS with temporal filtering (pink dashed line). For MIR DCS and MIR CCS without temporal filtering, their highest SNR is limited by detector saturation, as is their sensitivity (minimum detectable concentration, the intersection between each curve and the line of SNR=1). For MIR CCS with temporal filtering, while its highest SNR is still limited by the detector saturation, its MDC is fundamentally limited by the strength of the nonlinearity, which determines where the SNR starts to decrease with the concentration.

Let us then discuss CCS with temporal filtering, which is slightly different. For a concentration higher than the unit concentration, the SNR could not notably increase despite a stronger FID tail, because the detector is already set to be saturated in the absorbed measurement at the unit concentration, and the spectral amplitude of the reference measurement is already very low. For a lower concentration, if we keep the same optical power and τ_c, the FID tail will get weaker, so the SNR will decrease. However, if we can apply a higher power (target or local) to compensate the lower absorption, we can keep the amplitude of FID signal the same and still saturate the detector. As such, the SNR is estimated to be the same as that at the unit concentration, which explains the part of the plateau extending to concentrations smaller than 10^0. This plateau can be kept until there is no more optical power (upconversion capability) available, after which the SNR will start to decrease with the sample concentration in a way similar to the other two cases. Therefore, unlike the other two cases, the sensitivity (MDC, the intersection) here is determined by the highest upconversion capability, which is decided by target power, local power, and nonlinear platform together, as claimed and discussed in the main paper. In this simulation, we assume the availability of roughly a factor of 10 higher nonlinear upconversion strength as compared to the parameters used at the unit concentration, to keep the SNR from decreasing until a concentration as low as 10^{-1} (the turning point). The practical values of the turning point and sensitivity will depend on the specific experimental conditions, for which a more accurate estimation would require a more accurate model of the nonlinear conversion process.

In summary, in this section, we demonstrate that the MIR CCS (without temporal filtering) can have a higher SNR and sensitivity compared to the MIR DCS, thanks to the advantages of NIR detectors and smaller noises due to the reduced background signal. In both cases, we show their SNR and sensitivity are fundamentally limited by the detector saturation and noise if high enough optical power is used, under our assumptions of the noise sources. Moreover, CCS with temporal filtering can provide even higher SNR and sensitivity because of its different detection methodology, which will be fundamentally decided by the upconversion capability instead of detector saturation. However, unlike the other two methods, it cannot provide

the full information of the absorption spectrum because some information is lost in the temporal filtering, though it can give a higher sensitivity for the detection of the presence of molecules.

References

1. Liu, M. *et al.* High-Power Mid-IR Few-Cycle Frequency Comb from Quadratic Solitons in an Optical Parametric Oscillator. *Laser & Photonics Reviews* 2200453 (2022) doi:10.1002/lpor.202200453.
2. Marandi, A., Leindecker, N. C., Pervak, V., Byer, R. L. & Vodopyanov, K. L. Coherence properties of a broadband femtosecond mid-IR optical parametric oscillator operating at degeneracy. *Opt. Express* **20**, 7255–7262 (2012).
3. Pupeza, I. *et al.* Field-resolved infrared spectroscopy of biological systems. *Nature* **577**, 52–59 (2020).
4. Chen, Z., Hänsch, T. W. & Picqué, N. Upconversion mid-infrared dual-comb spectroscopy. *arXiv:2003.06930 [physics]* (2020).
5. Zhu, Z., Ni, K., Zhou, Q. & Wu, G. Digital correction method for realizing a phase-stable dual-comb interferometer. *Opt. Express* **26**, 16813 (2018).
6. Lanin, A. A., Voronin, A. A., Fedotov, A. B. & Zheltikov, A. M. Time-domain spectroscopy in the mid-infrared. *Sci Rep* **4**, 6670 (2014).
7. Yokoyama, S., Yokoyama, T., Hagihara, Y., Araki, T. & Yasui, T. A distance meter using a terahertz intermode beat in an optical frequency comb. *Opt. Express* **17**, 17324–17337 (2009).
8. Stead, R. A., Mills, A. K. & Jones, D. J. Method for high resolution and wideband spectroscopy in the terahertz and far-infrared region. *J. Opt. Soc. Am. B* **29**, 2861–2868 (2012).
9. Coddington, I., Newbury, N. & Swann, W. Dual-comb spectroscopy. *Optica* **3**, 414–426 (2016).
10. Trebino, R. *Frequency-Resolved Optical Gating: The Measurement of Ultrashort Laser Pulses: The Measurement of Ultrashort Laser Pulses*. (Springer Science & Business Media, 2000).
11. Riek, C. *et al.* Direct sampling of electric-field vacuum fluctuations. *Science* **350**, 420–423 (2015).
12. Kowligy, A. S. *et al.* Infrared electric field sampled frequency comb spectroscopy. *Science Advances* **5**, eaaw8794 (2019).
13. Newbury, N. R., Coddington, I. & Swann, W. Sensitivity of coherent dual-comb spectroscopy. *Opt. Express* **18**, 7929–7945 (2010).
14. Demtröder, W. *Laser Spectroscopy: Basic Concepts and Instrumentation*. (Springer Science & Business Media, 2013).

15. Gordon, I. E. *et al.* The HITRAN2020 molecular spectroscopic database. *Journal of Quantitative Spectroscopy and Radiative Transfer* **277**, 107949 (2022).

16. Fathy, A., Sabry, Y. M., Hunter, I. W., Khalil, D. & Bourouina, T. Direct Absorption and Photoacoustic Spectroscopy for Gas Sensing and Analysis: A Critical Review. *Laser & Photonics Reviews* **n/a**, 2100556.

17. Weiner, A. *Ultrafast Optics*. (John Wiley & Sons, 2011).

18. Boyd, R. W. *Nonlinear Optics*. (Elsevier, 2008).

19. Griffiths, P. R. & De Haseth, J. A. *Fourier transform infrared spectrometry*. (Wiley-Interscience, 2007).
